# Synthesis and Anti-Proliferative Assessment of Triazolo-Thiadiazepine and Triazolo-Thiadiazine Scaffolds

**DOI:** 10.3390/molecules24244471

**Published:** 2019-12-06

**Authors:** Ahmed T. A. Boraei, Hazem A. Ghabbour, Mohamed S. Gomaa, El Sayed H. El Ashry, Assem Barakat

**Affiliations:** 1 Chemistry Department, Faculty of Science, Suez Canal University, Ismailia 41522, Egypt; 2 Department of Medicinal Chemistry, Faculty of Pharmacy, Mansoura University, Mansoura 35516, Egypt; hghabbour@mans.edu.eg; 3 Pharmaceutical Chemistry Department, College of Clinical Pharmacy, Imam Abdulrahman Bin Faisal University, Dammam 32241, Saudi Arabia; mohamdgomaa75@gmail.com; 4 Chemistry Department, Faculty of Science, Alexandria University, P.O. Box 426, Ibrahimia, Alexandria 21321, Egypt; eelashry60@hotmail.com; 5 Chemistry Department, College of Science, King Saud University, P.O. Box 2455, Riyadh, 11451, Saudi Arabia

**Keywords:** 4-Amino-1,2,4-triazolethione, Chalcone, PTSA, Thiadiazepine, HEPG-2, MCF-7, EGFR

## Abstract

A series of triazolo-thiadiazepines **4a**–**k** were synthesized with excellent yields using dehydrated PTSA as a catalyst in toluene. Two triazolo-thiadiazines were obtained; **8a** was formed directly by reflux in ethanol, whereas, PTSA promoted the formation of **8b**. The molecular structure of the formed triazolo-thiadiazepines is identical to the imine-form **4a**–**k** and not the enamine-tautomer **6a**–**k**. The structures of the newly synthesized triazolo-thiadiazepines **4a**–**k** and triazolo-thiadiazines **8a**–**b** were elucidated using NMR (^1^H, and ^13^C), 2D NMR, HRMS, and X-ray single crystal. Furthermore, **4a** was deduced using X-ray single crystal diffraction analysis. These new thiadiazepine hits represent an optimized series of previously synthesized indole-triazole derivatives for the inhibition of EGFR. The cytotoxicity activity against two cancer cell lines including human liver cancer (HEPG-2) and breast cancer (MCF-7) was promising, with IC_50_ between 12.9 to 44.6 µg/mL and 14.7 to 48.7 µg/mL for the tested cancer cell lines respectively, compared to doxorubicin (IC_50_ 4.0 µg/mL). Docking studies revealed that the thiadiazepine scaffold presented a suitable anchor, allowing good interaction of the various binding groups with the enzyme binding regions and sub-pockets.

## 1. Introduction

Indole and 1,2,4-triazoles heterocycles exhibit important biological activity and are present in many drugs [1,2]. The search for model drugs for different targets has led to the identification of ribavirin (an anti-viral drug), rizatriptan (used in the treatment of migraine headaches), and letrozole (for the treatment of hormonally-responsive breast cancer after surgery) [3,4,5].

**Figure 1:**
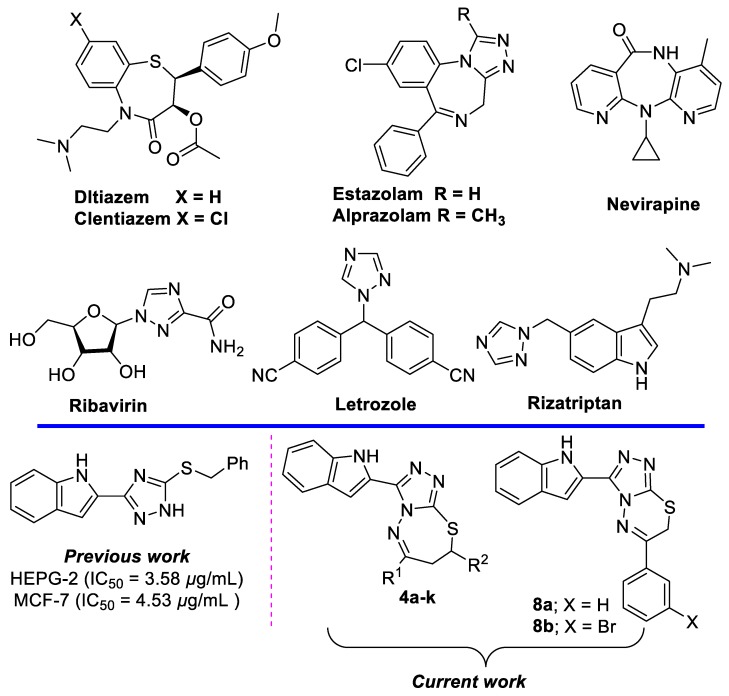
Selected drugs containing benzothiazepine, benzodiazepine, 1,2,4-triazole and indole scaffolds, and our designed new compounds.

Seven-membered heterocycles, like thiazepines, have occupied a unique place in pharmaceutical chemistry as they act as calcium channel blockers and are known to be applicable as cardiovascular drugs; these include diltiazem, clentiazem, and siratiazem [6,7,8,9]. Conversely, many examples of heterocycles have been reported with the benzodiazepine motif, such as estazolam, used for treatment of insomnia; alprazolam, commonly used for the treatment of panic disorder and anxiety disorders; flurazepam, which is anticonvulsant, anxiolytic and sedative, and enhanced the skeletal muscle relaxant responses; and nevirapine, used to treat HIV-1 infection and AIDS (Figure 1) [10,11]. 

Additionally, thiadiazepines are expected to play a vital role in the biological system. Recently, researchers have increased the focus towards the synthesis and biological importance of thiadiazepines [12,13,14,15,16].

The synthesis of annulated heterocyclic systems, which incorporate biologically active nuclei, is expected to show interesting new pharmacological and biological activities which are highly challenging. 

Recently, Boraei, A.T.A., et al., reported the cytotoxicity activity of a set of indole triazole derivatives, as potential EGFR inhibitors [17]. Therefore, we decided to combine the two previously discussed privileged structures as a potential scaffold to optimize the interaction of the indole-triazole series with the EGFR receptor.

Herein, PTSA was used as a catalyst for the synthesis of 11 indolo-triazolo-thiadiazepines and two indolo-triazolo-thiadiazenes, via reaction of 4-amino-1,2,4-triazole-3-thione with various chalcones and two phenacyl bromides. The newly introduced indolo-triazolo-thiadiazepine scaffolds, which possess better EGFR activity, selectivity, and greater potential, are promising for future preclinical and clinical development.

## 2. Results

### 2.1. Synthesis of **4a**–**k**

**Scheme 1:**
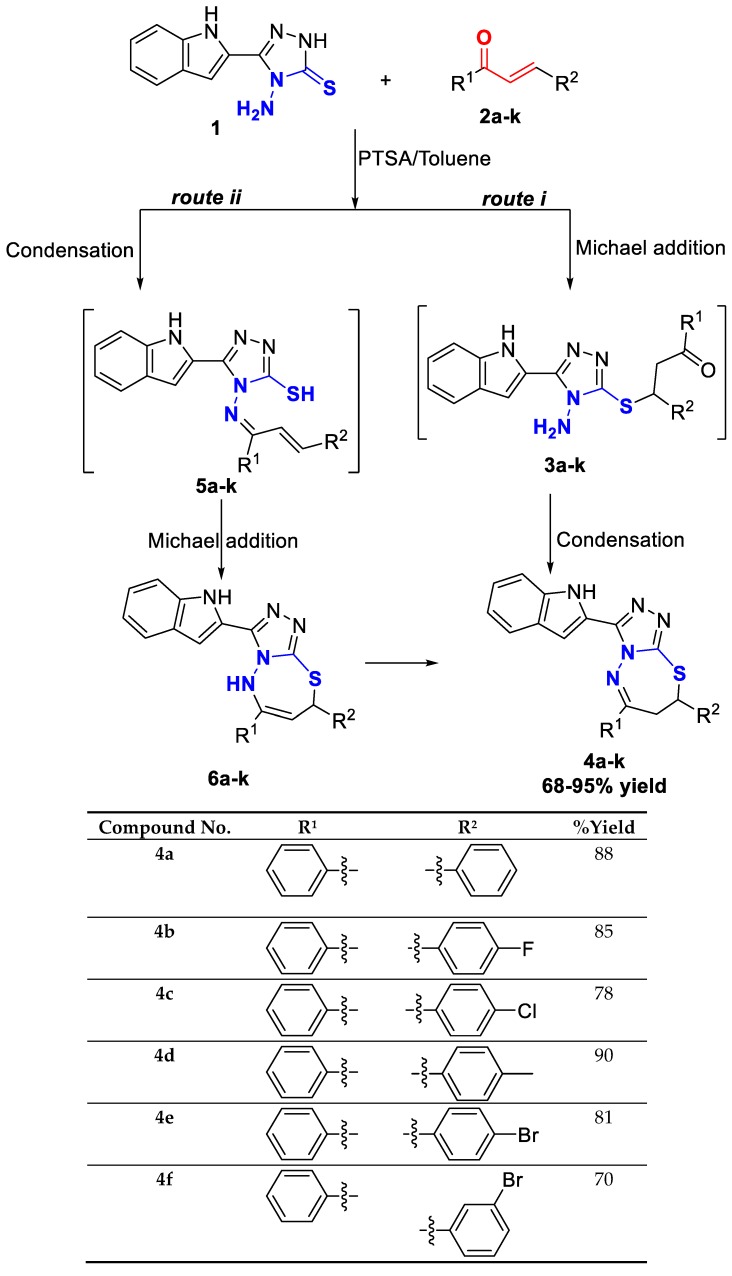

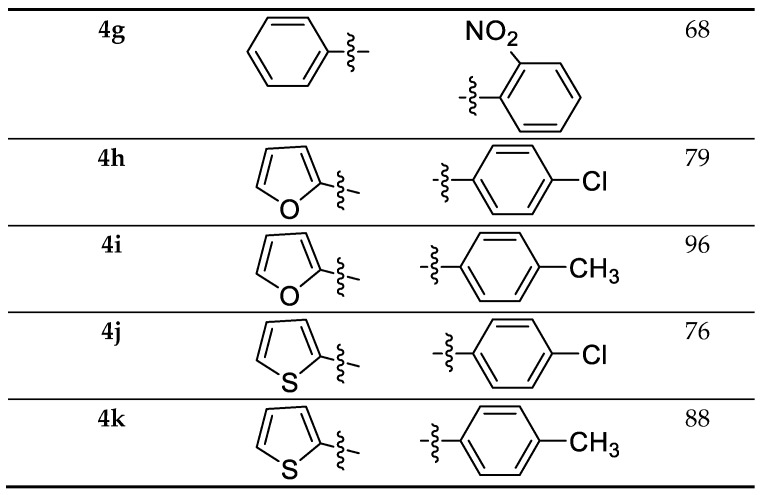
Synthesis and substrate scope of the target molecules **4a**–**k.**

The required precursor chalcones were synthesized via the reaction of aromatic aldehydes and ketones in the presence of either aqueous KOH or NaOH in ethanol [18]. Chalcones **2a**–**k** were reacted with 4-amino-1,2,4-triazole-3-thione having indole moiety **1,** using *p*-toluenesulfonic acid (*p*TsOH or PTSA) as a reaction promoter, in dry toluene for 30 min under reflux, to yield the target compounds **4a**–**k** with high yield (Scheme 1).

The mechanism of the reaction is expected to follow one of the following pathways: route (*i*), which involved Michael addition of the sulfhydryl group of the 4-amino-1,2,4-triazole-3-thione derivative **1** to the *α,β*-unsaturated carbonyl compounds **2a**–**k** [18] to form the intermediate *thia*-Michael adducts **3a**–**k**, followed by condensation and dehydration due to the intramolecular nucleophilic attack of the amino group on the carbonyl carbon, or route (*ii*), which involves, firstly, the removal of a water molecule due to the condensation of the amino and carbonyl groups to form the intermediate *aza*-dienes **5a**–**k**, and then a subsequent intramolecular conjugate addition of the sulfhydryl group to produce triazolo-thiadiazepine [19].

**Scheme 2:**
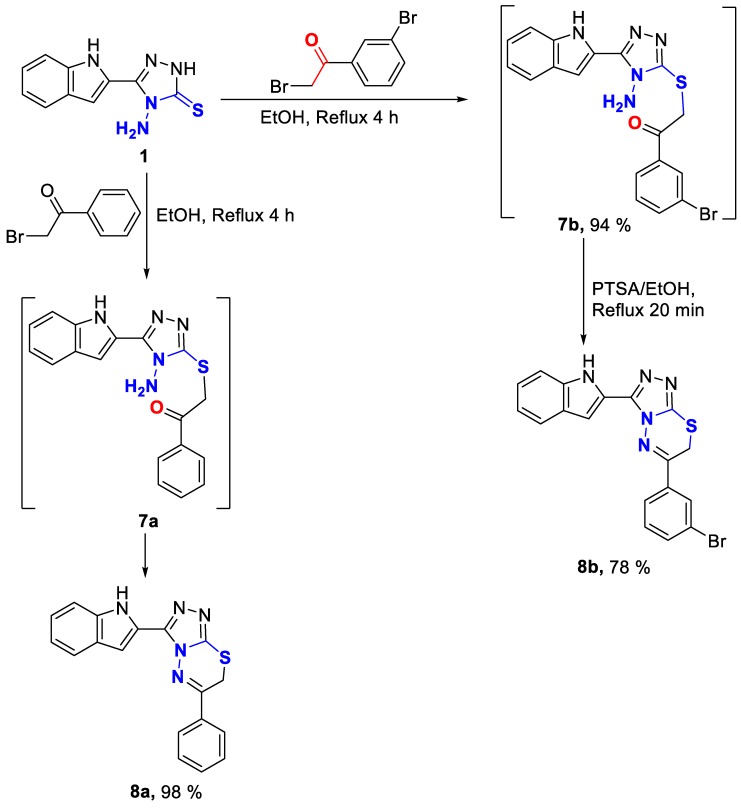
Synthesis of triazolo-thiadiazines **8a**–**b.**

Triazolo-thiadiazines **8a**, and **8b** were obtained via the reaction of 4-amino-1,2,4-triazole-3-thione derivative **1** with phenacyl bromide and *m*-bromophenacyl bromide, respectively. We noticed that in the case of phemacyl bromide, **8a** was obtained directly by reflux in ethanol for 4 h whereas, in the case of *m*-bromophenacyl bromide at the same conditions, the intermediate **7b** was isolated which then cyclized by reflux in the presence of PTSA in ethanol for 20 min to afford product **8b**. The formation of the intermediate **7b** is suggested to be due to the deactivation caused by the electron withdrawing effect of bromine atom (Scheme 2). 

#### 2.1.1. Structural Analysis 

^1^H NMR of the triazole-thiadiazepines **4a**–**k** showing that the three protons of the thiadiazepine ring are not equivalent and appeared mostly as doublet of doublets at different chemical shifts. For example, one of the methylene protons (described as trans proton) appeared as doublet of doublets around 3.45 ppm, with coupling constants *^2^J* ≈ 13.5 Hz and ^3^*J* ≈ 13.2 Hz, whereas, the second methylene proton (described as cis proton) appeared around 3.74 ppm with *^2^J* ≈ 13.5 Hz, and ^3^*J* ≈ 4.7 Hz; and the methine proton (CH) attached to the asymmetric carbon was identified between *δ* 5.33 and 5.60 ppm with a ^3^*J* around 13.0 and 4.7 Hz respectively. The remaining aromatic protons were observed between *δ* 7.00 and 8.30 ppm, while the indole NH was observed around *δ* 12.00 ppm. ^13^C NMR displayed the thiadiazepine methylene carbon (CH_2_) around *δ* 36.6 ppm and methine carbon (CH) around *δ* 53.5 ppm. The quaternary carbon resulted from the carbonyl condensation, appeared around *δ* 171.0 ppm, and the remaining aromatic carbons appeared between *δ* 104.0, and 148.0 ppm.

The NMR of **7b** showed the methylene protons (S-CH_2_-) at *δ* 4.30 ppm, and the amino group protons at *δ* 6.31 ppm. The methylene carbon (S-CH_2_-) appeared at *δ* 39.2 ppm, whereas the carbonyl carbon was found at *δ* 192.8 ppm.

NMR of triazolo-thiadiazines **8a**–**b** showed -SCH_2_- protons as singlets around *δ* 4.50 ppm, whereas the respective carbon was observed at *δ* 22.9 ppm. The thiadiazine quaternary carbon resulted from condensation appeared at *δ* 156.0 ppm in **8a** and *δ* 154.8 ppm in **8b**. The two triazole carbons in **8a**–**b** were assigned around *δ* 142.0, and 146.5 ppm. The indole NH proton was identified around *δ* 12.00 ppm. 

#### 2.1.2. X-Ray Diffraction Analysis

**Figure 2:**
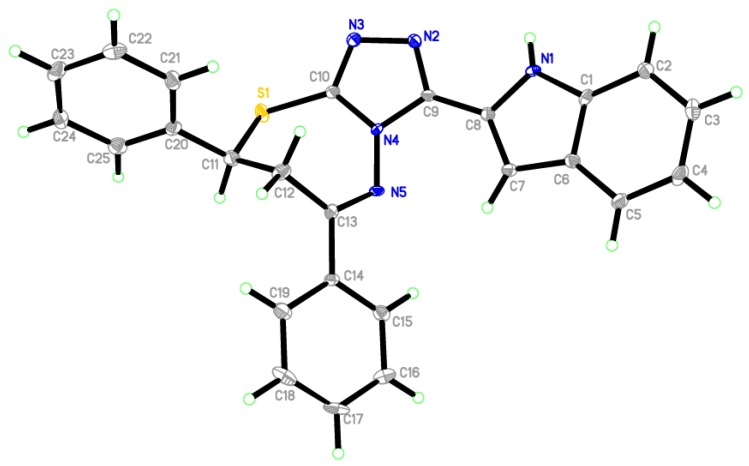
ORTEP of 4a.

Compound **4a** fortunately was obtained in a single crystal**,** which was solved using X-ray single crystal diffraction [20,21]. Table S1 lists the crystallographic data collection and structure refinement features. The later compound crystalized in a space group, *a* = 10.1159 (11) Å, *b* = 10.6507 (11) Å, *c* = 10.9680 (12) Å, *α* = 95.737 (3)°, *β* = 116.290 (3)°, *γ* = 100.618 (3)°, *V* = 1018.99 (19) Å^3^ and triclinic crystal system with *P*^−1^. Table S2 describes some bond angles and selected interatomic distances. The unit cell of the titled compound contained one molecule which appeared in the unit cell, showing that all bond angles and bond lengths of the benzene rings were in the ordinary framework. The indole ring (C1-C8/N1) formed a dihedral angle of 15.35° with the triazole ring (C9/N2/N3/C10C4)). The structural feature of **4a** as depicted in Figure 2 shows an imine functionality, and the C13=N5 bond is [1.296 (5) (15) Å]. In the molecular packing (Figure S1), molecules were connected via strong classical intermolecular hydrogen bonds N1—H1N1···N2^i^ (Table S3).

### 2.2. Anticancer Activity

The cytotoxic activity of the synthesized indolo-triazolo-thiadiazepines was tested in vitro against two cancer cell lines namely human liver cancer (HEPG-2), and breast cancer (MCF-7). The hits showed IC_50_ between 12.9 to 44.6 µg/mL and 14.7 to 48.7 µg/mL for the tested cancer cell lines respectively, compared to doxorubicin (IC_50_ 4.0 µg/mL) (Table 1). The triazolo-thiadiazepine ring notably improved the binding properties and subsequent antiproliferative activity, as observed from the low activity of compounds **8a** and **8b** compared to that of compounds such as **4b** and **4c**, which possessed the triazolo-thiadiazepine nucleus. Compounds having *para*-substituted phenyl with an electron withdrawing group, such as **4c**, **4e**, and **4b** were shown to have better activity than the *ortho* **4g** or *meta* **4f** or *para* **4d** with electron donating analogues. Interestingly, compounds bearing a heterocyclic substituent showed slightly better activity against HEPG-2 than their phenyl counterparts (Compounds **4i**, and **4j** compared to compounds **4c**, and **4d**), an observation that was not noted against the MCF-7 cell line, which could be explained by the difference in compound uptake by the two cancer cell types. The chemaxon-calculated LogP was found to be approximately 4.65 for the heterocyclic derivatives, and 5.62 for the phenyl derivatives, with no big difference in the pKa compounds. The possible difference in LogP compounds could have impacted the penetration through the cancer cells and favored the HEPG-2 cells in the case of the heterocyclic derivatives.

### 2.3. Molecular Modeling

We have previously reported the design and synthesis of indolyl-1,2,4-triazole-3-thione and its 3-benzylsulfanyl analogues as potent anticancer agents [17]. Herein, we are presenting a lead optimization through ring variation, ring expansion, and functional group extension techniques for EGFR inhibition. Ring expansion and variation with a triazolo-thiadiazepine ring was performed to increase the flexibility of the molecule and allow better interaction of the important binding groups with the enzyme sub pockets. The triazolo-thiadiazepine ring allowed better positioning of the substituents in the hydrophobic sub pocket and the cleft region. The triazolo-thiadiazepine ring had the advantage of being a spider-like scaffold, which enables the exploration and optimization of the substituents around the scaffold for optimum enzyme binding. It also allows for pharmacokinetic optimization and preclinical development.

**Figure 3:**
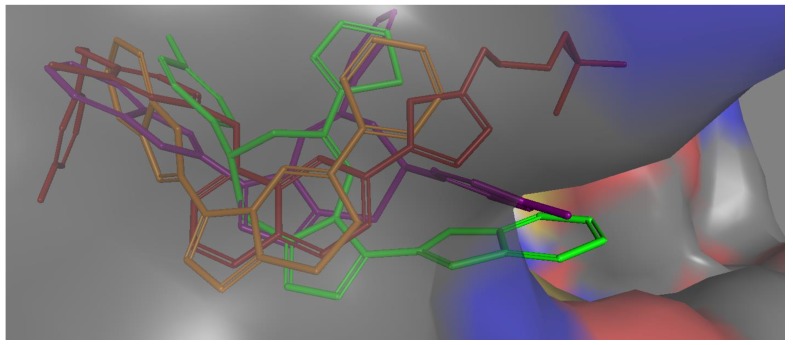
Molecular surface representation of compound **4b** purple, compound **4j** green, compound **8a** brown, and co-crystallized ligand (red) in EGFR active site (front view).

**Figure 4:**
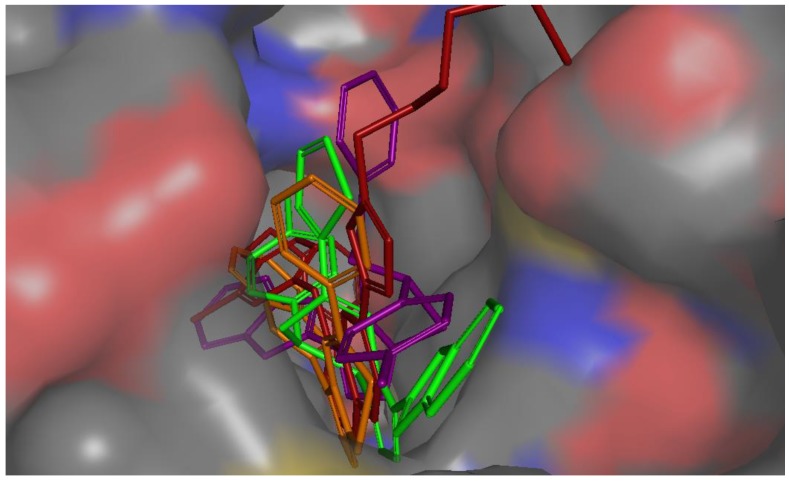
Molecular surface representation of compound **4b** purple, compound **4j** green, compound **8a** brown, and co-crystallized ligand (red) in EGFR active site (access channel view).

All active compounds showed poses that are comparable to the co-crystallized inhibitor and occupied the enzyme’s three main binding pockets (Figure 3 and Figure 4). The triazolo-thiadiazepine ring acts as an anchor that positions the compounds tightly in the active site through hydrogen bonding with key residue Met 793. The bicyclic, slightly flexible system also allowed for better interaction of the aryl and heteroaryl substituents, with the residues in the cleft and hydrophobic regions (Figure 3 and Figure 4). The *para*-halo substituted derivatives demonstrated better interaction through dipole–dipole bonds, while the *meta*-substituted, and *ortho*-substituted compounds were not able to form such an interaction. This partially explained the lower activity of the *ortho-*, and *meta*-substituted derivatives, as well as the *para*-methyl substituted derivatives. The binding scores were slightly better in the case of the phenyl derivatives than for the heterocyclic ones; however, this was not conclusive, and the difference in activity could not be explained based on the binding energy calculation. 

**Figure 5:**
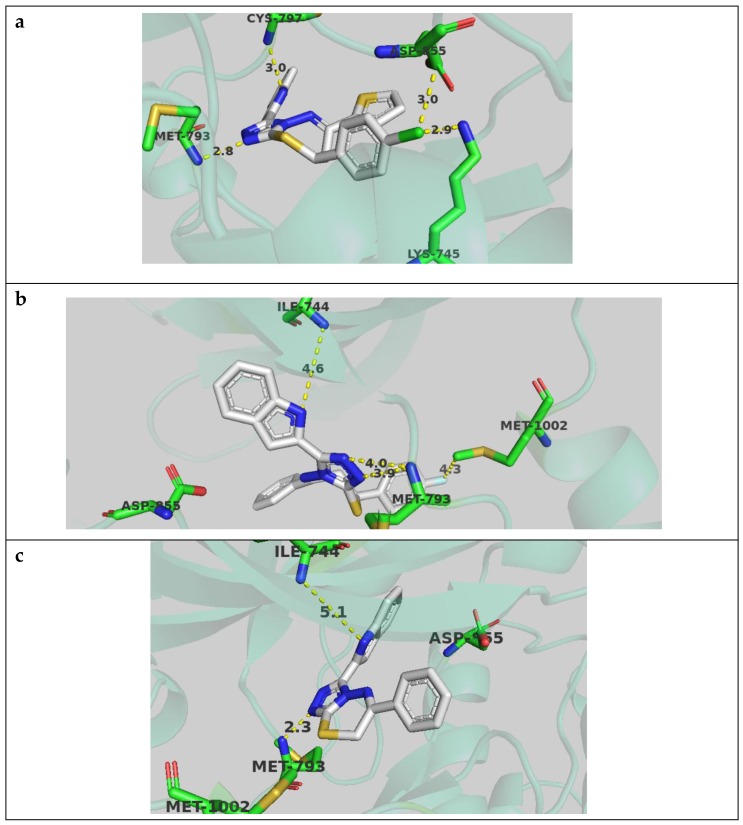
Compound **4j (a),** Compound **4b** (**b**), and compound **8a** (**c**) docked in EGFR active site and performing interactions with key residues. Distances are represented as yellow dotted lines and are in angstrom.

Compound **4j** docked well in the active site and showed an important hydrogen bond with the key Met 793 residue, with the triazole anchor at a distance of 2.8 angstrom. The *p*-chlorophenyl ring occupied the hydrophobic pocket and formed a dipole–dipole interaction with Lys 745 side chain. The indole ring was positioned well in the cleft region and formed a hydrogen bond with Cys 797 (Figure 5a).

Compound **4b** showed a similar pose at the active site but with the indole ring occupying the hydrophobic pocket, and exhibited potential hydrogen bonding with Ile 744 with the possible induced fit observed in biological systems. The *p*-fluorophenyl interacted with residues in the cleft region, such as Met 1002 (Figure 5b).

Compound **8a** had only one phenyl substituent in the thiadiazepine ring and showed comparable poses to other compounds, but with less interaction. The indole ring occupied the hydrophobic region and the triazole ring coordinated to Met 793, but lacked the interaction with the cleft region (Figure 5c).

In conclusion, the triazolo-thiadiazepine ring represents a good scaffold for EGFR inhibition, yielded active compounds that occupied all enzyme sub pockets, and has good potential for further optimization and development.

## 3. Materials and Methods 

### 3.1. Experiments: Chemistry

Melt-temp apparatus (SMP10) used for melting point (m.p.) determination in open capillaries and uncorrected. All reaction progress was monitored using TLC (eluent: *n*-hexane /ethyl acetate 4:6 for compounds **4a–k**; and CH_2_Cl_2_/MeOH 9:1 for the compounds **7b**, **8a** and **8b**) (TLC plates purchased from Merck, type: 60 F_254_/0.25 mm). The distillation technique was used for solvent purification if necessary. NMR (^1^H, ^13^C, and 2D) were recorded in DMSO-*d*_6_ using Brüker AC 300-600 spectrometers. Thermofinnigan MAT 95XP spectrometers used for molecular mass determination including HRMS spectral data determination.

### 3.2. Synthesis of Chalcones 

Chalcones were synthesized by stirring the appropriate aldehydes and active hydrogen ketones in ethanol, with a few drops of aq. NaOH (50%). The target chalcones were precipitated and subsequently underwent filtration and recrystallization from EtOH to give pure compounds [18]. 4-Amino-1,2,4-triazole-3-thione derivative **1** was prepared according to the procedures [22]. 

### 3.3. Synthesis of Thiadiazepine **4a**–**k**

The 4-amino-1,2,4-triazole-3-thione derivative **1** (1.0 mmol) and the suitable chalcones **2a–k** (1.1 mmol) were added to a previously dehydrated PTSA (3.0 mmol) in toluene and heated under reflux for 30 min. The organic layer was washed with water and aqueous solution of NaHCO_3_, followed by filtration, drying, and recrystallization from DMF/EtOH. 

*(E)-7,8-Dihydro-3-(1H-indol-2-yl)-6,8-diphenyl-[1,2,4]triazolo[3,4-b][1,3,4]thiadiazepine***4a** Yellow crystalline materials, m.p. 240–242 °C; ^1^H NMR (300 MHz) *δ* 3.45 (dd, 1 H, *J*_gem_ 13.7, *J*_trans_ 13.0 Hz, C*H*H_Thiadiazepin_), 3.66 (dd, 1 H, *J*_gem_ 13.7, *J*_cis_ 4.7 Hz, CH*H*_Thiadiazepin_), 5.33 (dd, 1 H, *J* 12.6, *J* 4.7 Hz, CH_7Thiadiazepin_), 7.00–7.70 (m, 13 H, H-3_Indol_, H-4_Indol_, H-5_Indol_, H-6_Indol_, H-7_Indol_, 8 H_Ph_), 8.23 (d, 2 H, J 7.1 Hz, 2 H_Ph_), 12.07 (br. s, 1H, NH_Indol_); ^13^C NMR (125 MHz) *δ* 36.6, 53.5, 104, 112.0, 119.8, 121.1, 123.3, 123.6, 126.6 127.6, 128.0, 128.1, 128.6, 129.2, 132.5, 133.8, 136.9, 142.3, 143.3, 147.5, 171.7; HRMS (FAB +ve) calcd for C_25_H_20_N_5_S (M + 1): 422.1439. Found: 422.1391.

*(E)-8-(4-Fluorophenyl)-3-(1H-indol-2-yl)-6-phenyl-7,8-dihydro-[1,2,4]triazolo[3,4-b][1,3,4]thiadiazepine***4b.** White solid product, m.p. 272–273 °C; ^1^H NMR (300 MHz) *δ* 3.47 (dd, 1 H, *J*_gem_ 13.7, *J*_trans_ 13.1 Hz, C*H*H_Thiadiazepin_), 3.67 (dd, 1 H, *J*_gem_ 13.7, *J*_cis_ 4.8 Hz, CH*H*_Thiadiazepin_), 5.37 (dd, 1 H, *J* 12.5, *J* 4.8 Hz, CH_7Thiadiazepin_), 7.00–7.70 (m, 12 H, H-3_Indol_, H-4_Indol_, H-5_Indol_, H-6_Indol_, H-7_Indol_, 7 H_Ph_), 8.24 (d, 2 H, *J* 7.2 Hz, 2 H_Ph_), 12.07 (br. s, 1 H, NH_Indol_); ^13^C NMR (75 MHz) *δ* 36.5, 52.7, 104.0, 112.0, 115.3, 115.5, 119.8, 121.1, 123.3, 123.6, 127.6, 128.1, 128.7, 128.8, 129.2, 132.5, 133.7, 136.9, 138.7, 143.2, 147.5, 171.7; HRMS (FAB^+^) calcd for C_25_H_19_N_5_SF (M + 1): 440.1345. Found: 440.1350.

*(E)-8-(4-Chlorophenyl)-7,8-Dihydro-3-(1H-indol-2-yl)-6-phenyl-[1,2,4]triazolo[3,4-b][1,3,4]thiadiazepine***4c.** White solid product, m.p. 271–272 °C; ^1^H NMR (300 MHz) *δ* 3.46 (dd, 1 H, *J*_gem_ 13.7, *J*_trans_ 13.2 Hz, C*H*H_Thiadiazepin_), 3.66 (dd, 1 H, *J*_gem_ 13.7, *J*_cis_ 4.7 Hz, CH*H*_Thiadiazepin_), 5.35 (dd, 1 H, *J* 12.6, *J* 4.7 Hz, CH_7Thiadiazepin_), 7.00–7.73 (m, 13 H, H-3_Indol_, H-4_Indol_, H-5_Indol_, H-6_Indol_, H-7_Indol_, 8 H_Ph_), 8.24 (d, 2 H, *J* 7.1 Hz, 2 H_Ph_), 12.07 (br. s, 1H, NH_Indol_); ^13^C NMR (75 MHz) *δ* 36.3, 52.7, 104.1, 112.0, 119.8, 121.1, 123.4, 123.6, 127.6, 128.1, 128.6, 129.3, 132.5, 132.6, 132.7, 136.9, 141.5, 143.1, 147.6, 171.7; HRMS (EI) calcd for C_25_H_18_N_5_SCl (M): 455.0971. Found: 455.0960.

*(E)-8-(p-Tosyl)-7,8-dihydro-3-(1H-indol-2-yl)-6-phenyl-[1,2,4]triazolo[3,4-b][1,3,4]thiadiazepine***4d.** Yellowish white needles, m.p. 263–264 °C; ^1^H NMR (400 MHz) *δ* 2.31 (s, 3 H, CH_3_), 3.44 (dd, 1 H, *J*_gem_ 13.6, *J*_trans_ 12.8 Hz, C*H*H_Thiadiazepin_), 3.64 (dd, 1 H, *J*_gem_ 13.6, *J*_cis_ 4.8 Hz, CH*H*_Thiadiazepin_), 5.31 (dd, 1 H, *J* 12.6, *J* 5.2 Hz, CH_7Thiadiazepin_), 7.03–7.07 (m, 2 H, H-3_Indol_, H-5_Indol_), 7.19–7.23 (m, 3 H, H-6_Indol_, 2 H_Ph_), 7.41 (d, 2 H, *J* 8.0 Hz, 2 H_Ph_), 7.51 (d, 1 H, *J*_6,7_ 8.0 Hz, H-7_Indol_), 7.64–7.74 (m, 4 H, H-4_Indol_, 3 H_Ph_), 8.23 (d, 2 H, *J* 8.0 Hz, 2 H_Ph_), 12.07 (br. s, 1 H, NH_Indol_); ^13^C NMR (100 MHz) *δ* 20.7, 36.7, 53.4, 104.0, 112.0, 119.8, 121.1, 123.3, 123.6, 126.5, 127.6, 128.0, 129.1, 129.3, 132.5, 133.8, 136.9, 137.5, 139.5, 143.4, 147.5, 171.7; HRMS (EI) calcd for C_26_H_21_N_5_S (M): 435.1518. Found: 435.1529.

*(E)-8-(4-Bromophenyl)-7,8-dihydro-3-(1H-indol-2-yl)-6-phenyl-[1,2,4]triazolo[3,4-b][1,3,4]thiadiazepine***4e.** Yellow crystalline materials, m.p. 271–272 °C; ^1^H NMR (300 MHz) *δ* 3.49 (dd, 1 H, *J*_gem_ 13.8, *J*_trans_ 13.5 Hz, C*H*H_Thiadiazepin_), 3.65 (dd, 1 H, *J*_gem_ 13.8, *J*_cis_ 4.5 Hz, CH*H*_Thiadiazepin_), 5.35 (dd, 1 H, *J* 12.6, *J* 4.5 Hz, CH_7Thiadiazepin_), 7.00–7.04 (m, 2 H, H-3_Indol_, H-5_Indol_), 7.19 (dd, 1 H, *J*_5,6_ 7.2, *J*_6,7_ 8.4 Hz, H-6_Indol_), 7.46–7.49 (m, 3 H, H-7_Indol_, 2 H_Ph_), 7.58–7.71 (m, 6 H, H-4_Indol_, 5 H_Ph_), 8.24 (dd, 1 H, *J* 7.2 Hz, 2 H_Ph_), 12.06 (br. s, 1H, NH_Indol_); ^13^C NMR (100 MHz) *δ* 36.2, 52.8, 104.1, 112.0, 119.8, 121.1, 121.2, 123.4, 123.7, 127.6, 12.1, 129.9, 129.3, 131.5, 132.6, 133.7, 136.9, 141.9, 143.2, 147.6, 171.7; CHN-microanalysis calcd for C_25_H_18_N_5_BrS: 60.00% C, 3.63% H, 14.00% N, 6.41% S. Found: 59.29% C, 4.13% H, 14.80% N, 6.69% S.

*(E)-8-(3-Bromophenyl)-7,8-dihydro-3-(1H-indol-2-yl)-6-phenyl-[1,2,4]triazolo[3,4-b][1,3,4]thiadiazepine***4f.** Yellow crystalline materials, m.p. 263–265 °C; ^1^H NMR (300 MHz) *δ* 3.54 (dd, 1 H, *J*_gem_ 13.8, *J*_trans_ 13.5 Hz, C*H*H_Thiadiazepin_), 3.69 (dd, 1 H, *J*_gem_ 13.8, *J*_cis_ 4.8 Hz, CH*H*_Thiadiazepin_), 5.32 (dd, 1 H, *J* 12.6, *J* 4.8 Hz, CH_7Thiadiazepin_), 7.00–7.05 (m, 2 H, H-3_Indol_, H-5_Indol_), 7.19 (dd, 1 H, *J*_5,6_ 7.2, *J*_6,7_ 8.4 Hz, H-6_Indol_), 7.36 (dd, 1 H, *J* ≈ 7.8 Hz, H_Ph_), 7.49 (d, 1 H, *J*_6,7_ 8.4 Hz, H-7_Indol_), 7.53 (d, 2 H, *J* 7.8 Hz, 2 H_Ph_), 7.62–7.76 (m, 5 H, H-4_Indol_, 4 H_Ph_), 8.26 (d, 2 H, *J* 7.2 Hz, 2 H_Ph_), 12.07 (br. s, 1H, NH_Indol_); ^13^C NMR (100 MHz) *δ* 35.9, 52.7, 104.0, 112.0, 119.8, 121.1, 121.8, 123.3, 123.6, 125.8, 127.6, 128.1, 129.3, 129.5, 130.8, 130.9, 132.6, 133.7, 136.9, 143.0, 145.0, 147.6, 171.6; CHN-microanalysis calcd for C_25_H_18_N_5_BrS: 60.00% C, 3.63% H, 14.00% N, 6.41% S. Found: 59.93% C, 3.87% H, 14.02% N, 6.25% S. 

*(E)-7,8-Dihydro-3-(1H-indol-2-yl)-8-(2-nitrophenyl)-6-phenyl-[1,2,4]triazolo[3,4-b][1,3,4]thiadiazepine***4g.** Yellow crystalline materials, m.p. 270–272 °C; ^1^H NMR (300 MHz) *δ* 3.61 (dd, 1 H, *J*_gem_ 13.8, *J*_trans_ 13.5 Hz, C*H*H_Thiadiazepin_), 3.76 (dd, 1 H, *J*_gem_ 13.8, *J*_cis_ 4.5 Hz, CH*H*_Thiadiazepin_), 5.60 (dd, 1 H, *J* 12.9, *J* 4.7 Hz, CH_7Thiadiazepin_), 7.01–7.07 (m, 2 H, H-3_Indol_, H-5_Indol_), 7.20 (dd, 1 H, *J*_5,6_ 7.2, *J*_6,7_ 8.1 Hz, H-6_Indol_), 7.49 (d, 1 H, *J*_6,7_ 8.1 Hz, H-7_Indol_), 7.59–7.73 (m, 5 H, H-4_Indol_, 4 H_Ph_), 7.83 (dd, 1 H, *J* 7.8, *J* 7.5 Hz, H_Ph_), 7.99 (d, 1 H, *J* 7.8 Hz, H_Ph_), 8.06 (d, 1 H, *J* 8.1 Hz, H_Ph_), 8.32 (d, 2 H, *J* 7.2 Hz, 2 H_Ph_), 12.09 (br. s, 1H, NH_Indol_); ^13^C NMR (100 MHz) *δ* 35.3, 48.7, 104.1, 112.0, 119.8, 121.8, 123.4, 123.6, 124.7, 127.6, 128.1, 128.5, 129.3, 132.8, 133.6, 134.4, 136.9, 137.3, 143.7, 146.7, 147.6, 171.4; HRMS (EI) calcd for C_25_H_18_N_5_O_2_S (M^+^): 466.1212. Found: 466.1233.

*(E)-8-(4-Chlorophenyl)-6-(furan-2-yl)-7,8-Dihydro-3-(1H-indol-2-yl)-[1,2,4]triazolo[3,4-b][1,3,4]thiadiazepine***4h.** Yellowish white solid product, m.p. 275–276 °C; ^1^H NMR (400 MHz) *δ* 3.38–3.44 (m, 1 H, C*H*H_Thiadiazepin_), 3.51 (dd, 1 H, *J*_gem_ 14.0, *J*_cis_ 5.2 Hz, CH*H*_Thiadiazepin_), 5.35 (dd, 1 H, *J* 12.0, *J* 5.2 Hz, H-7_Thiadiazepin_), 6.85–6.86 (m, 1 H, H-4_Furyl_), 7.033 (dd, 1 H, *J*_4,5_ 8.0, *J*_5,6_ 7.6 Hz, H-5_Indol_), 7.08 (s, 1 H, H-3_Indol_), 7.19 (dd, 1 H, *J*_5,6_ 7.6, *J*_6,7_ ≈ 8.0 Hz, H-6_Indol_), 7.44–7.53 (m, 5 H, H-7_Indol_, 4 H_Ph_), 7.62 (d, 1 H, *J*_4,5_ 8.0 Hz, H-4_Indol_), 7.71 (d, 1 H, *J* 3.6 Hz, H-3_Furyl_), 8.16 (s, H, H-5_Furyl_), 12.01 (br. s, 1H, NH_Indol_); ^13^C NMR (100 MHz) *δ* 35.9, 52.8, 104.1, 112.0, 113.3, 119.5, 119.9, 121.1, 123.4, 123.6, 127.6, 128.6, 128.7, 132.7, 136.9, 141.2, 143.2, 147.5, 148.3, 148.7, 161.3; HRMS (EI) calcd for C_23_H_16_N_5_OSCl (M^+^): 445.0764. Found: 445.0764.

*(E)-6-(Furan-2-yl)-8-7,8-dihydro-3-(1H-indol-2-yl)-8-p-tolyl-[1,2,4]triazolo[3,4-b][1,3,4]thiadiazepine***4i.** Brown solid product, m.p. 256–257 °C; ^1^H NMR (400 MHz) *δ* 2.29 (s, 3 H, CH_3_), 3.33–3.50 (m, 2 H, H-6_Thiadiazepin_, H-6′_Thiadiazepin_,), 5.20 (dd, 1 H, *J* 12.0, *J* 5.2 Hz, H-7_Thiadiazepin_), 6.84-6.85 (m, 1 H, H-4_Furyl_), 7.03 (dd, 1 H, *J*_4,5_ 8.0, *J*_5,6_ 7.6 Hz, H-5_Indol_), 7.09 (s, 1 H, H-3_Indol_), 7.16–7.21 (m, 3 H, H-6_Indol_, 2 H_Ph_), 7.35 (d, 2 H, *J* 8.0 Hz, 2 H_Ph_), 7.49 (d, 1 H, *J*_6,7_ 8.4 Hz, H-7_Indol_), 7.62 (d, 1 H, *J*_4,5_ 8.0 Hz, H-4_Indol_), 7.66 (d, 1 H, *J* 3.2 Hz, H-3_Furyl_), 8.15 (s, 1 H, H-5_Furyl_), 12.01 (br. s, 1H, NH_Indol_); ^13^C NMR (100 MHz) *δ* 20.7, 36.3, 53.5, 104.1, 112.0, 113.3, 119.2, 119.9, 121.1, 123.4, 123.6, 126.6, 127.6, 129.2, 136.9, 137.6, 139.3, 143.5, 147.5, 148.4, 148.6, 161.4; HRMS (EI) calcd for C_24_H_19_N_5_OS (M^+^): 425.1310. Found: 425.1327.

*(E)-8-(4-Chlorophenyl)-7,8-Dihydro-3-(1H-indol-2-yl)-6-(thiophen-2-yl)-* [1,2,4]*triazolo[3,4-b][1,3,4]thiadiazepine* **4j.** Yellow powder product, m.p. 280–281 °C; ^1^H NMR (300 MHz) *δ* 3.48 (dd, 1 H, *J*_gem_ 13.8, *J*_trans_ 12.6 Hz, C*H*H_Thiadiazepin_), 3.70 (dd, 1 H, *J*_gem_ 13.5, *J*_cis_ 5.1 Hz, CH*H*_Thiadiazepin_), 5.35 (dd, 1 H, *J* 12.0, *J* 4.8 Hz, H-7_Thiadiazepin_), 7.01-7.55 (m, 9 H, 1 H-4_Thiophen_, H-3_Indol_, H-5_Indol_, H-6_Indol_, H-7_Indol_, 4 H_Ph_), 7.61 (d, 1 H, *J*_4,5_ 7.8 Hz, H-4_Indol_), 8.04 (d, 1 H, *J* 4.8 Hz, H-3_Thiophen_), 8.10 (d, 1 H, *J* 3.3 Hz, H-5_Thiophen_), 12.04 (br. s, 1H, NH_Indol_); HRMS (EI) calcd for C_23_H_16_N_5_S_2_Cl (M^+^): 461.0536. Found: 461.0559.

*(E)-8-7,8-Dihydro-3-(1H-indol-2-yl)-6-(thiophen-2-yl)-8-p-tolyl-[1,2,4]triazolo[3,4-b][1,3,4]thiadiazepine***4k.** White solid, m.p. 277–278 °C; ^1^H NMR (400 MHz) *δ* 2.13 (s, 3 H, CH_3_), 3.44 (dd, 1 H, *J*_gem_ 13.2, *J*_trans_ 12.8 Hz, 6_Thiadiazepin_,), 3.65 (dd, 1 H, *J*_gem_ 13.8, *J*_cis_ 4.8 Hz, 6′_Thiadiazepin_,)), 5.20 (dd, 1 H, *J* 12.0, *J* 4.8 Hz, H-7_Thiadiazepin_), 7.01-7.33 (m, 6 H, H-3_Indol_, H-5_Indol_, H-6_Indol_, 2 H_Ph_, H_Thiophen_), 7.38 (d, 2 H, *J* 8.0 Hz, 2 H_Ph_), 7.48 (d, 1 H, *J*_6,7_ 8.4 Hz, H-7_Indol_), 7.61 (d, 1 H, *J*_4,5_ 7.6 Hz, H-4_Indol_), 8.02-8.06 (m, 2 H, H-3_Thiophen_, H-5_Thiophen_), 12.04 (br. s, 1H, NH_Indol_); ^13^C NMR (100 MHz) *δ* 20.7, 36.9, 53.4, 104.0, 112.0, 119.9, 121.1, 123.4, 123.7, 126.7, 128.9, 129.1, 134.1, 134.1, 136.9, 137.6, 138.5, 139.3, 143.6, 147.3, 166.8; HRMS (EI) calcd for C_24_H_19_N_5_S_2_ (M^+^): 441.1082. Found: 441.1078.

### 3.4. Procedure for **8a**,**b**

A mixture of 4-amino-1,2,4-triazole-3-thione derivative **1** (1.0 mmol) and the relevant phenacyl bromide (1.1 mmol) was refluxed for 4 h in 10 mL ethanol and left to cool to room temperature. The formed ppt was collected by filtration, dried and recrystallized from DMF/EtOH to give **8a**, and **7b** respectively.

### 3.5. Cyclization of **7b**

A mixture of **7b** (1.0 mmol) and dehydrated PTSA (1.0 mmol) was refluxed in ethanol for 20 min and then left to cool, then the ppt was filtered, washed with water and aqueous sodium hydrogen carbonate, dried, and recrystallized from DMF/EtOH to give **8b**. 

*4-Amino-3-(3-bromoacetophenonylsulfanyl)-5-(1H-indol-2-yl)-1,2,4-triazole***7b.** Yellow crystals, m.p. 267–268 °C; ^1^H NMR (300 MHz) δ δ 4.93 (SCH_2_), 6.31 (s, 2 H, NH_2_), 7.03 (dd, 1 H, *J*_4,5_ 8.1, *J*_5,6_ 7.2 Hz, H-5_Indol_), 7.16 (dd, 1H, *J*_5,6_ 7.2, *J*_6,7_ 8.1 Hz, H-6_Indol_), 7.29 (s, 1 H, H-3_Indol_), 7.45 (d, 1 H, *J*_6,7_ 8.1 Hz, H-7_Indol_), 7.54 (dd, 1 H, *J* 7.8 Hz, H_Ph_), 7.61 (d, 1H, *J*_4,5_ 7.8 Hz, H-4_Indol_), 7.87–7.90 (m, 1 H, H_Ph_), 8.04 (d, 1 H, *J* 7.8 Hz, H_Ph_), 8.19 (s, 1 H, H_Ph_), 11.70 (br. s, 1 H, NH_Indol_); ^13^C NMR (75 MHz) δ 39.2, 102.4, 111.9, 119.6, 120.8, 122.2, 122.9, 123.9, 127.4, 127.6, 131.0, 131.0, 136.2, 136.5, 137.6, 149.4, 152.6, 192.8; LRMS (EI) *m/z* (Int. %): 63.1 (12.4), 82.9 (33.5), 84.9 (26.8), 142.0 (100), 143.1 (21.0), 182.9 (23.5), 185.0 (14.8), 227.1 (19.1), 228.1 (25.6), 244.1 (14.28), 409.0 (70), 410.0 (21.7), 411.0 (71), 412.0 (18.9), 427.1 (4), 429.1 (4.63); HRMS (EI) calcd for C_18_H_12_N_5_BrS (M-H_2_O): 408.9984. Found: 408.9997.

*7H-3-(1H-Indol-2-yl)-6-phenyl-1,2,4-triazolo[3,4-b][1,3,4-thiadiazine***8a.** Brownish yellow crystalline materials, m.p. 269–271 °C; ^1^H NMR (400 MHz) *δ* 4.49 (s, 2 H, CH_2Thiadiazin_), 7.05 (dd, 1 H, *J*_4,5_ 7.6, *J*_5,6_ 7.3 Hz, H-5_Indol_), 7.20 (dd, 1H, *J*_5,6_ 7.3, *J*_6,7_ 8.1 Hz, H-6_Indol_), 7.25 (d, 1 H, *J* 1.1 Hz, H-3_Indol_), 7.48 (d, 1 H, *J*_6,7_ 8.1 Hz, H-7_Indol_), 7.61–7.69 (m, 4 H, H-4_Indol_, 3 H_Ph_), 8.10–8.13 (m, 2 H, 2 H_Ph_), 11.97 (br. s, 1 H, NH_Indol_); ^13^C NMR (100 MHz) *δ* 22.9, 103.8, 111.9, 119.7, 121.2, 123.3123.3, 127.6, 127.7, 129.2, 132.0, 133.5, 136.9, 142.1, 146.6, 156.0; HRMS (EI) calcd for C_18_H_13_N_5_S (M^+^): 331.0892. Found: 331.0888.

*6-(3-Bromophenyl)-3-(1H-indol-2-yl)-7H-[1,2,4]triazolo[3,4-b][1,3,4]thiadiazine***8b.** Yellow crystals, m.p. 252–253 °C; ^1^H NMR (600 MHz) δ 4.48 (s, 2 H, CH_2Thiadiazin_), 7.05 (dd, 1 H, *J*_4,5_ 7.8, *J*_5,6_ 7.2 Hz, H-5_Indol_), 7.19-7.21 (m, 2 H, H-3_Indol_, H-6_Indol_), 7.48 (d, 1 H, *J*_6,7_ 7.8 Hz, H-7_Indol_), 7.60 (dd, 1 H, *J* 7.8, *J* 8.4 Hz, H_Ph_), 7.67 (d, 1 H, *J*_4,5_ 7.8 Hz, H-4_Indol_), 7.86 (d, 1 H, *J* 8.4 Hz, H_Ph_), 8.13 (d, 1 H, *J* 7.8 Hz, H_Ph_), 8.26 (s, 1 H, H_Ph_), 11.99 (s, 1 H, NH_Indol_); ^13^C NMR (DMSO-*d*_6_, 150 MHz) *δ* 23.0, 103.9, 112.0, 119.8, 121.2, 122.4, 123.1, 123.4, 126.6, 127.6, 130.3, 131.4, 134.6, 135.8, 136.9, 142.0, 146.7, 154.8; HRMS (ESI^+^) calcd for C_18_H_13_BrN_5_S (M + 1): 410.00750. Found: 410.0069.

## 4. Conclusions

PTSA were found to be a useful catalyst for the synthesis of indolo-triazolo-thiadiazepine-targeted molecules in high yields. The obtained compounds were fully characterized, and the X-ray crystal structure for **4a** was resolved. The triazolo-thiadiazepine scaffold represents a very promising scaffold, being spider-like, and is suitable for the parallel synthesis and subsequent development of highly selective EGFR inhibitors, as well as for selective multi-targeting to improve efficacy and reduce toxicity. We successfully prepared compounds demonstrating good antiproliferative activity, compared to that of the standard drug doxorubicin, including compounds **4b**, and **4j**. The molecular modeling studies demonstrated that the scaffold acts as an anchor binding the key residue Met793 and allowing the substituents to interact with the substrate access channel and the hydrophobic sub pocket. This work represents a good starting point to utilize triazolo-thiadiazepine as a promising scaffold for the optimization and preclinical development of EGFR inhibitors. 

## Figures and Tables

**Table 1:** IC_50_ results for indolyl-triazolothiadiazepine hybrids tested on HEPG2 and MCF-7 cancer cell lines.

#	Inhibition % HEPG-2 (100 µg/mL)	IC_50_ (µg/mL) HEPG-2	Inhibition % MCF-7 (100 µg/mL)	IC_50_ (µg/mL) MCF-7
**4a**	68.6	37	69.3	47.8
**4b**	59.5	44.6	76.4	20.2
**4c**	50.0	NT	63.6	14.7
**4d**	53.6	44.6	80.7	49.3
**4e**	44.4	30.1	77.9	26.9
**4f**	74.5	38.7	73.6	43.2
**4g**	74.5	43.2	72.9	48.7
**4h**	16.3	NT	45.7	NT
**4i**	66.7	15.2	51.4	NT
**4j**	61.4	12.9	32.9	NT
**4k**	56.1	NT	53.6	NT
**8a**	NT	NT	40.0	NT
**8b**	28.1	NT	21.4	NT
**Doxorubicin**		**4.0**		**4.0**

NT = Not Tested.

## Supplementary Materials

NMR spectrum, X-ray crystallographic analysis, cytotoxicity assay, and molecular docking study associated with this article can be found in the online version.
