# Supplementary material for: Synthesis and Anti-Proliferative Assessment of Triazolo-Thiadiazepine and Triazolo-Thiadiazine Scaffolds
Source: Molecules. 2019 Dec 6;24(24):4471. doi: 10.3390/molecules24244471 (PMC6943432; doi:10.3390/molecules24244471)
Supplement: Supplementary file 1 [file molecules-24-04471-s001.pdf]

SUPPORTING INFORMATION

Synthesis and anti-proliferative assessment of triazolo-thiadiazepine and triazolo-thiadiazine scaffolds

Ahmed T. A. Boraei [1,*], Hazem A. Ghabbour [2], Mohamed S. Gomaa [3], El Sayed H. El Ashry[5] and Assem Barakat [4,5,*]

1 Chemistry Department, Faculty of Science, Suez Canal University, Ismailia 41522, Egypt.
2 Department of Medicinal Chemistry, Faculty of Pharmacy, Mansoura University, Mansoura 35516, Egypt; hghabbour@mans.edu.eg
3 Pharmaceutical Chemistry Department, College of Clinical Pharmacy, Imam Abdulrahman Bin Faisal University, Dammam 32241, Kingdom of Saudi Arabia; mohamdgomaa75@gmail.com
4 Chemistry Department, College of Science, King Saud University, P.O. Box 2455, Riyadh, 11451, Saudi Arabia.
5 Chemistry Department, Faculty of Science, Alexandria University, P.O. Box 426, Ibrahimia, Alexandria 21321, Egypt; eelashry60@hotmail.com
* Correspondence: ahmed_tawfeek83@yahoo.com (A.T.A.B); ambarakat@ksu.edu.sa (A.B.); Tel.: +966-11467-5901 (A.B.); Fax: +966-11467-5992 (A.B.).

X-ray data, cytoxicity assay and molecular docking protocol.

Copies of the spectra used for characterization of the products,

Table S1. Crystal structure and refinement data of compound **4a**.

**Crystal Data**	
Chemical formula	$C_{25}H_{19}N_5S$
M*r*	421.51
Crystal system, space group	Triclinic, *P*-1
Temperature (K)	100
a, *b*, *c* (Å)	10.1159 (11), 10.6507 (11), 10.9680 (12)
V (Å^3)	1018.99 (19)
Z	2
Radiation type	Mo K*α*
μ (mm^{-1})	0.18
Crystal size (mm)	0.34 × 0.27 × 0.06

Data Collection	
Diffractometer	Bruker APEX-II D8 venture diffractometer
Absorption correction	Multi-scan, SADABS Bruker 2014
T_{min}, T_{max}	0.940, 0.990
No. of measured, independent and observed [$I > 2\sigma(I)$] reflections	18033, 4665, 2884
R_{int}	0.136
**Refinement**	
$R[F^2 > 2\sigma(F^2)]$, $wR(F^2)$, S	0.065, 0.152, 1.03
No. of reflections	4665
No. of parameters	284
No. of restraints	0
H-atom treatment	H atoms treated by a mixture of independent and constrained refinement
$\Delta\rho_{max}$, $\Delta\rho_{min}$ (e Å^{-3})	0.47, − 0.49
CCDC No.	1465170

Table S2. Selected bond lengths and bond angles in compound **4a.**

Atoms	Å, º	Atoms	Å, º
S1—C10	1.738 (3)	N3—C10	1.303 (4)
S1—C11	1.853 (3)	N4—N5	1.393 (3)
N1—C1	1.368 (4)	N4—C9	1.371 (4)
N1—C8	1.378 (4)	N4—C10	1.369 (4)
N2—N3	1.394 (4)	N5—C13	1.296 (5)
N2—C9	1.318 (4)		
C10—S1—C11	104.62 (14)	N1—C8—C9	118.8 (3)

C1—N1—C8	109.0 (2)	N4—C9—C8	125.5 (3)
N3—N2—C9	107.8 (2)	N2—C9—C8	125.0 (3)
N2—N3—C10	107.2 (2)	N2—C9—N4	109.1 (3)
N5—N4—C9	123.0 (3)	S1—C10—N3	122.9 (2)
N5—N4—C10	131.2 (3)	S1—C10—N4	126.0 (2)
C9—N4—C10	105.5 (2)	N3—C10—N4	110.4 (3)
N4—N5—C13	115.8 (3)	S1—C11—C12	110.9 (2)
N1—C1—C6	107.6 (3)	S1—C11—C20	109.9 (2)
N1—C1—C2	130.0 (3)	N5—C13—C12	124.1 (3)
N1—C8—C7	109.2 (3)	N5—C13—C14	115.3 (3)

Table S3. Hydrogen-bond geometry (Å, °) in compound **4a**.

D*—H···*A	***D*—H**	**H···*A***	***D*···*A***	***D*—H···*A***
N1—H1N1···N2[i]	0.78 (3)	2.25 (3)	2.997 (3)	160 (3)
Symmetry code: (i) −x, −y, −z+1.				

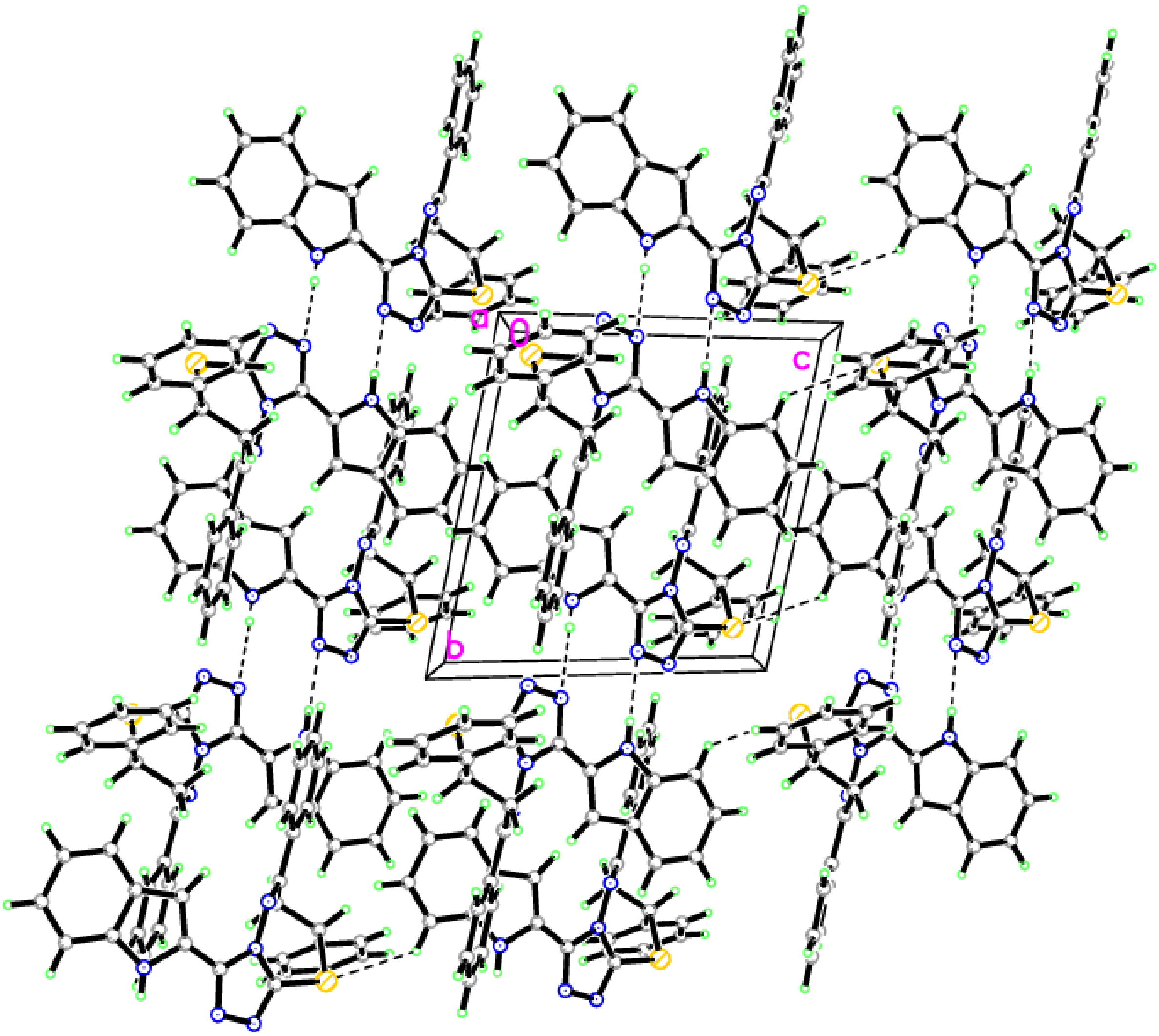

Figure S1. A view of the crystal packing down the *b* and *c* axes for compound **4a**. Dotted lines indicates the intermolecular interaction.

Experimental of Biological Assay

The antiproliferative assay was performed at the National Cancer Institute, Cairo, Egypt. The potential cytotoxicity of the compounds was tested using the method described by Skehan *et al*. [1]. Cells were plated in a 96-multiwell plate (104 cells/well) for 24 h before

treatment with the compounds to allow attachment of the cell to the wall of the plate. Different concentrations of the compound (0, 12.5, 25, 50, and 100 μg/mL) were added to the cell monolayer; triplicate wells were prepared for each individual dose. Monolayer cells were incubated with the compounds for 48 h at 37 °C and 5% CO_2. After 48 h, cells were fixed, washed, and stained with Sulfo-Rhodamine-B stain. Excess stain was washed with acetic acid and attached stain was recovered with TrisEDTA buffer. Color intensity was measured in an ELISA reader.

The relation between the surviving fraction and compound concentration was plotted to obtain a survival curve for the tumor cell lines for the specified compounds.

Molecular modeling

All molecular modeling studies were performed on a Hewlett-Packard Pentium Dual-Core T4300 2.10 GHz, running Windows 10 Ultimate using autodock 4.3 for molecular docking simulation and ligand binding energy calculation and Molsoft ICM-Pro 3.5-0 for output data visualization. The crystalline structure of human EGFR (PDB code; 1xkk) co-crystallized with inhibitor was used as the receptor in the docking studies. The selected target was used after deleting the co-crystallized inhibitor. Docking calculations were carried out using the AutoDock 4.3 software (La Jolla, CA) [40 in paper 1]. First, all hydrogens were added to the ligand PDB file, Gasteiger charges were computed, and all torsion angles of the ligands were defined using the autodock-tools program, to be

explored during molecular modeling. A grid box of 25 × 25 × 25Å with a grid spacing of 0.375Å and centered at the crystallized ligand was used to calculate the atom types needed for the calculation. The Lamarckian genetic algorithm was used as a search method with a total of 30 runs (maximum of 20 000 000energy evaluations; 27 000 generations; initial populations of 150conformers). The docking results were evaluated using the binding energy and cluster size calculation in autodock and by checking ligand binding position visually in Pymol.

Reference

1. Skehan, P.;, Storeng, R.; Scudiero, D.; Monks, A.; McMahon, J.; Vistica, D.; Warren, J.T.; Bokesch, H.; Kenney, S.; Boyd, M.R. New colorimetric cytotoxicity assay for anticancer-drug screening. *J. Natl. Cancer Inst.* **1990**, *82*, 1107–1112.

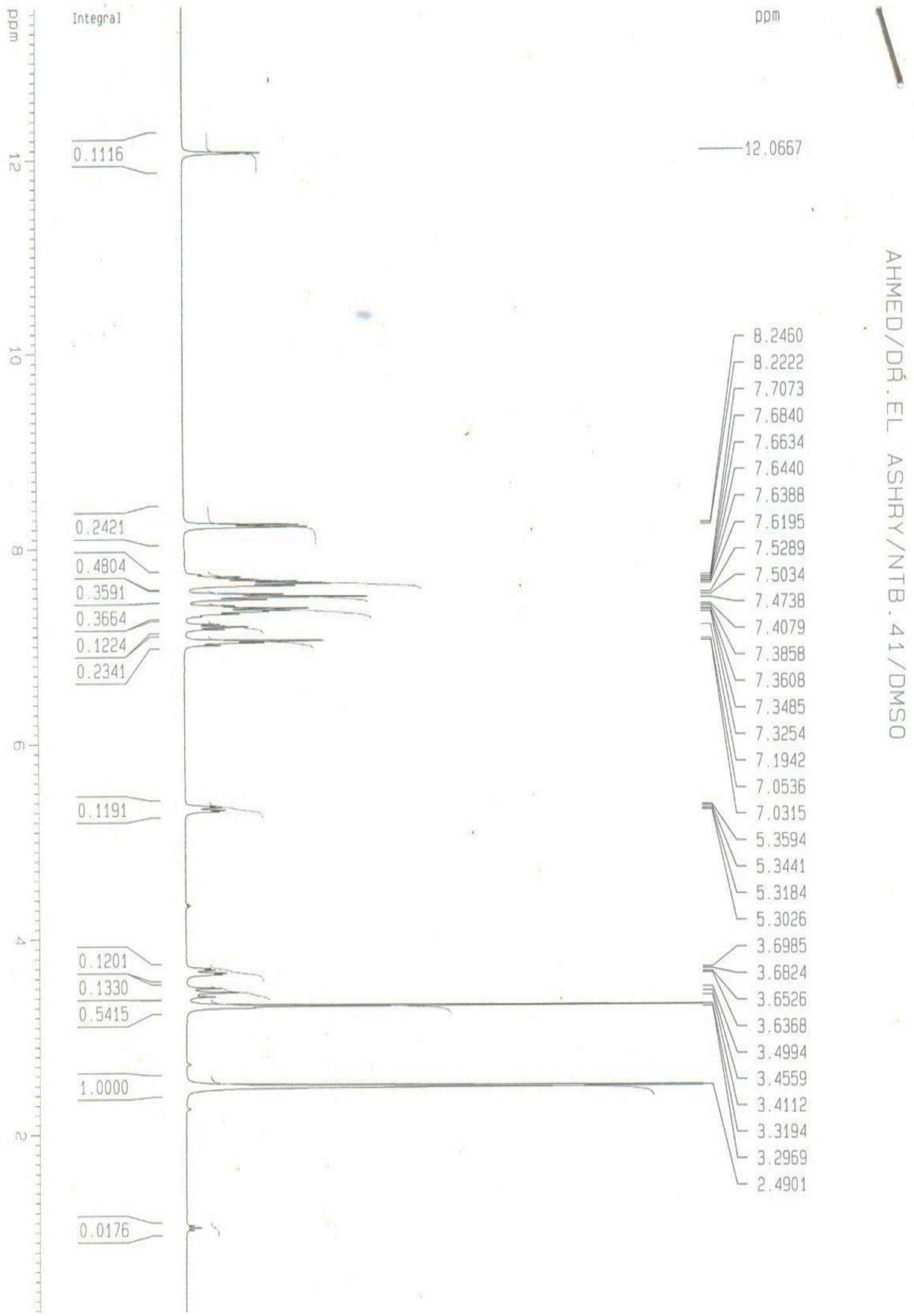

Figure S1. ^{1}H NMR of **4a**

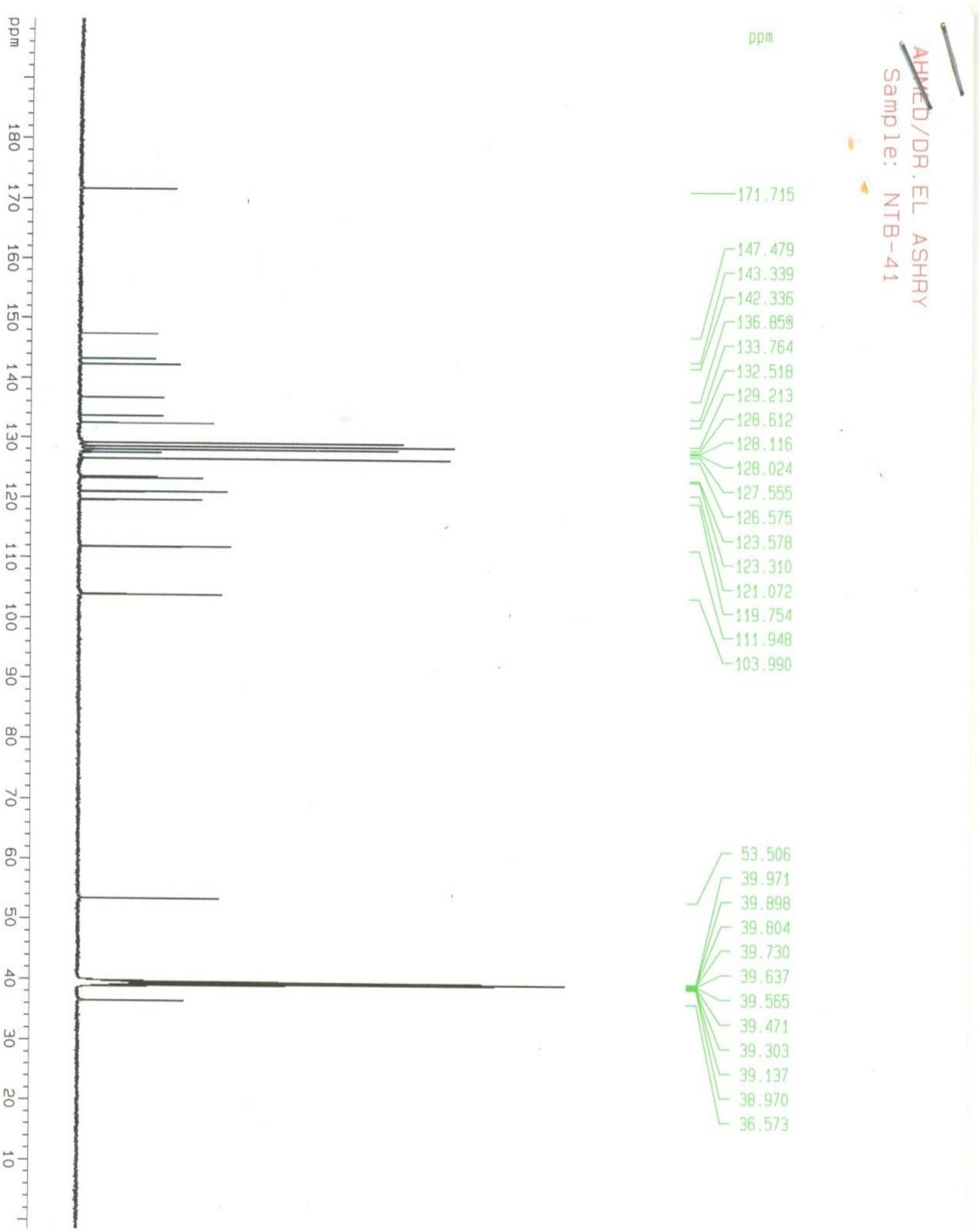

Figure S2. ^{13}C NMR of **4a**

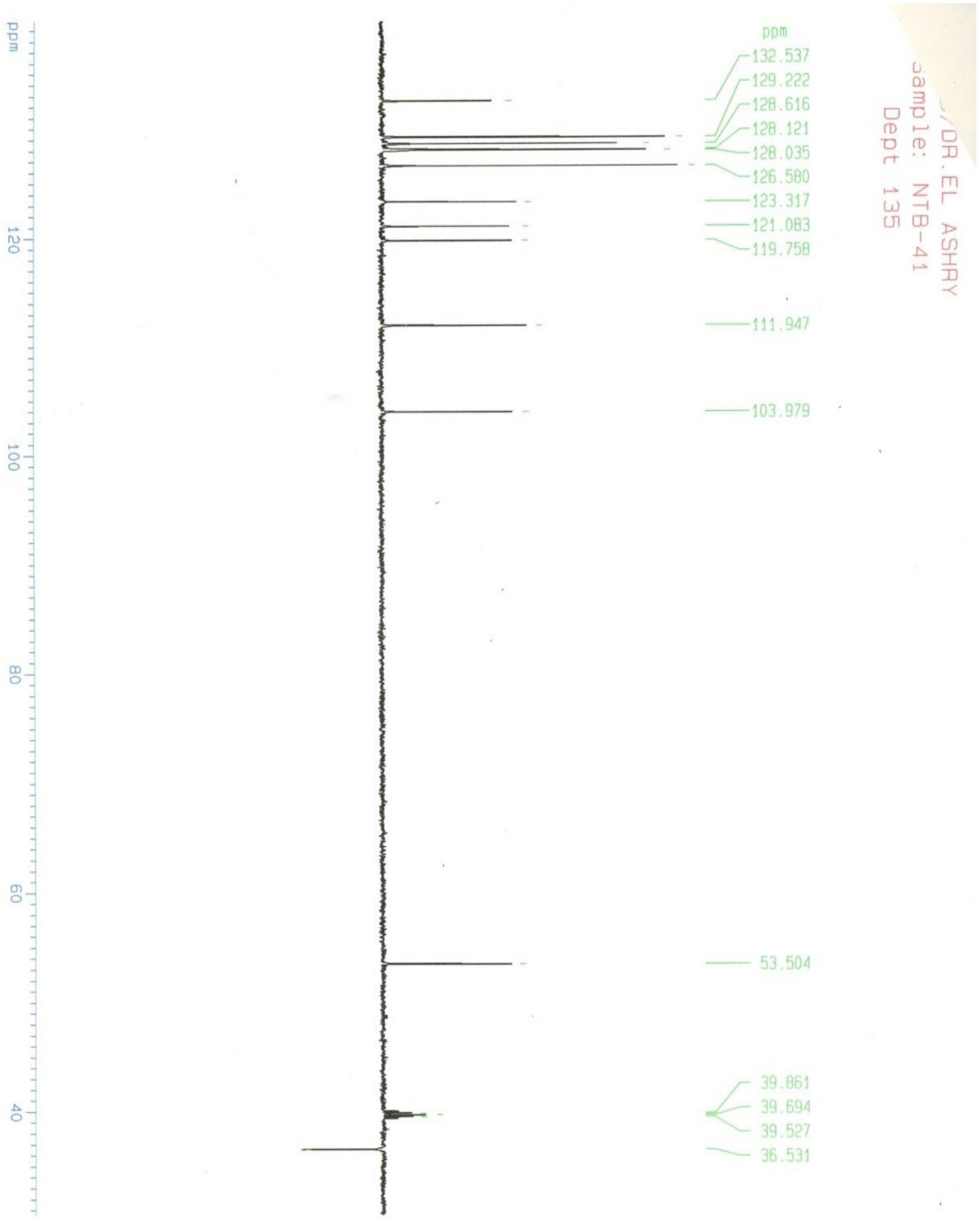

Figure S3. DEPT135 of **4a**

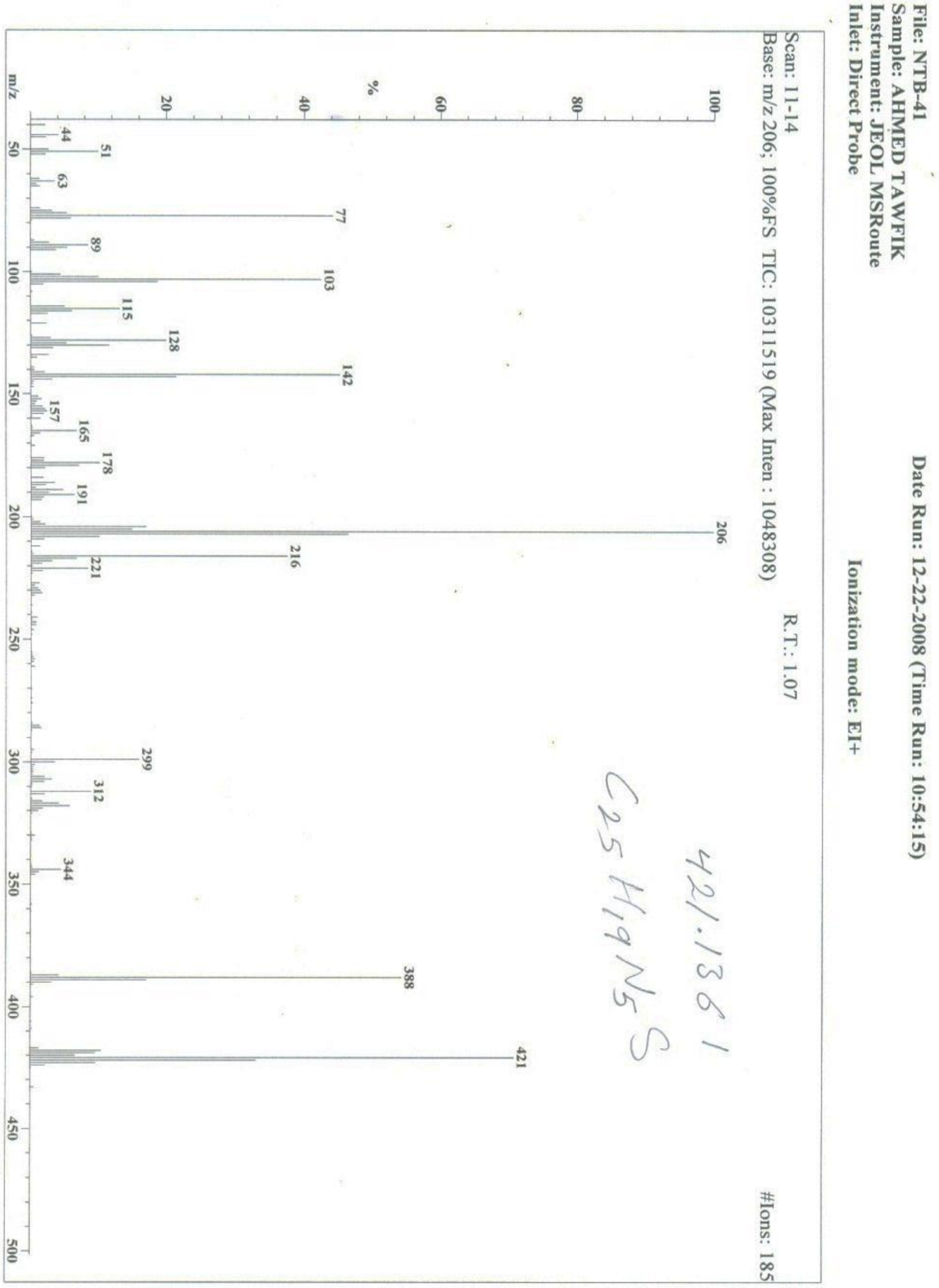

Figure S4. EIMS of **4a**

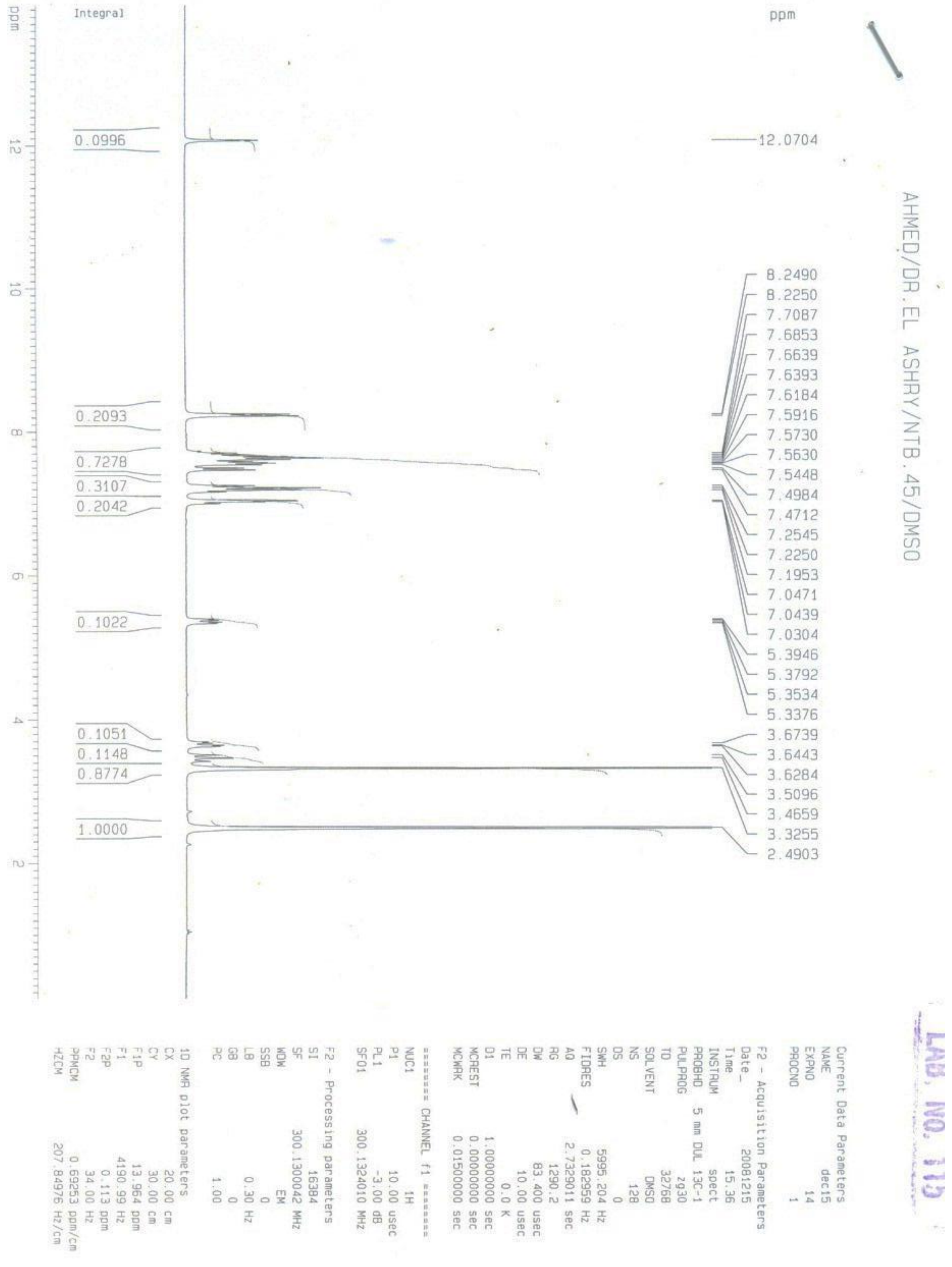

Figure S5. ^{1}H NMR of **4b**

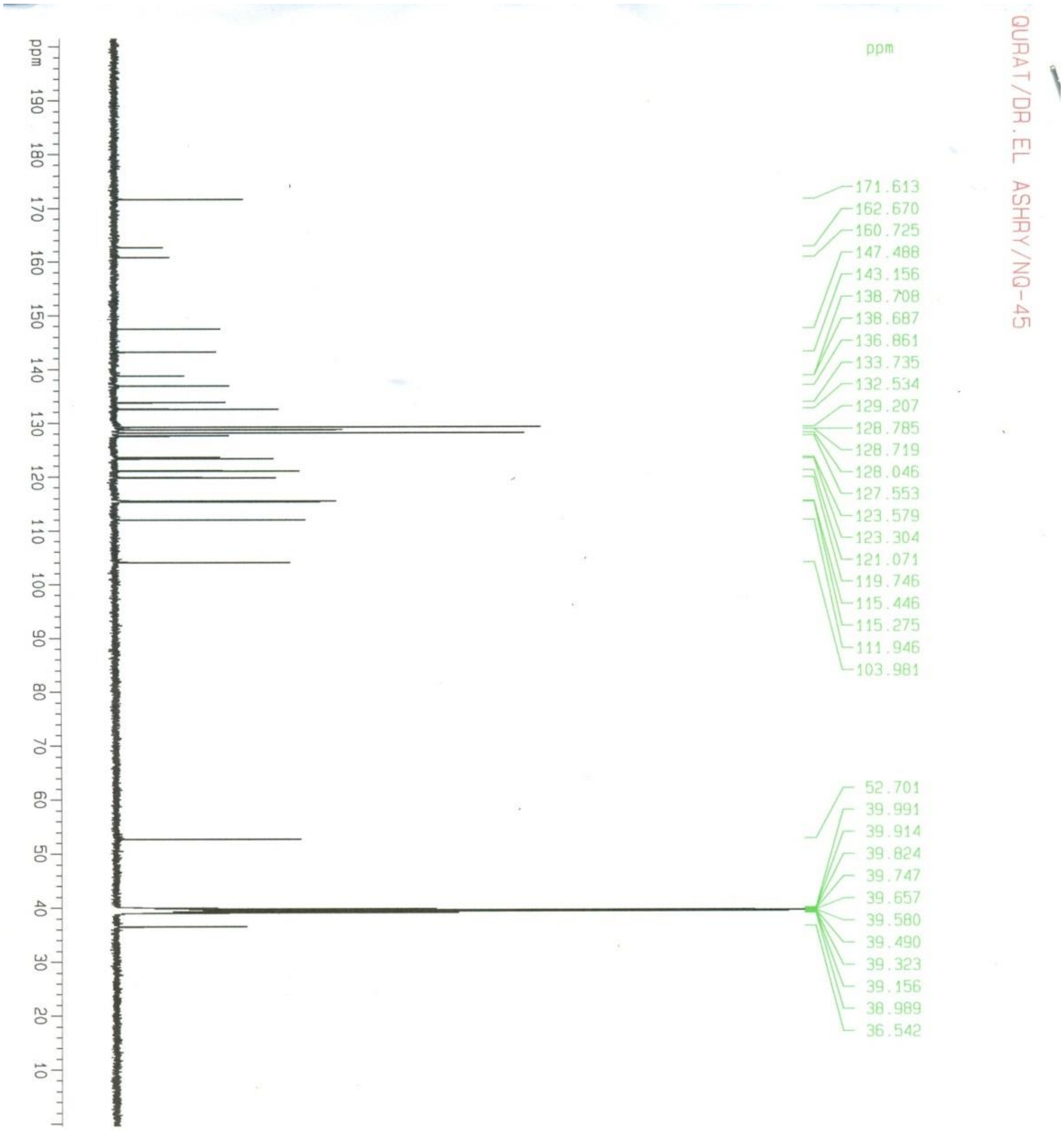

Figure S6. ^{13}C NMR of **4b**

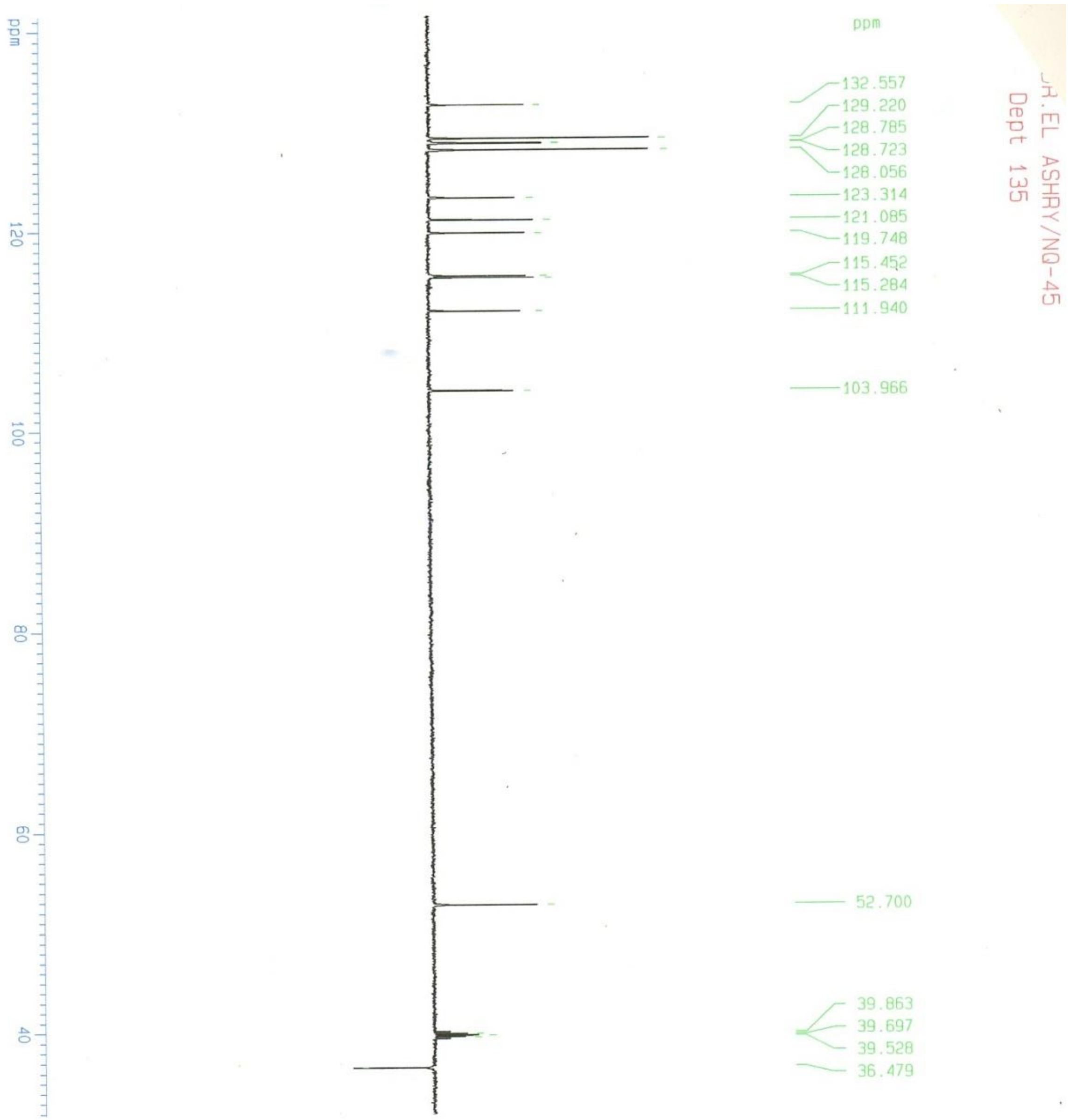

Figure S7. DEPT135 of **4b**

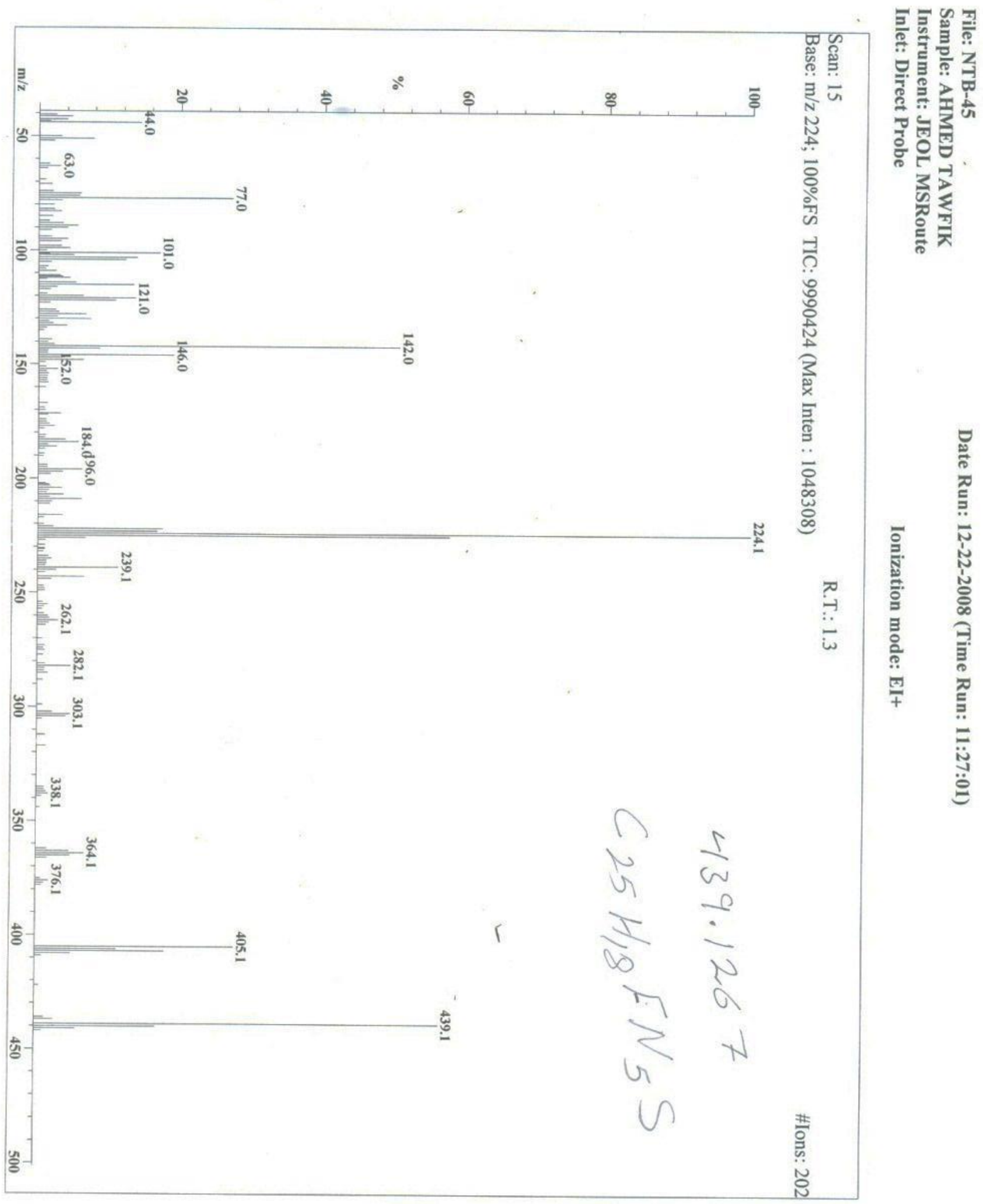

Figure S8. EIMS of **4b**

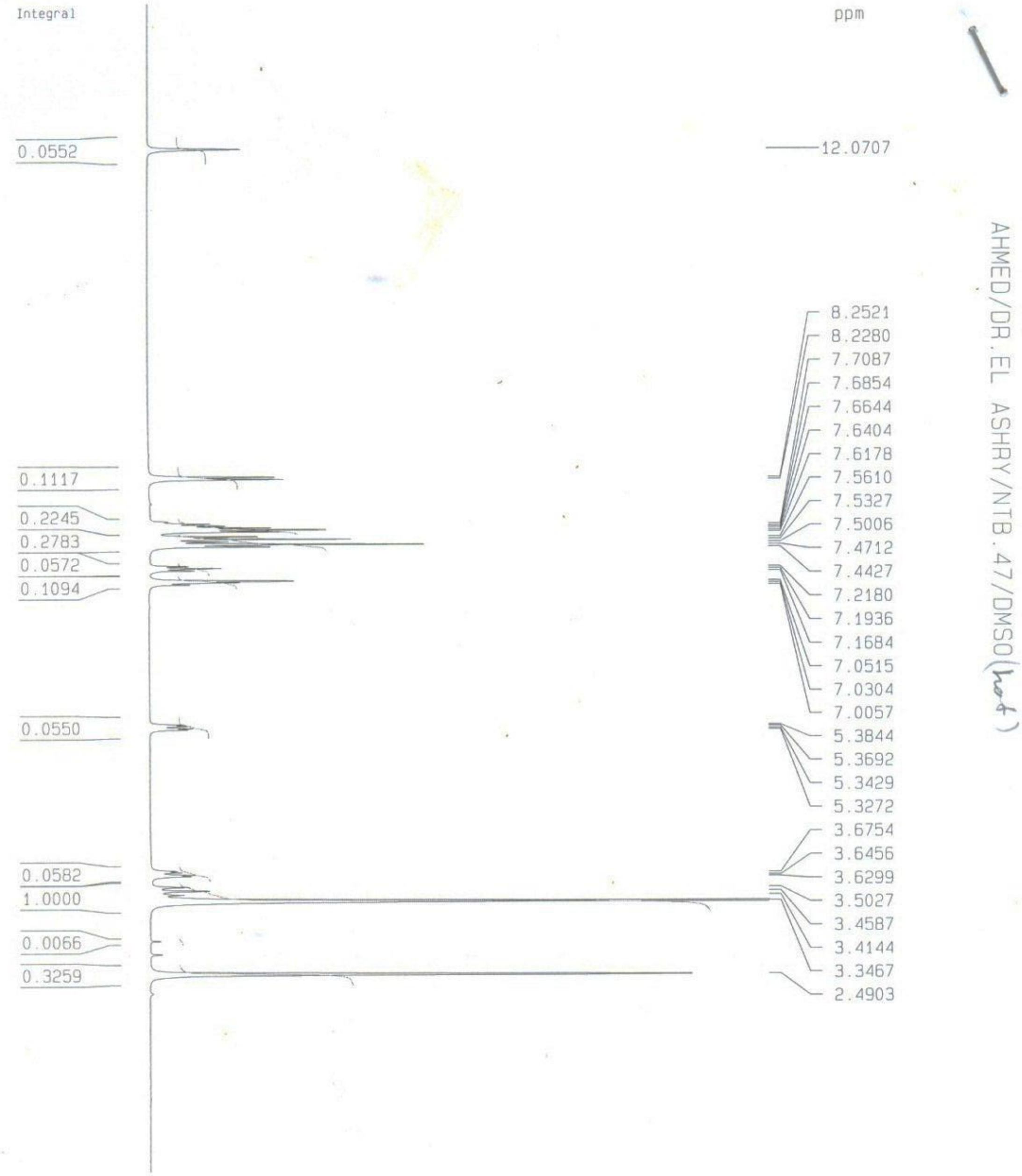

Figure S9. ^{1}H NMR of **4c**

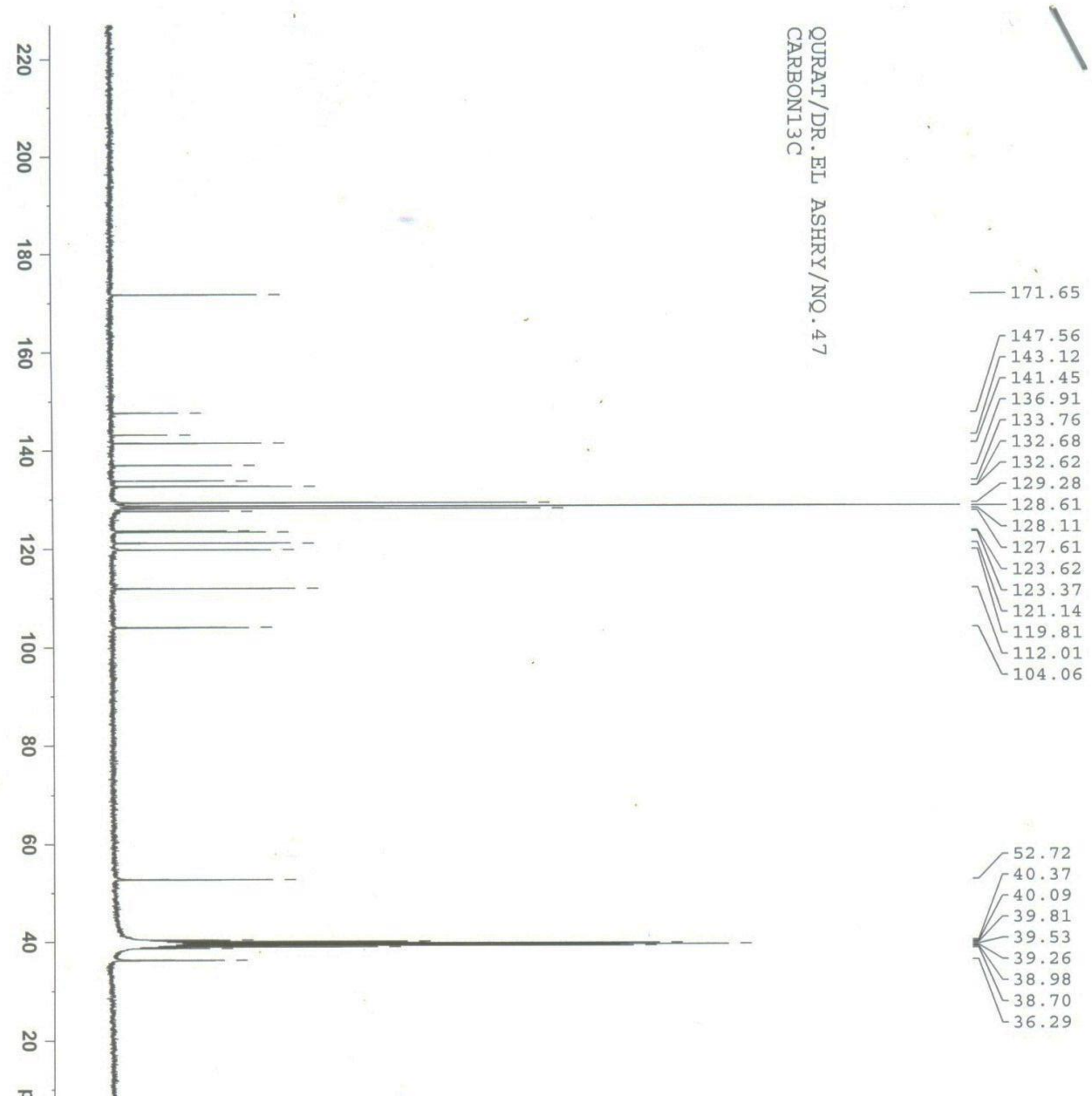

Figure S10. ^{13}C NMR of **4c**

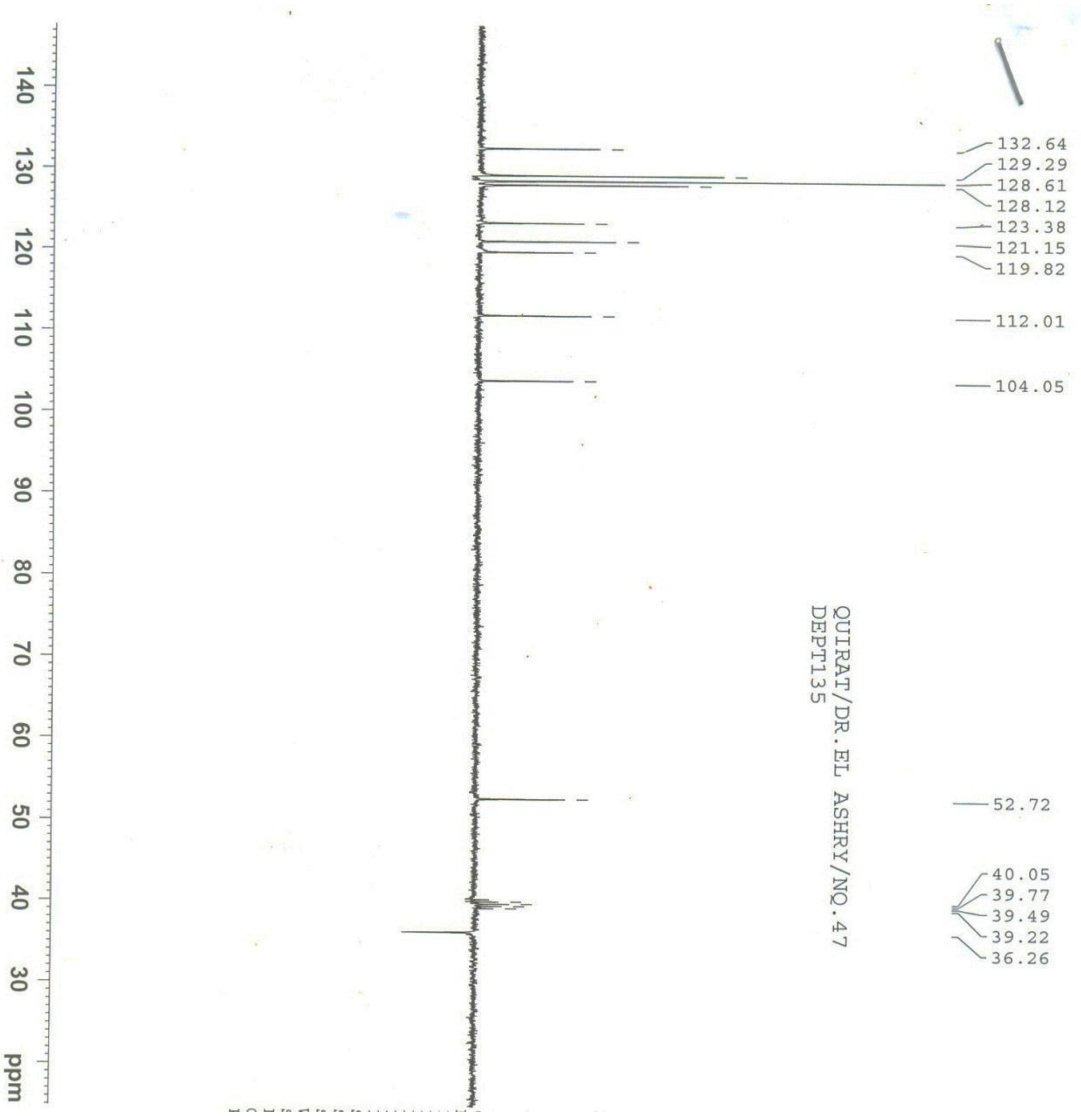

Figure S11. DEPT135 of **4c**

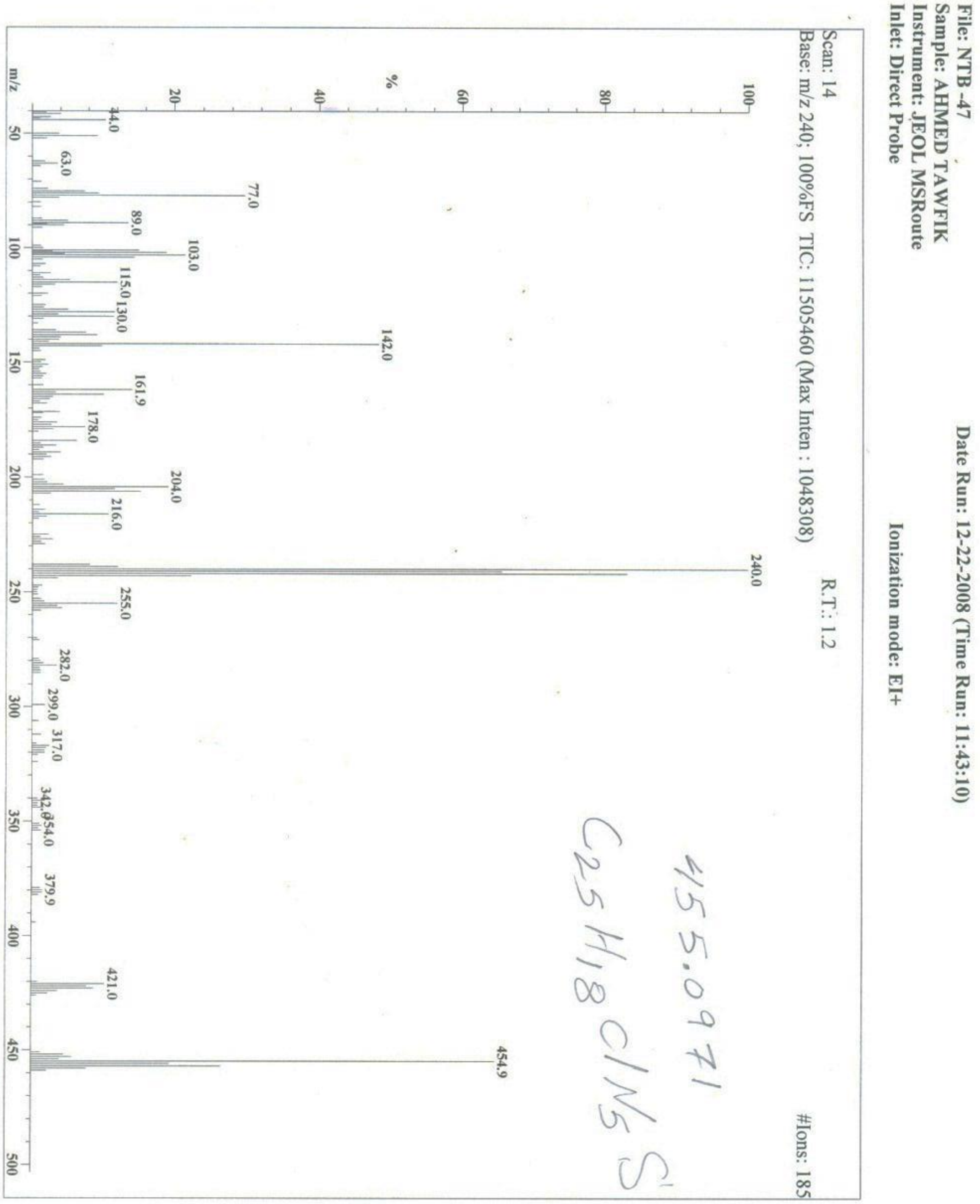

Figure S12. EIMS of **4c**

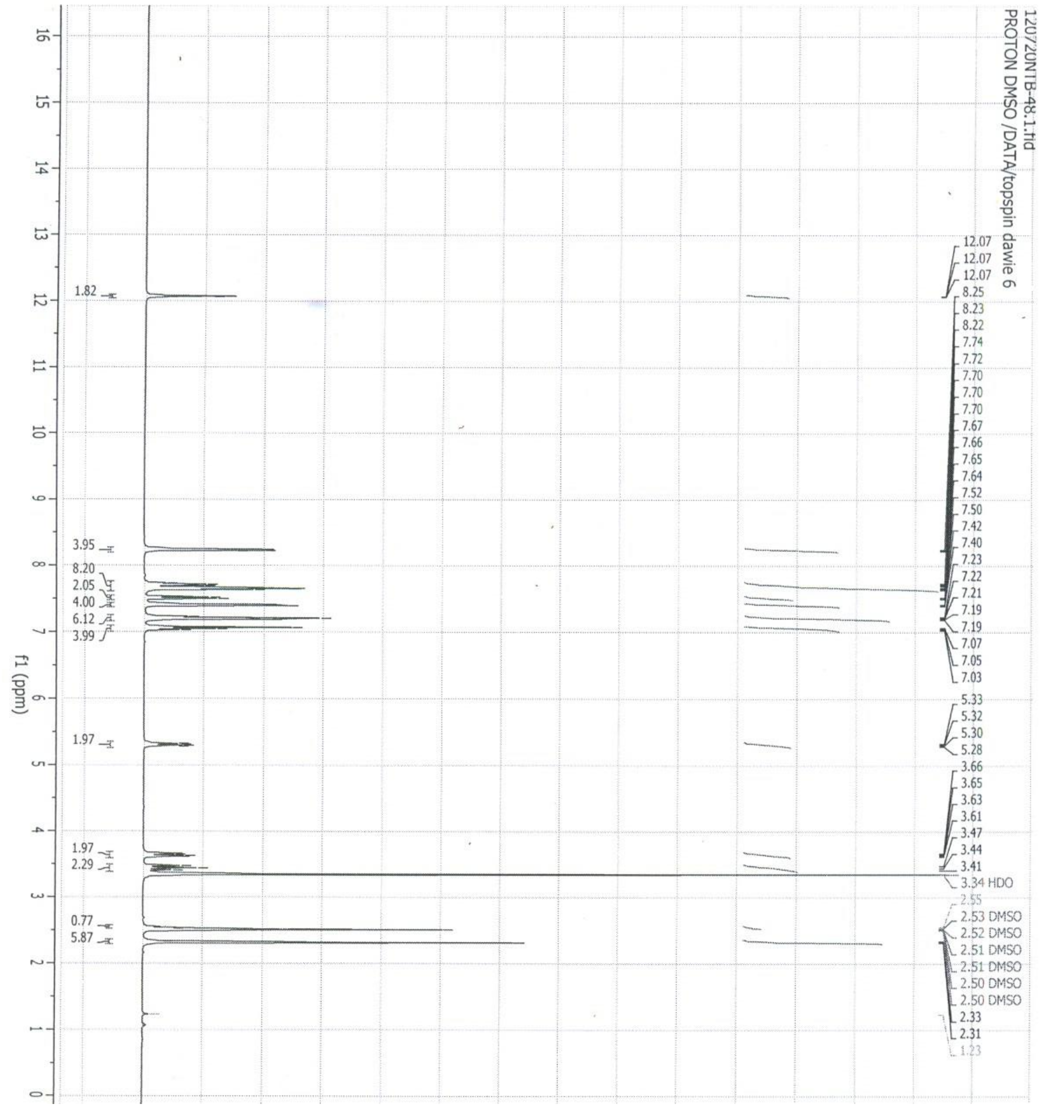

Figure S13. ^{1}H NMR of **4d**

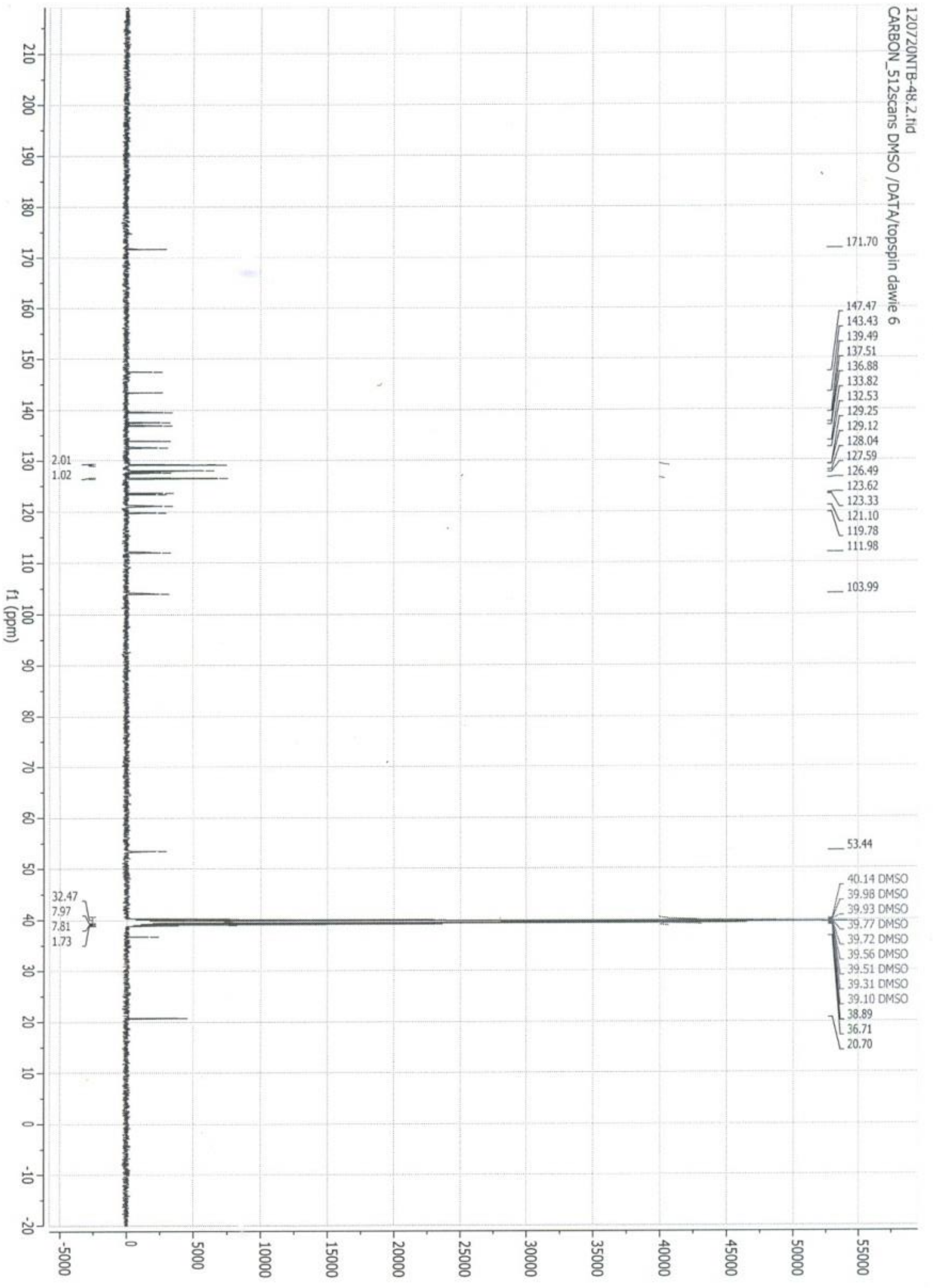

Figure S14. ^{13}C NMR of **4d**

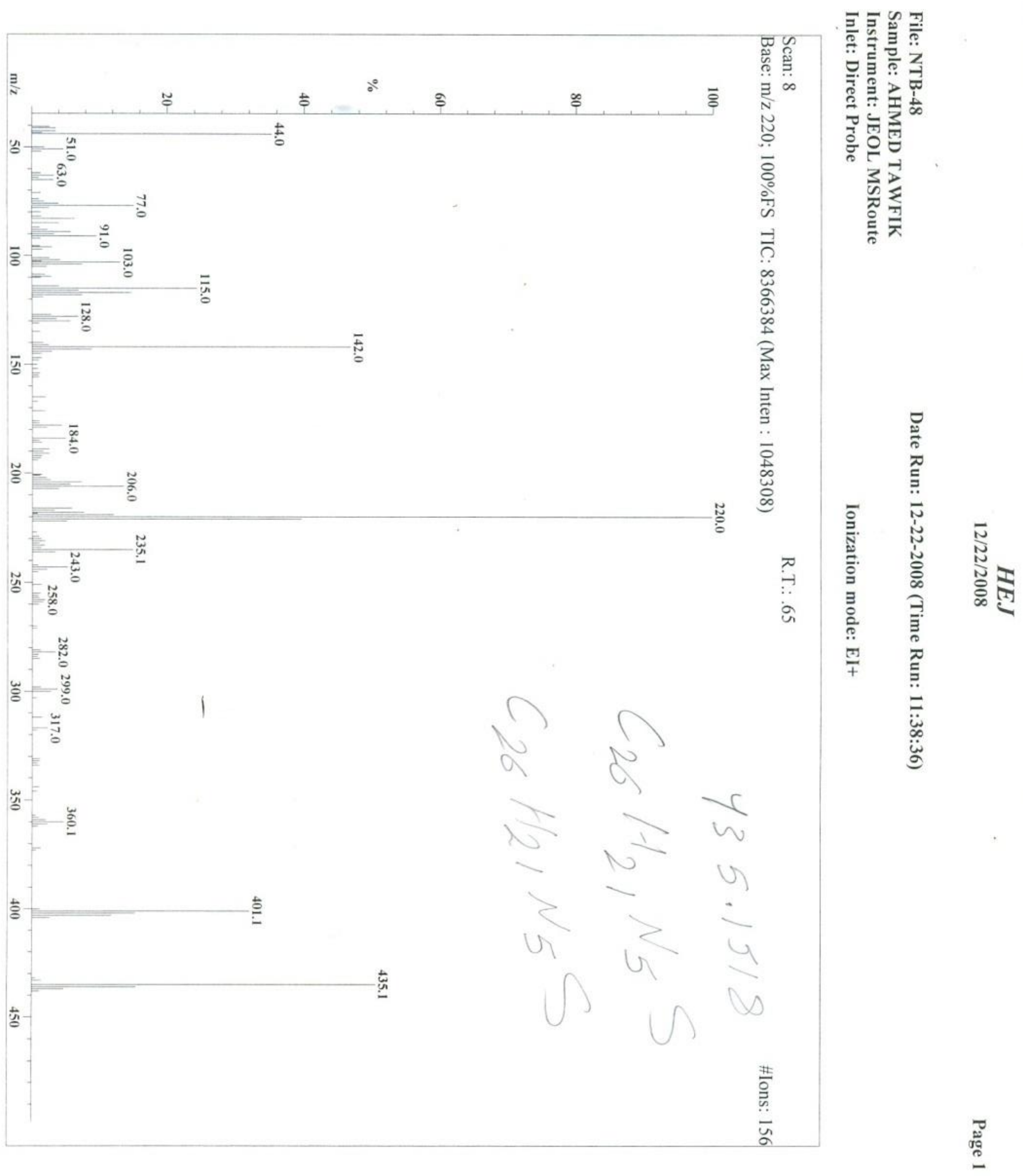

Figure S15. EIMS of **4d**

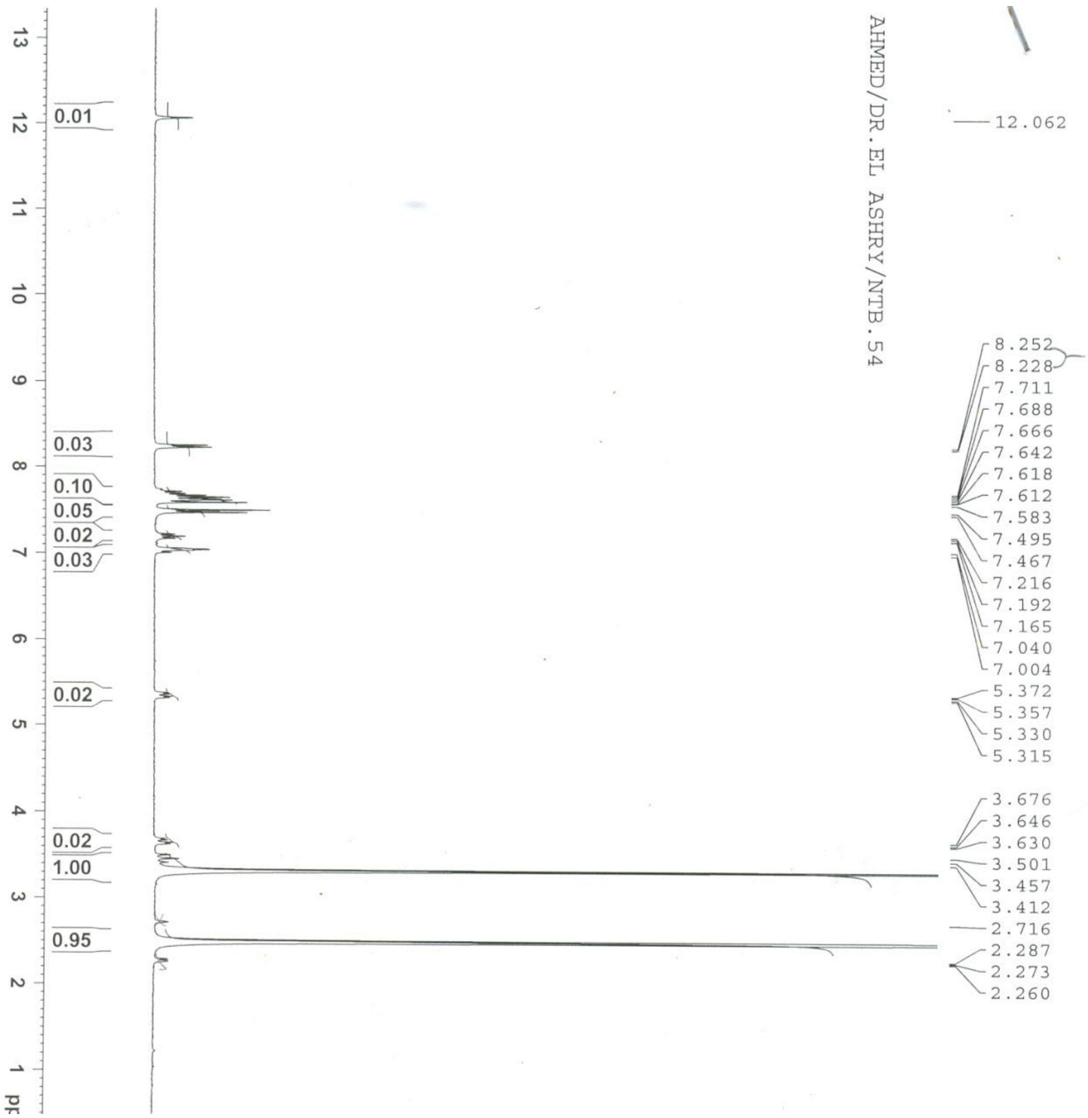

Figure S16. ^{1}H NMR of **4e**

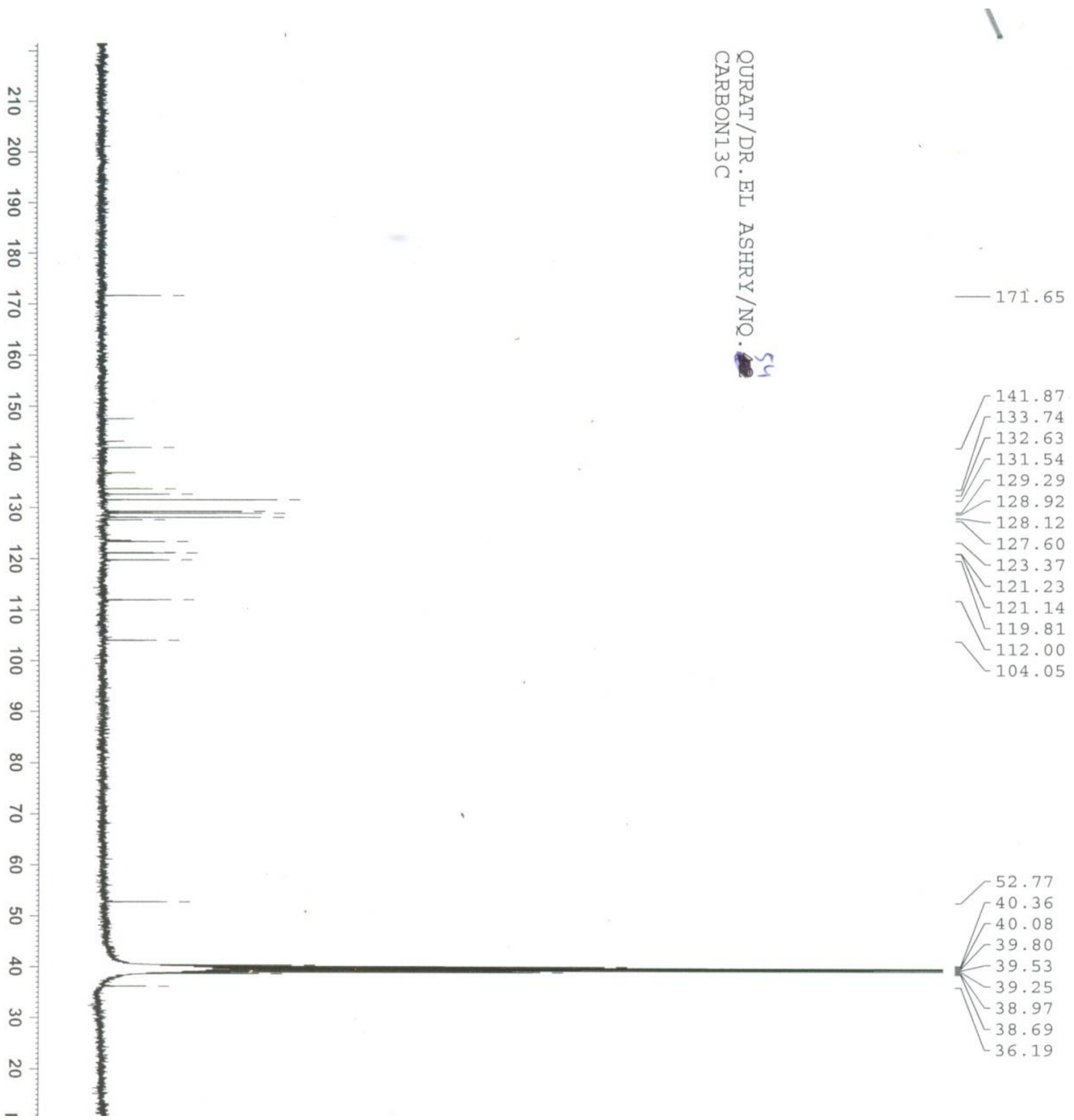

Figure S17. ^{13}C NMR of **4e**

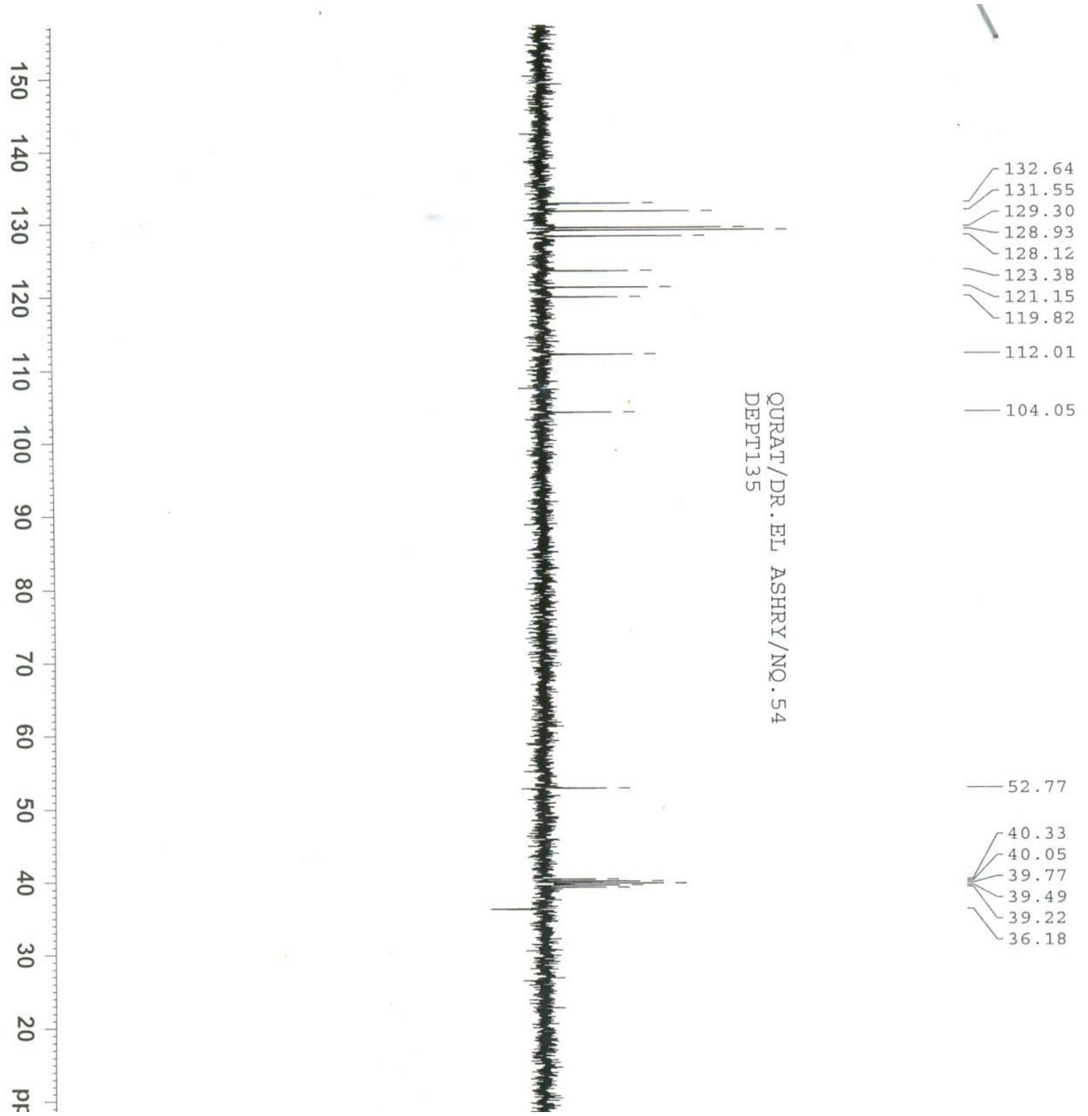

Figure S18. DEPT of **4e**

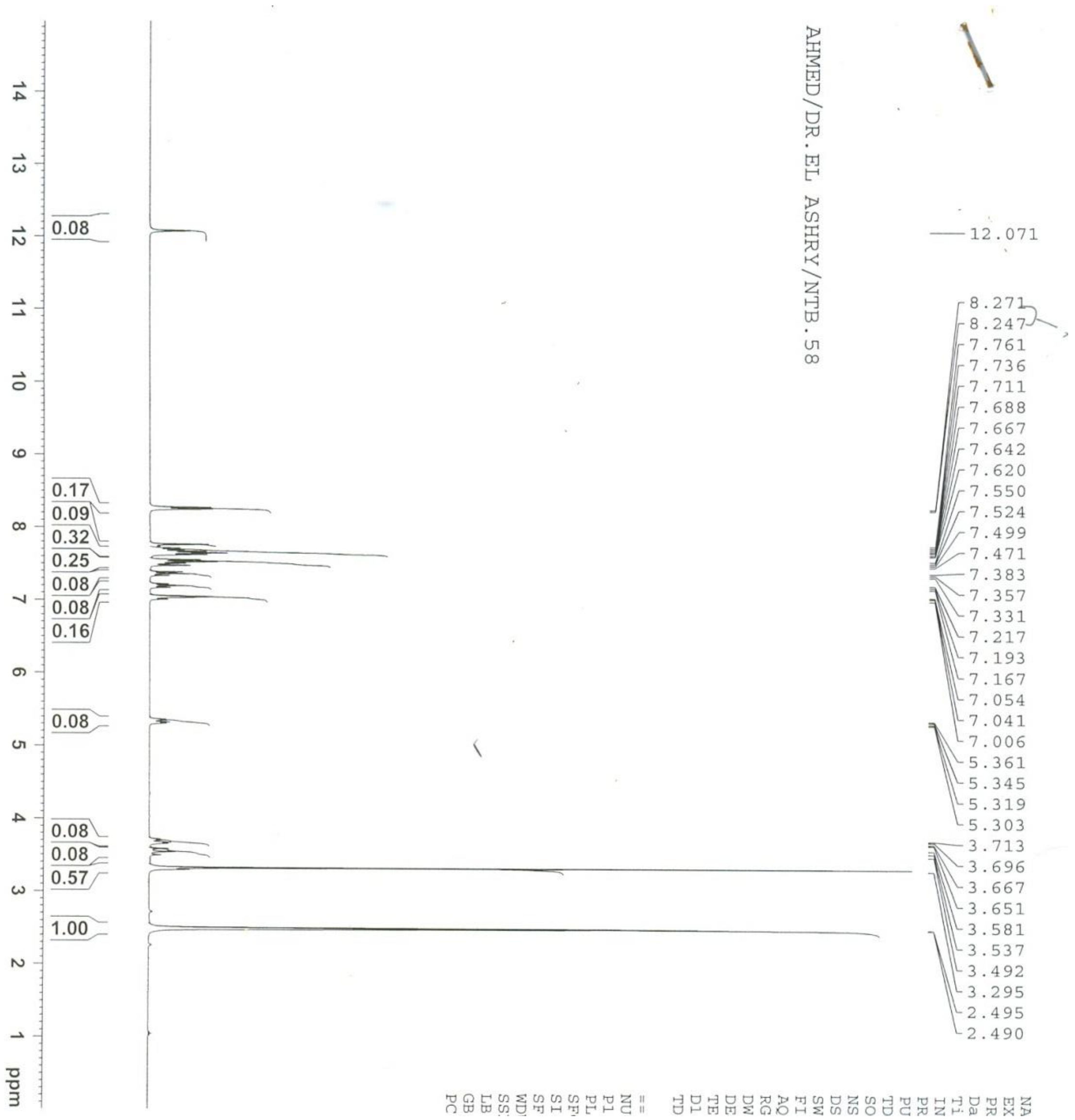

Figure S19. ^{1}H NMR of **4f**

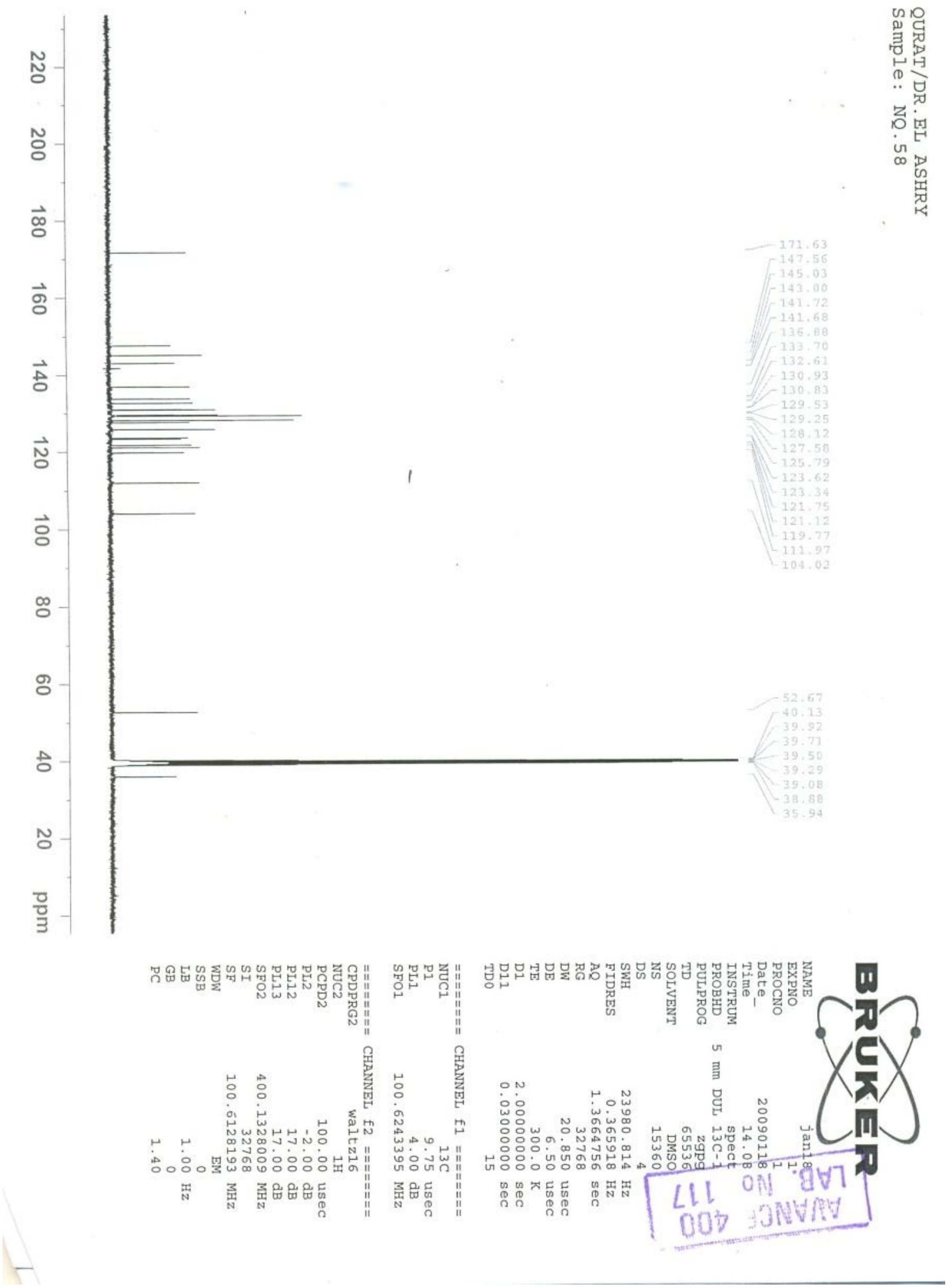

Figure S20. ^{13}C NMR of **4f**

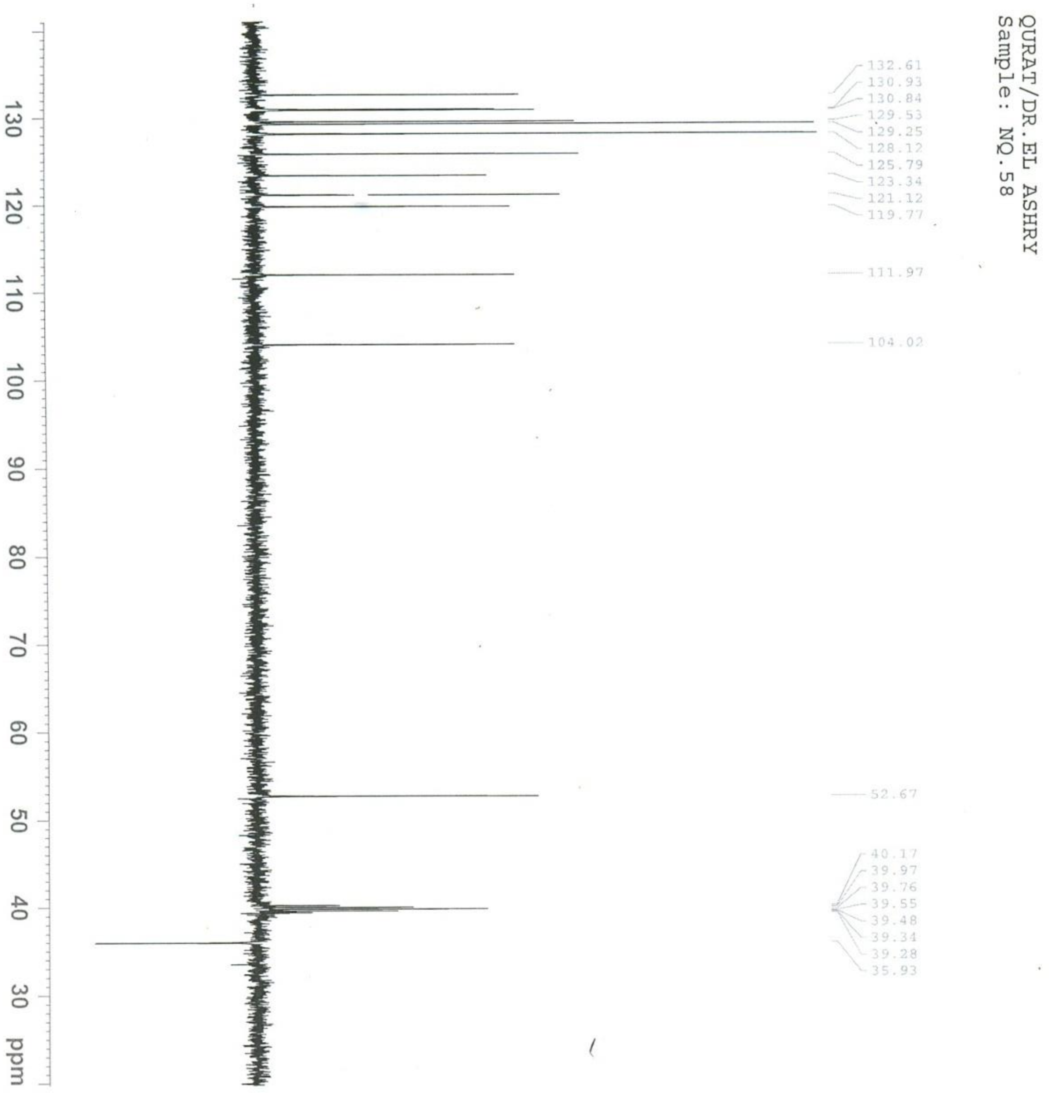

Figure S21. DEPT135 of **4f**

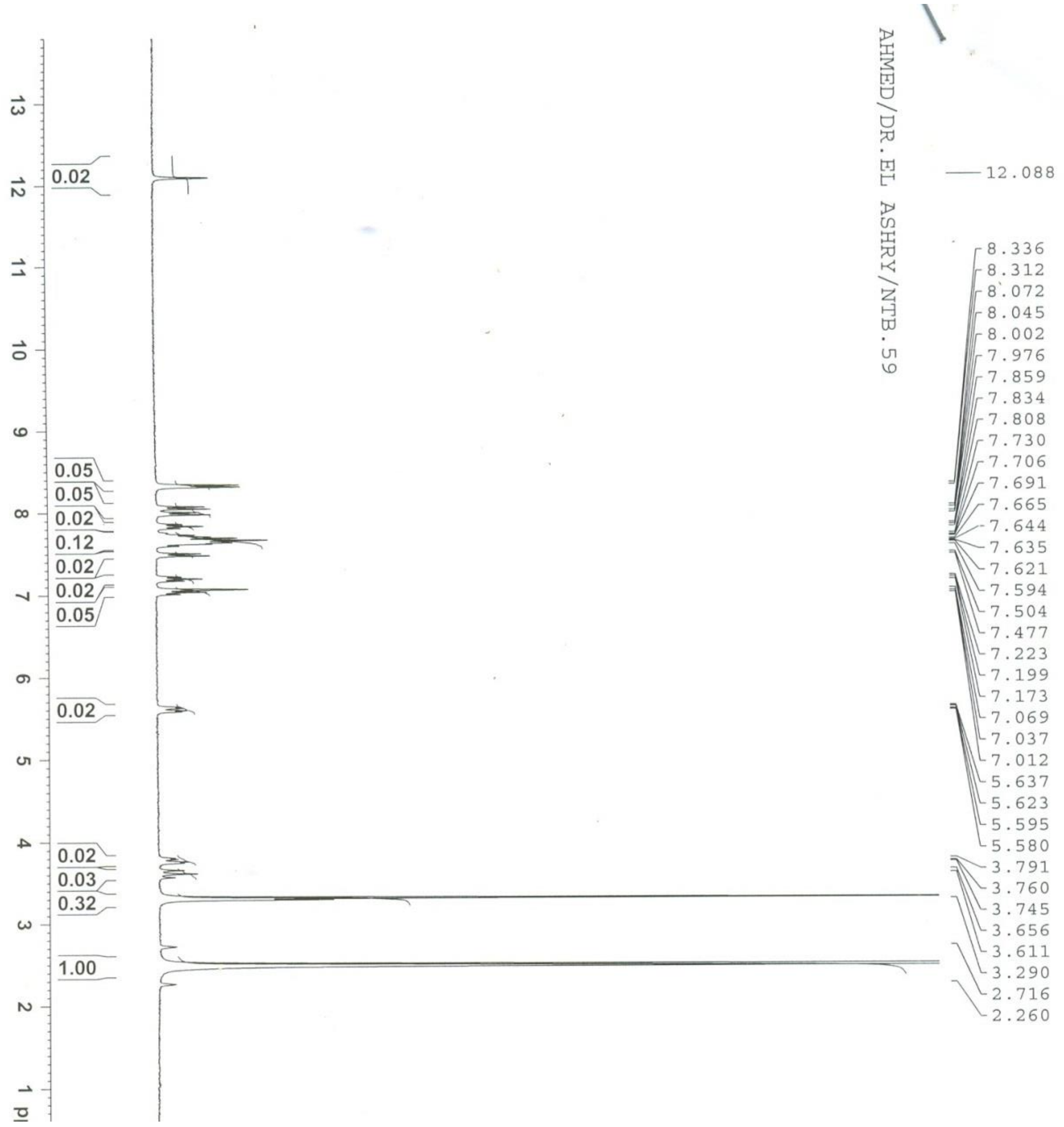

Figure S22. ^{1}H NMR of **4g**

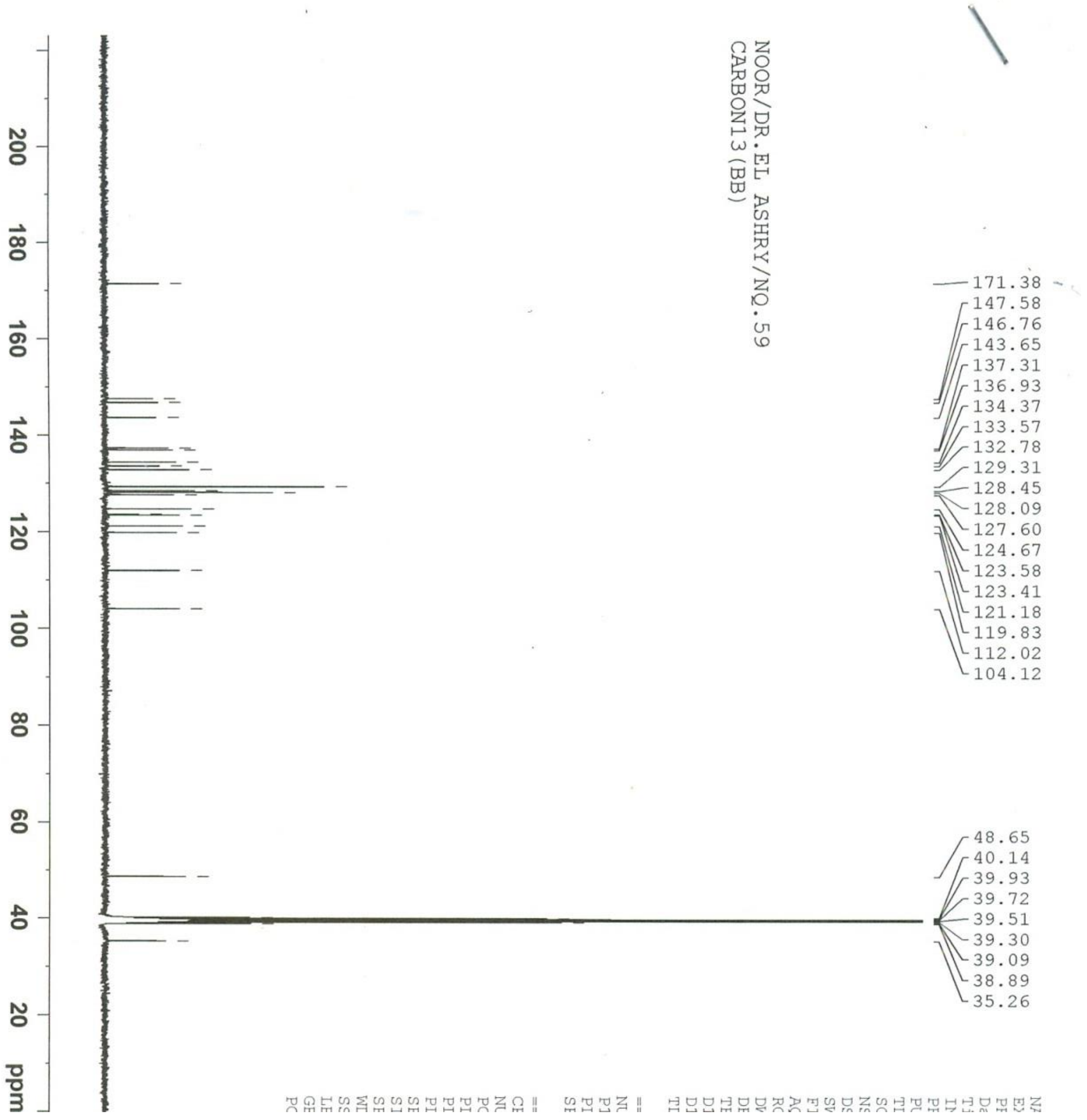

Figure S23. ^{13}C NMR of **4g**

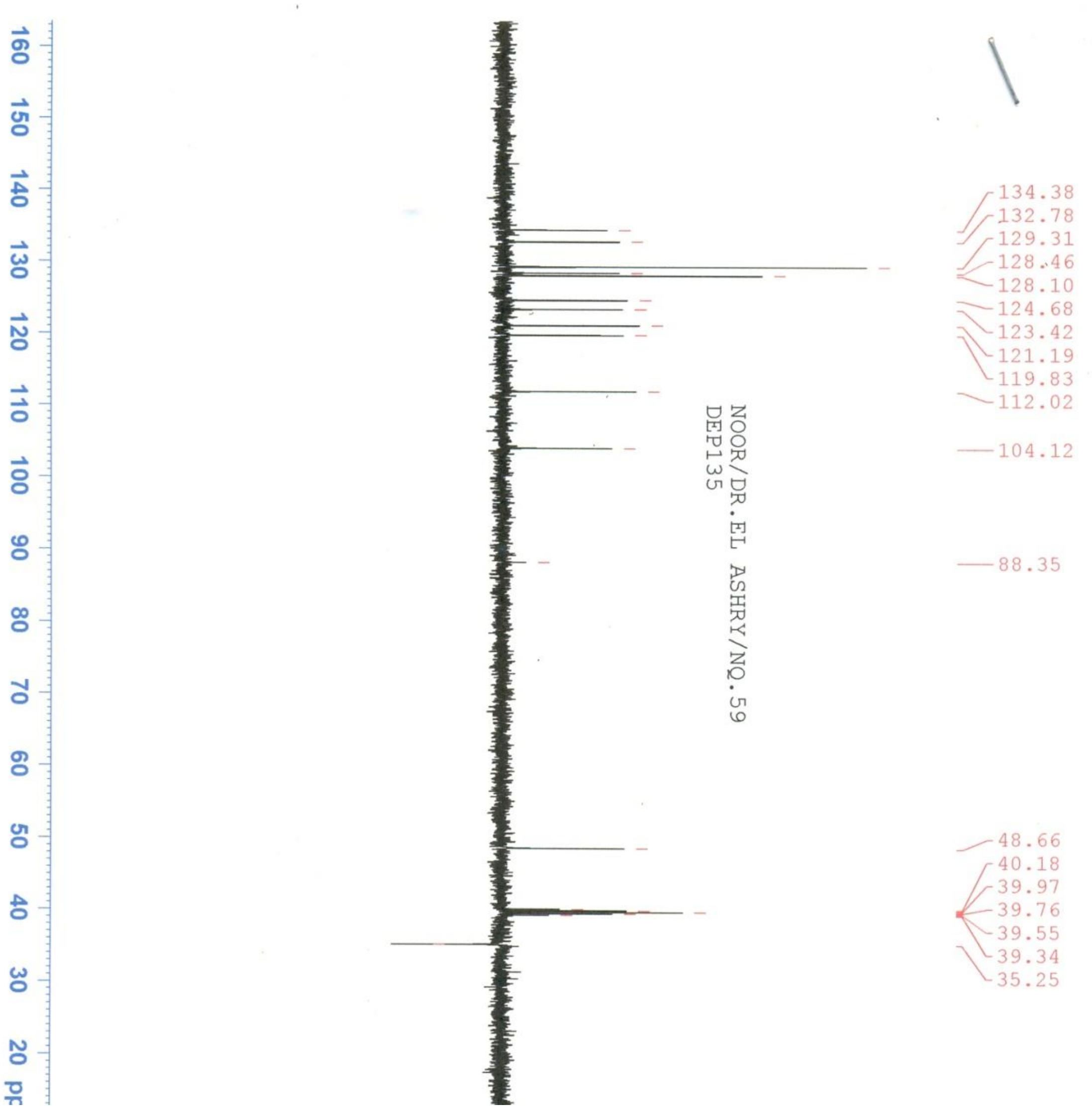

Figure S24. DEPT135 of **4g**

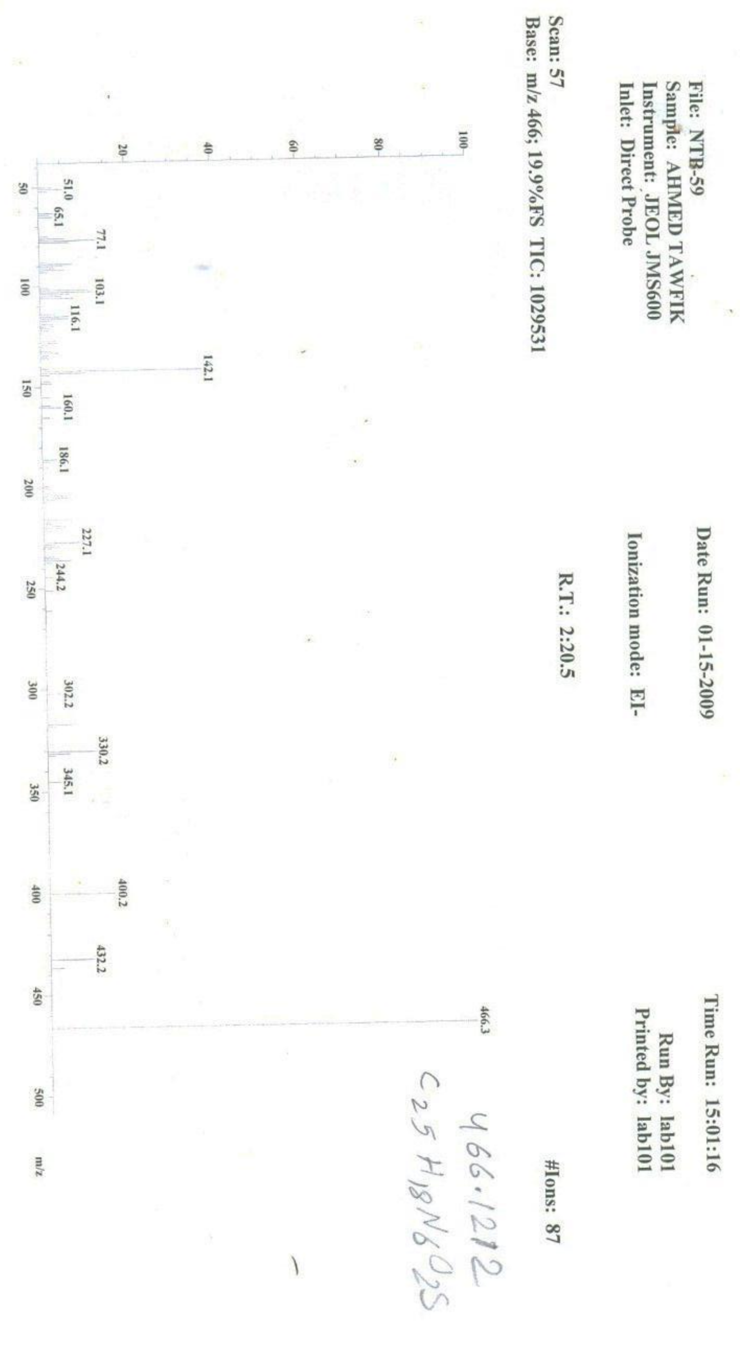

Figure S25. EIMS of **4g**

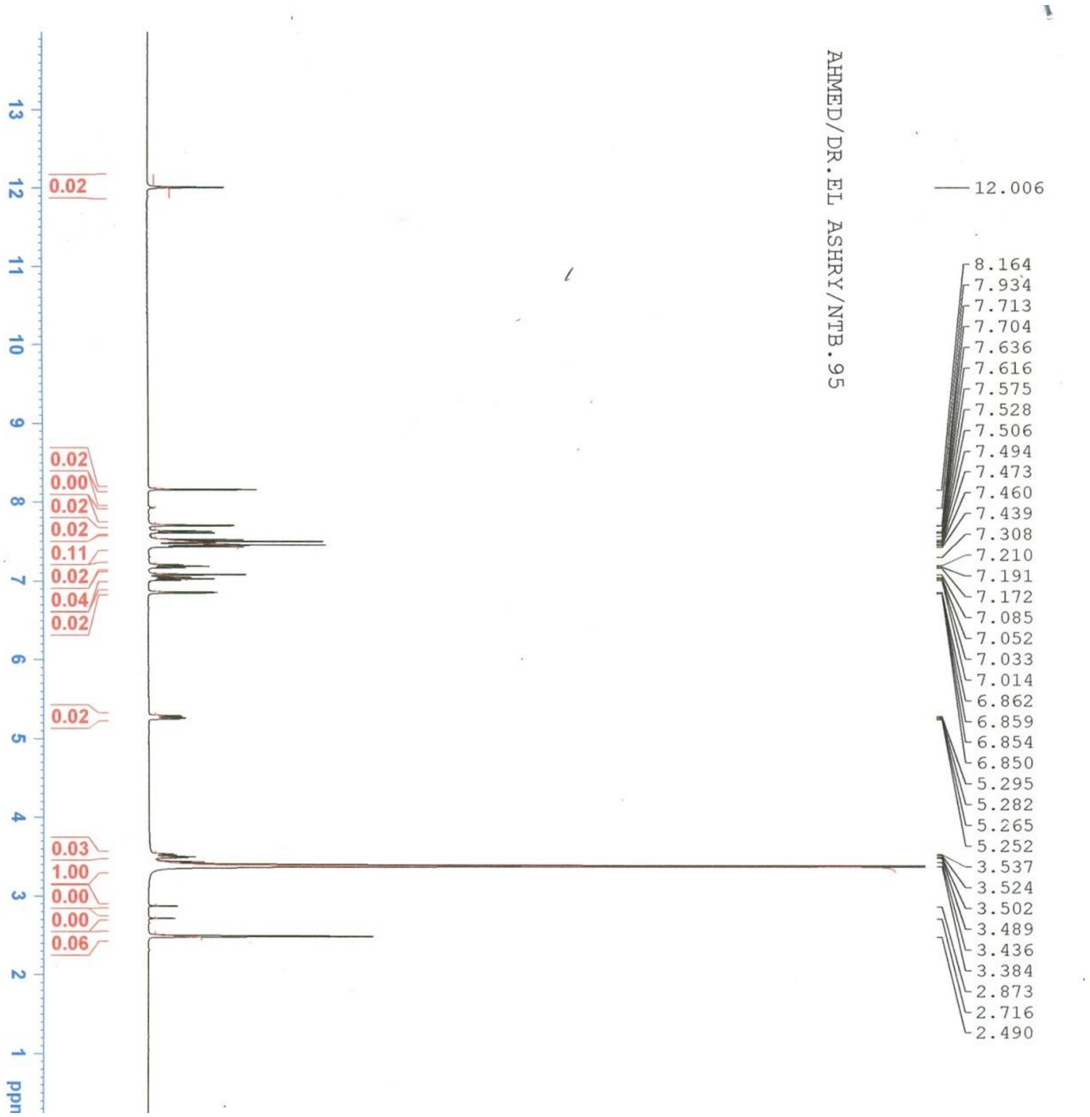

Figure S26. ^{1}H NMR of **4h**

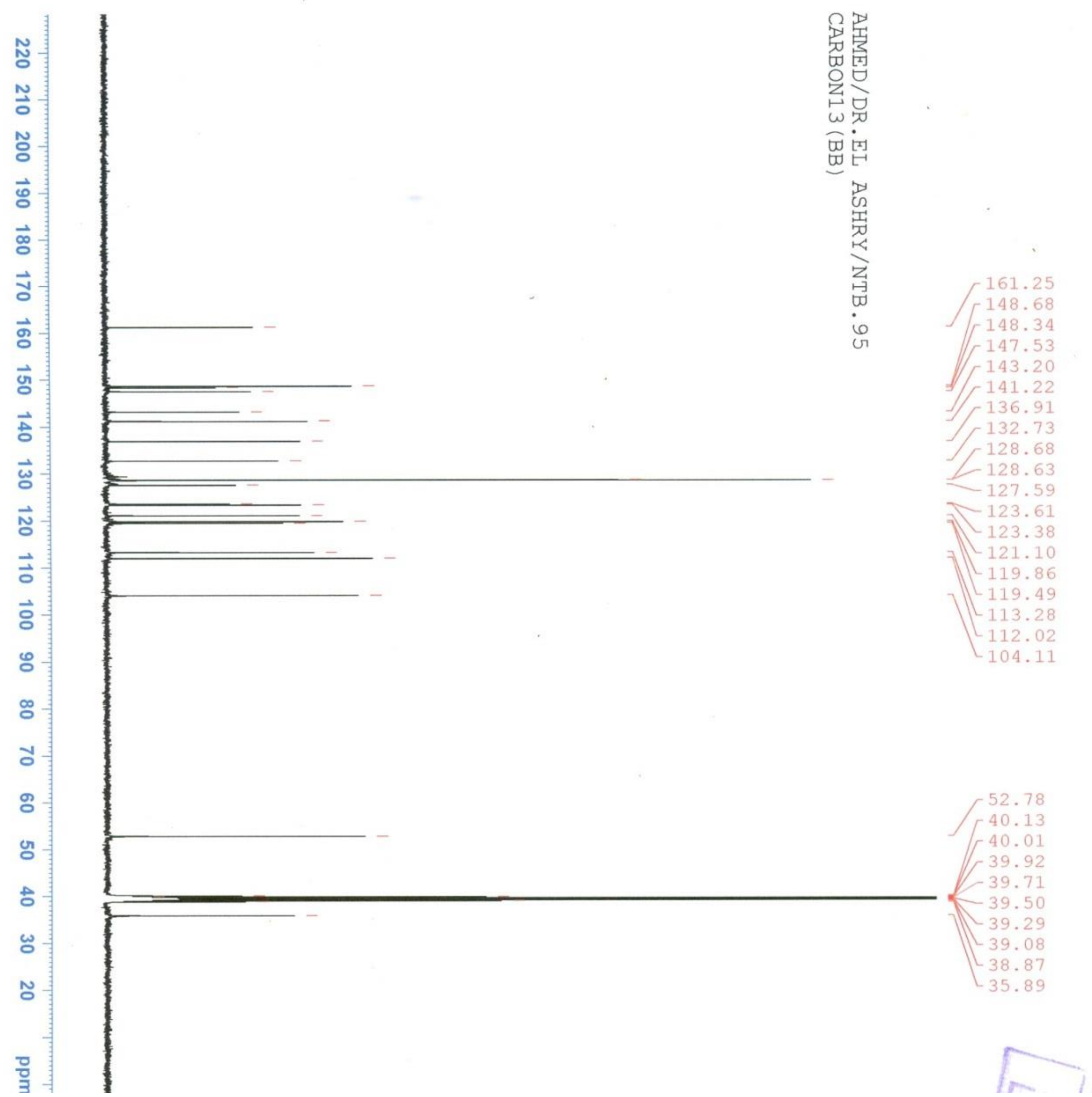

Figure S27. ^{13}C NMR of **4h**

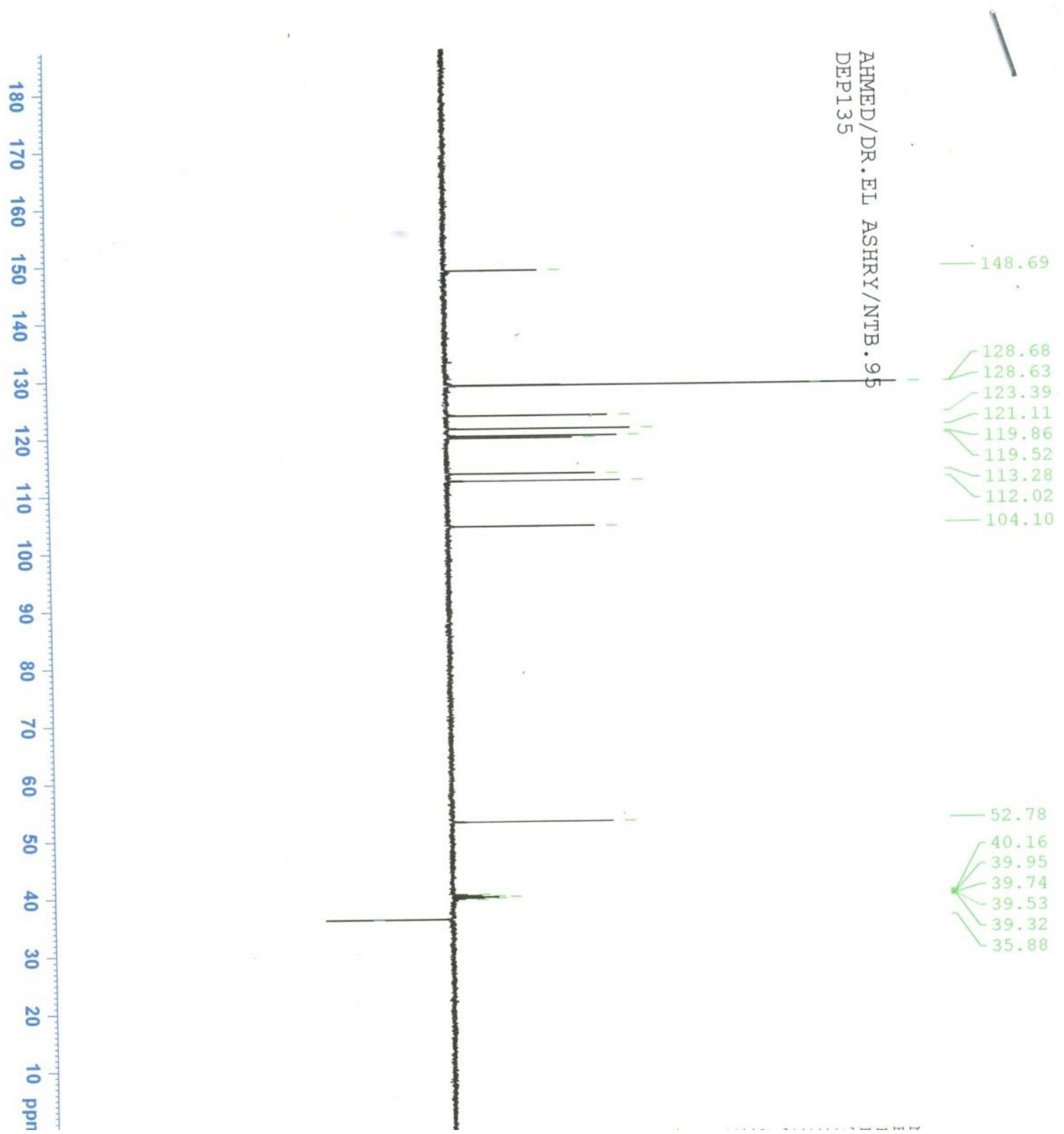

Figure S28. DEPT of **4h**

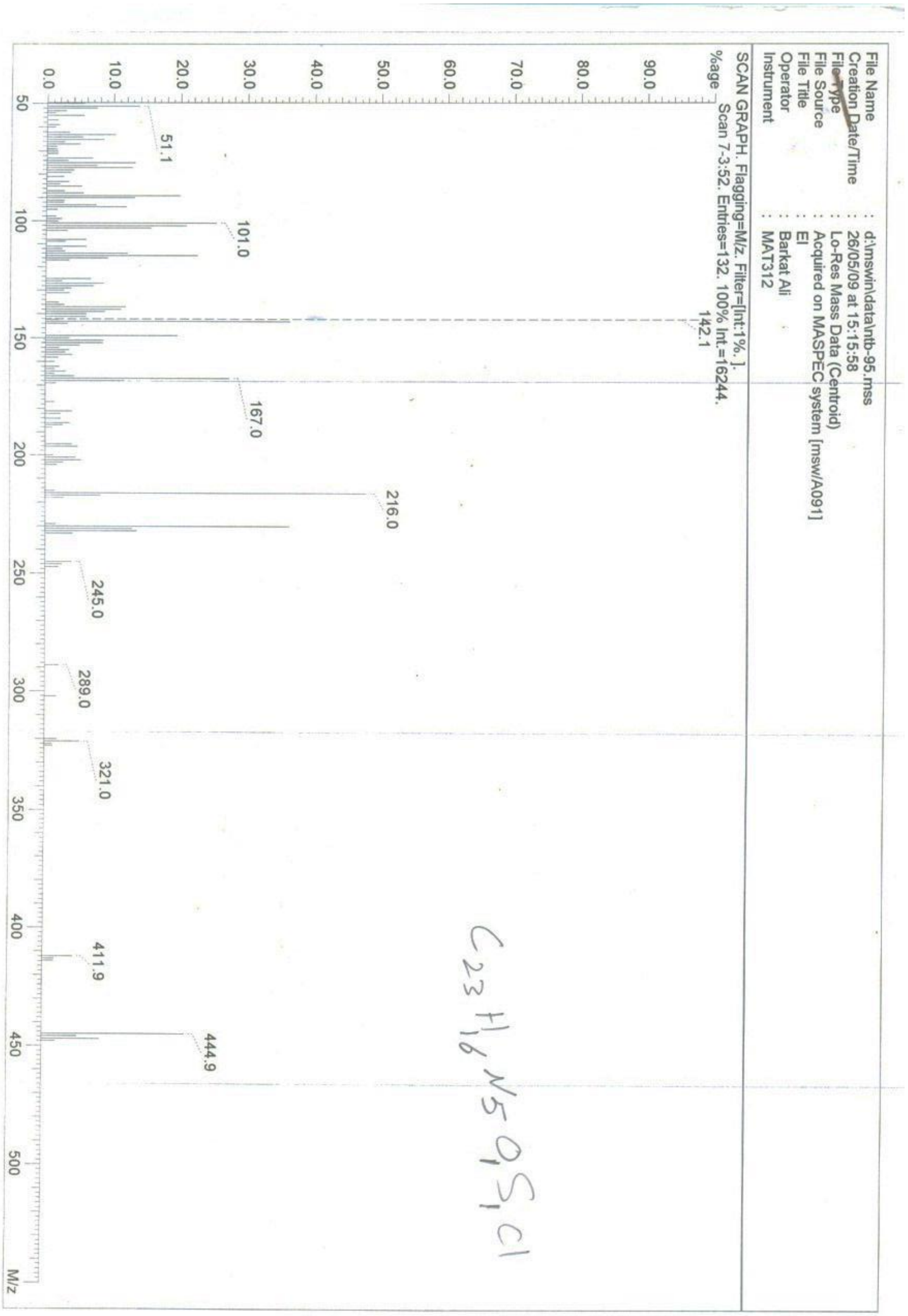

Figure S29. EIMS of **4h**

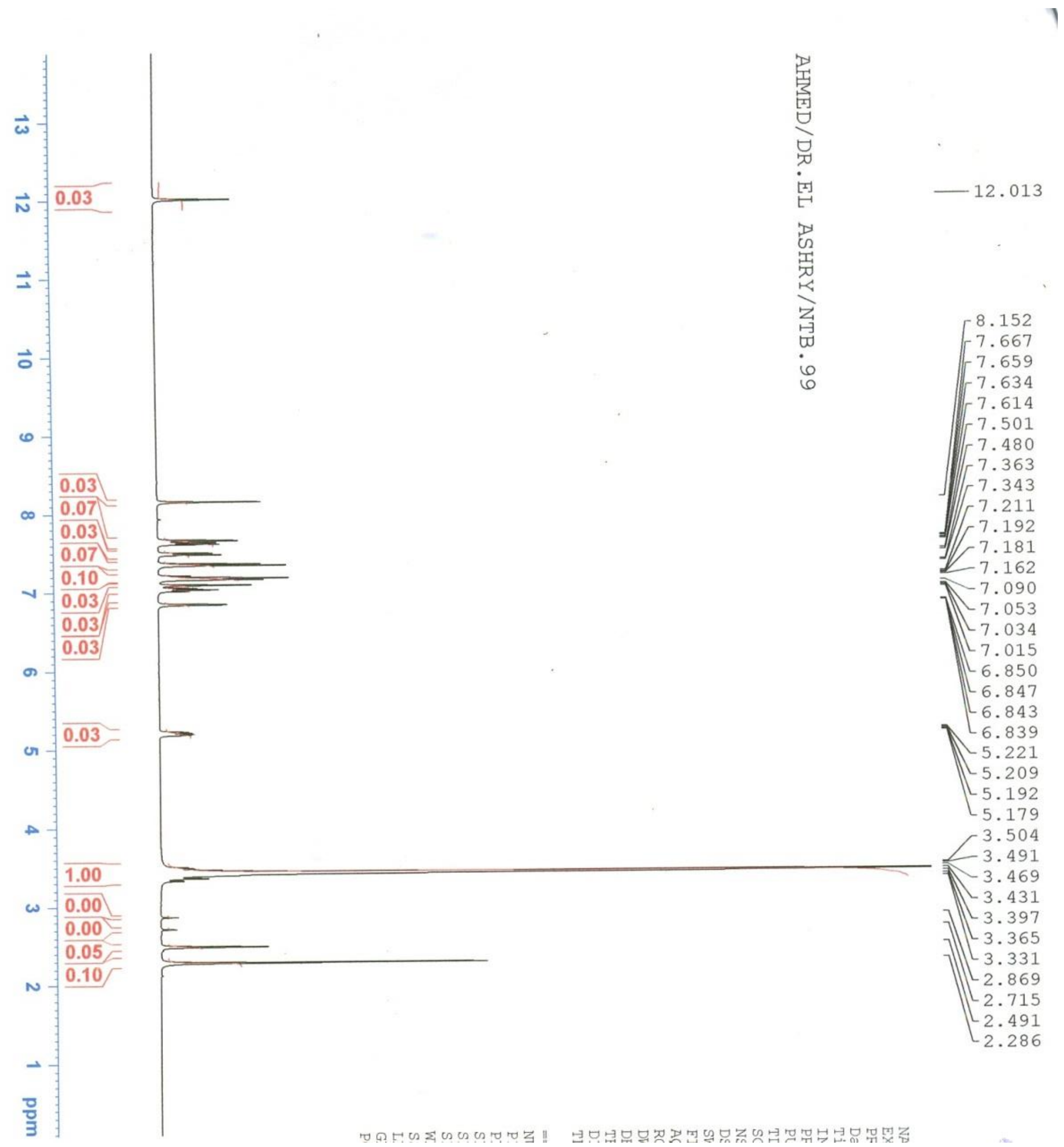

Figure S30. ^{1}H NMR of **4i**

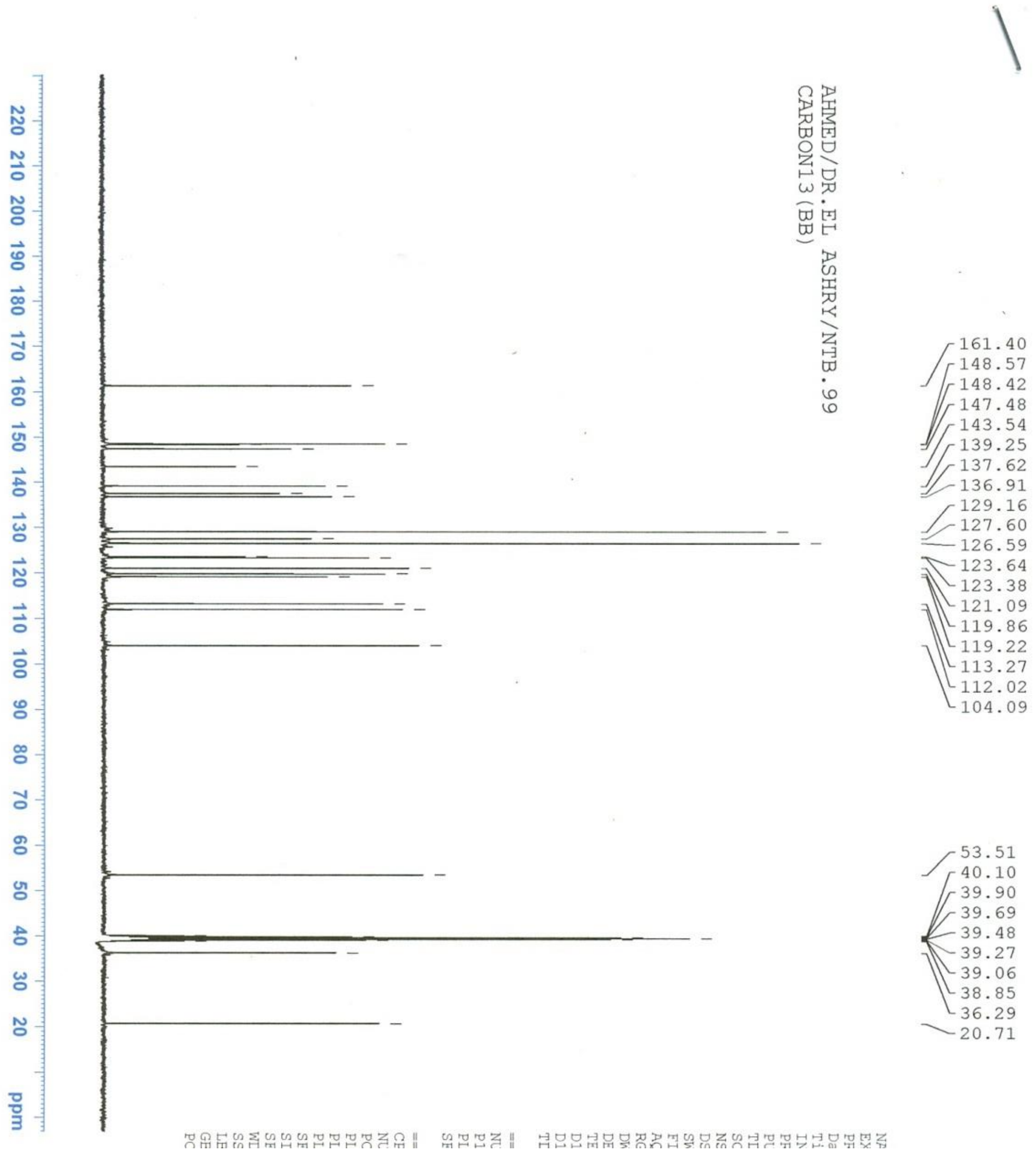

Figure S31. ^{13}C NMR of **4i**

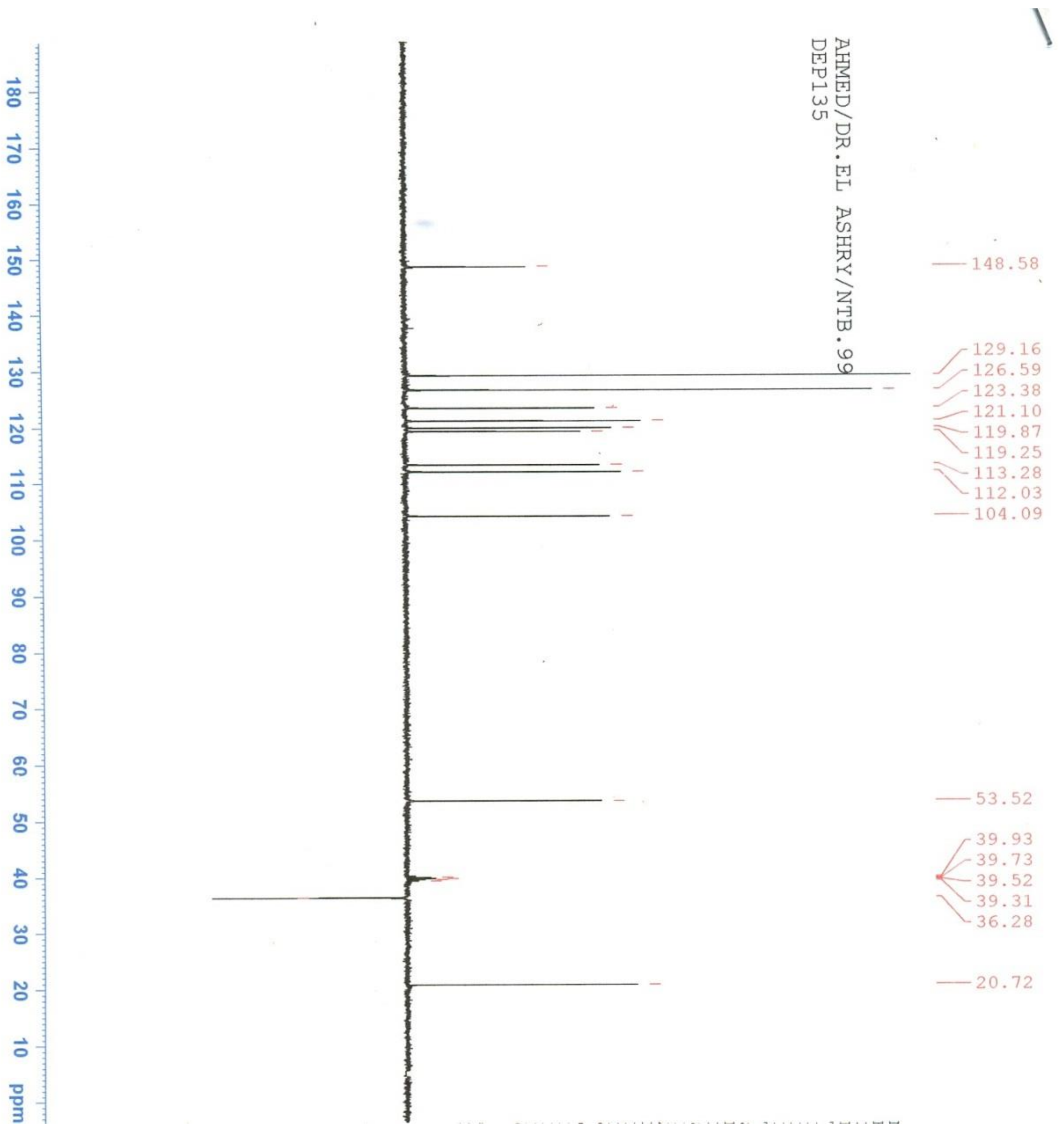

Figure S32. DEPT of **4i**

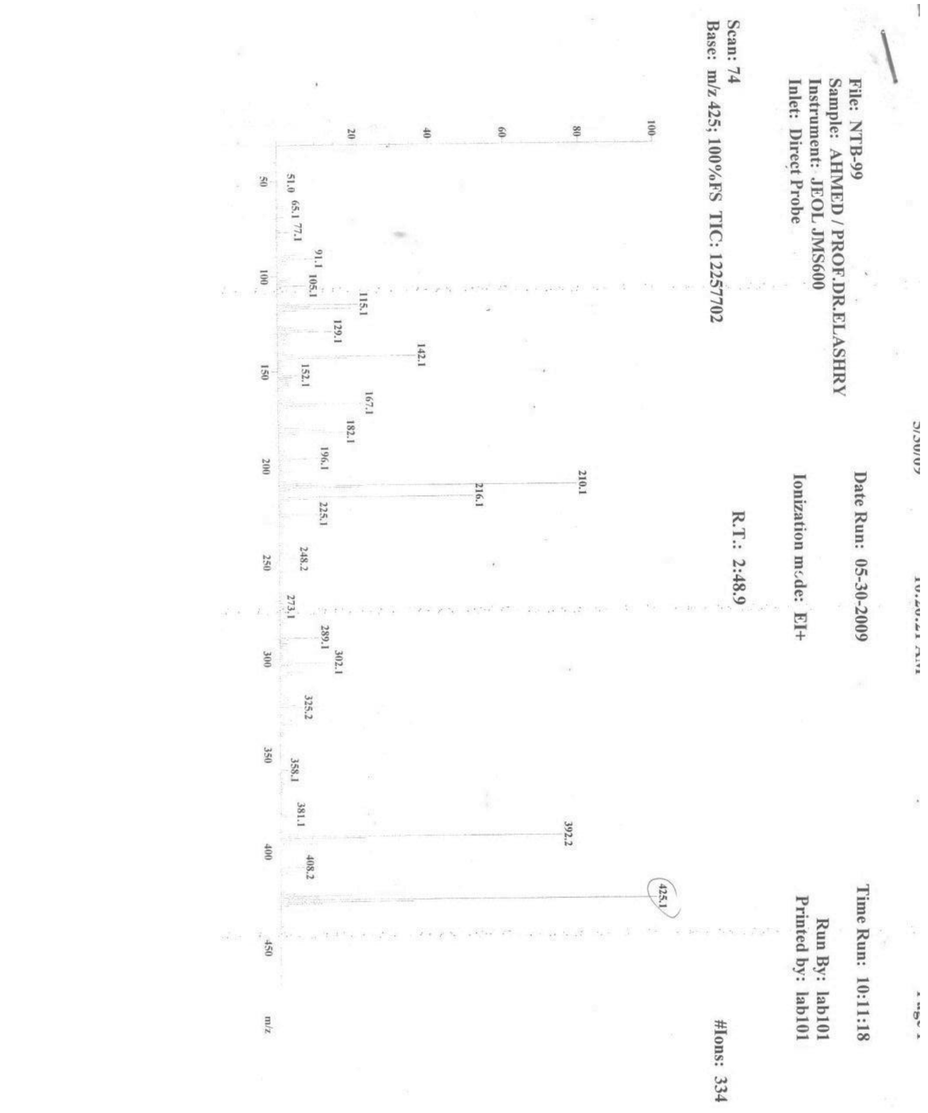

Figure S33. EIMS of **4i**

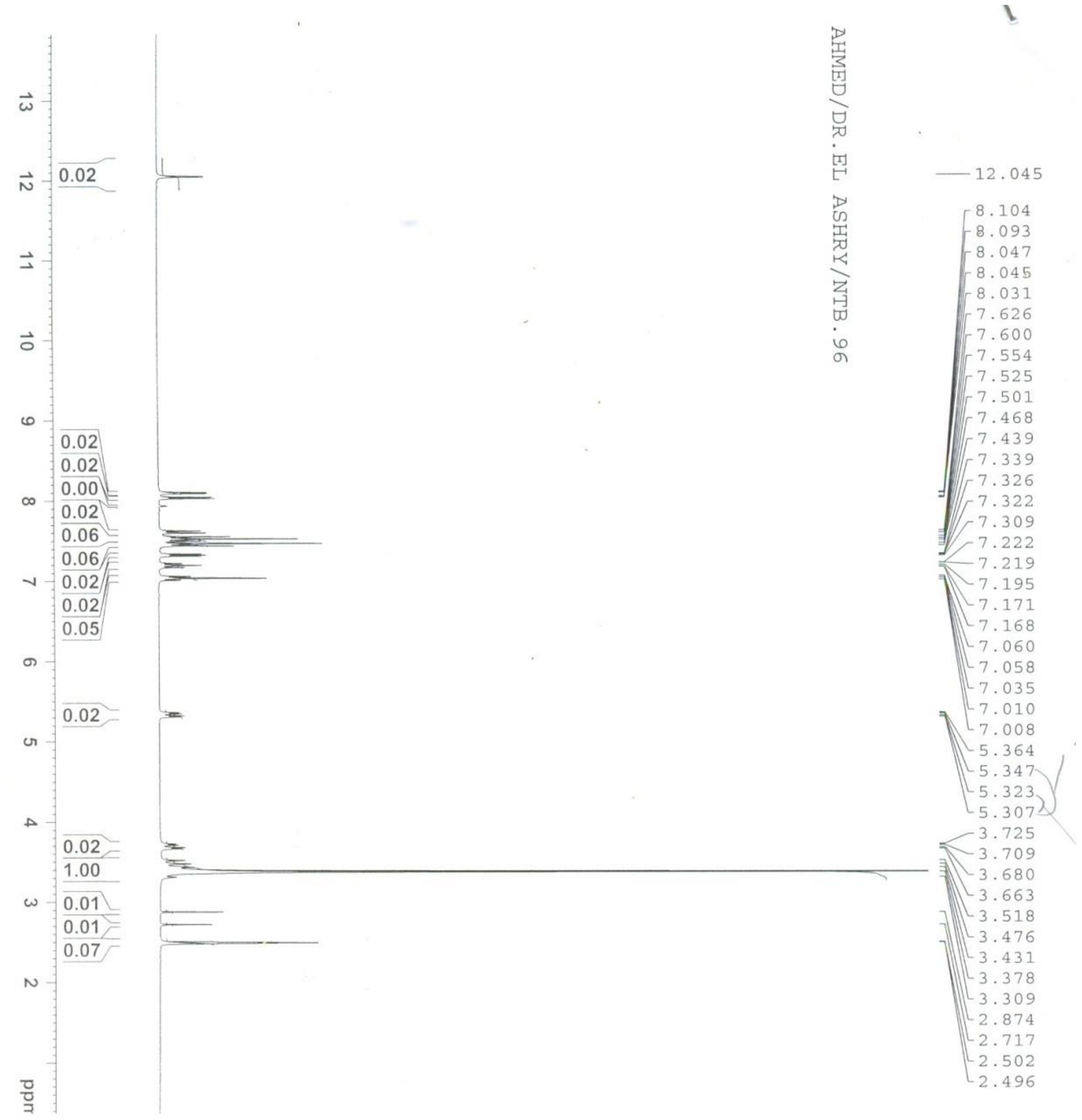

Figure S34. ^{1}H NMR of **4j**

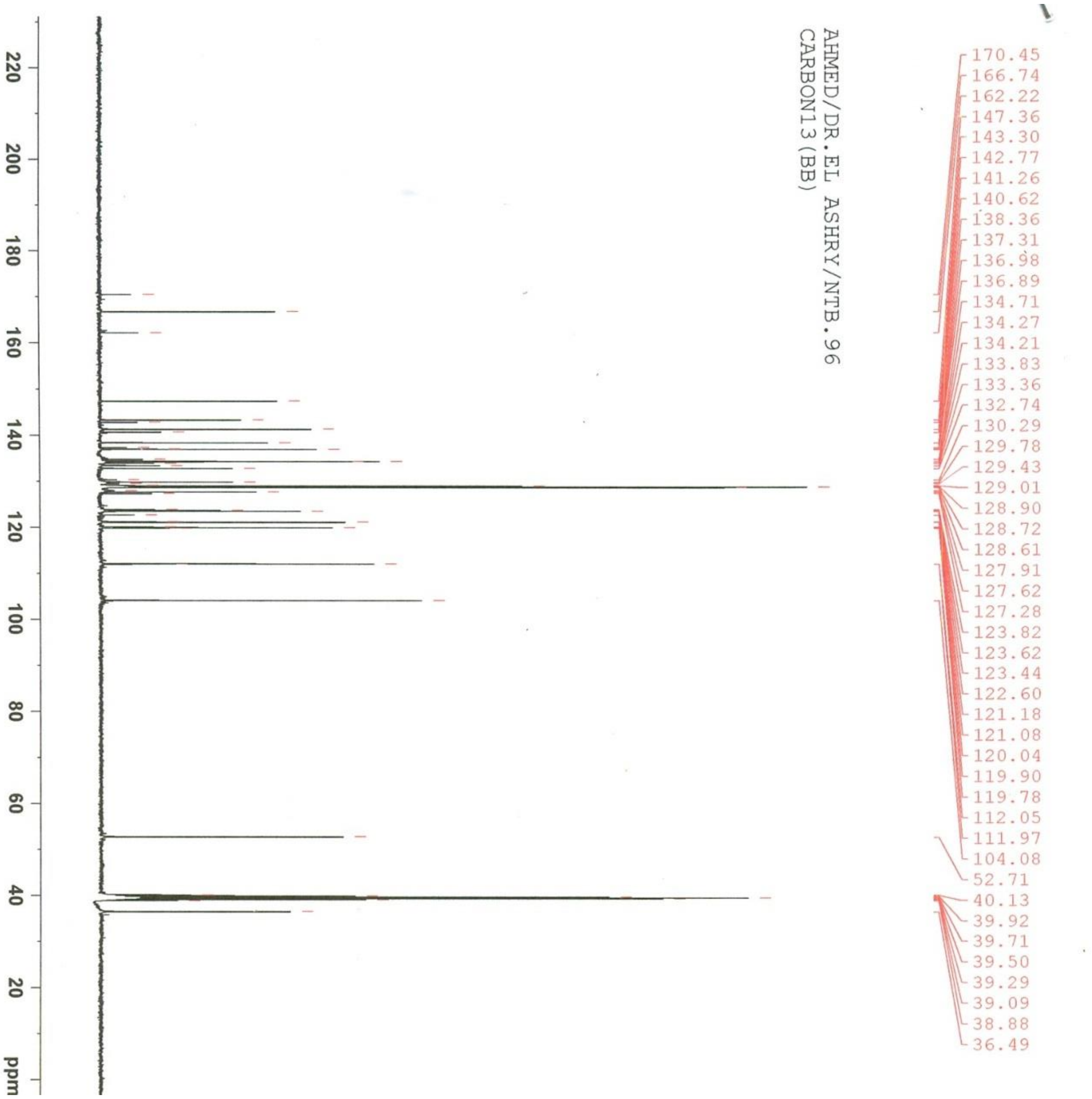

Figure S35. ^{13}C NMR of **4j**

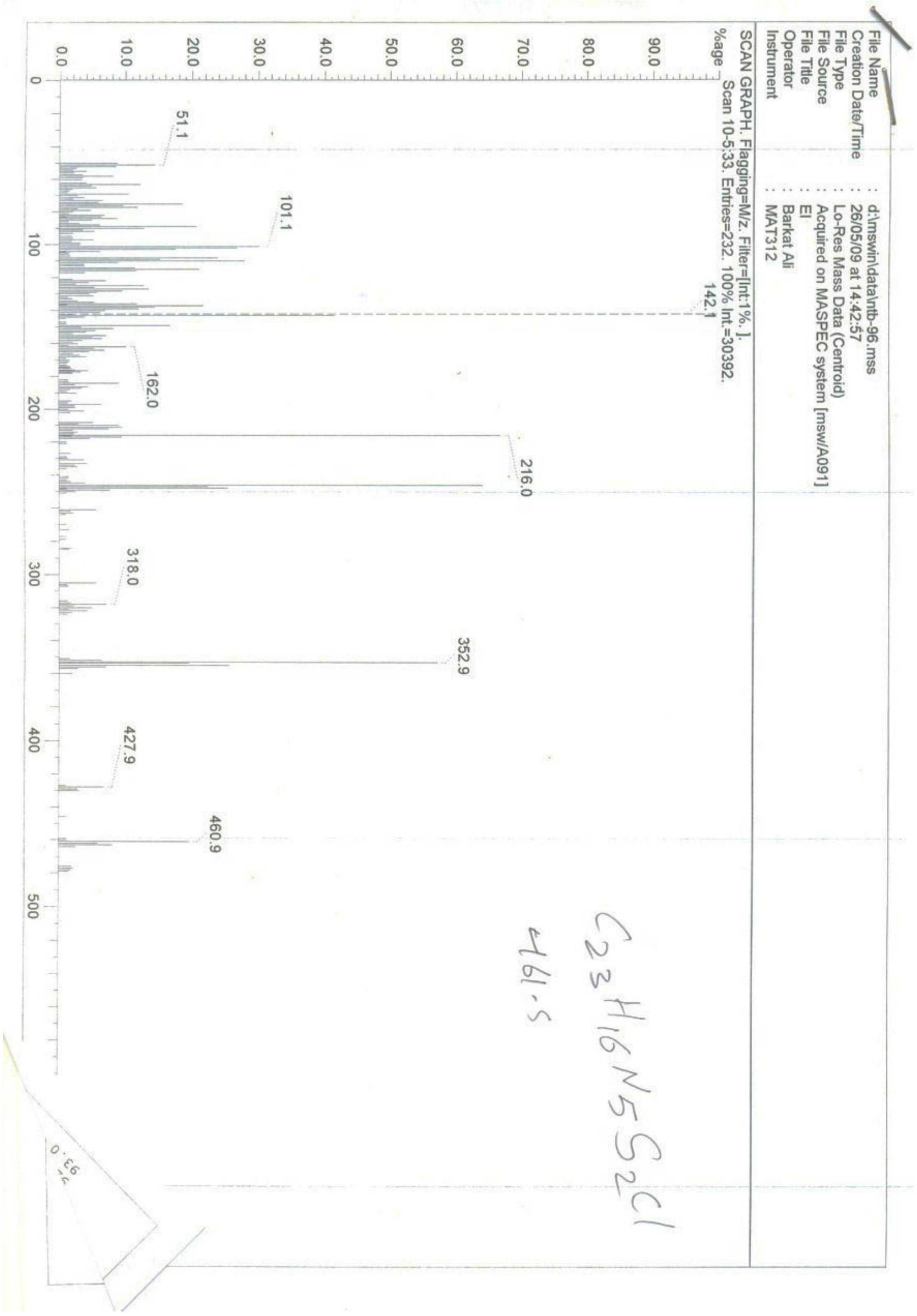

Figure S36. EIMS of **4j**

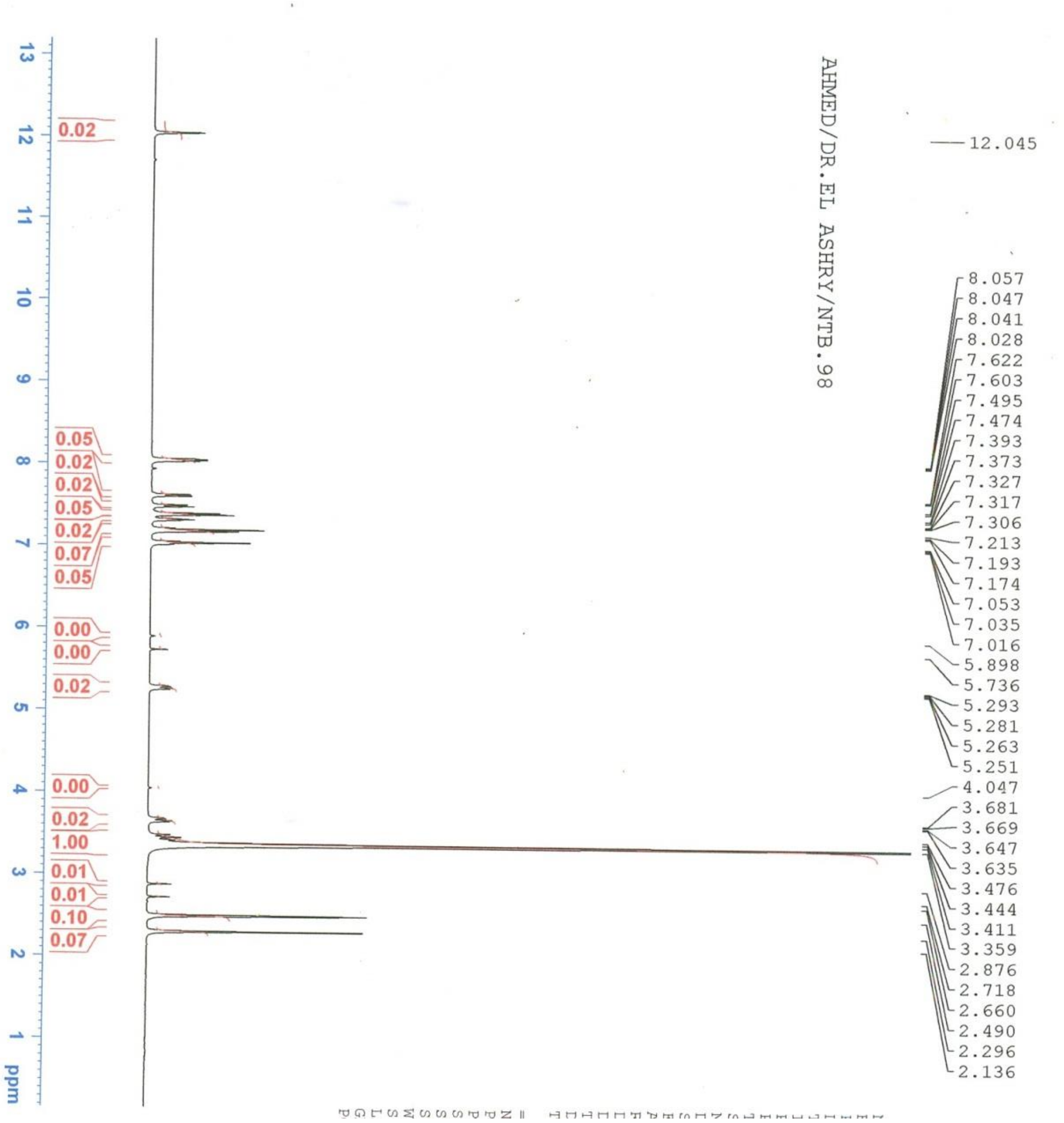

Figure S37. ^{1}H NMR of **4k**

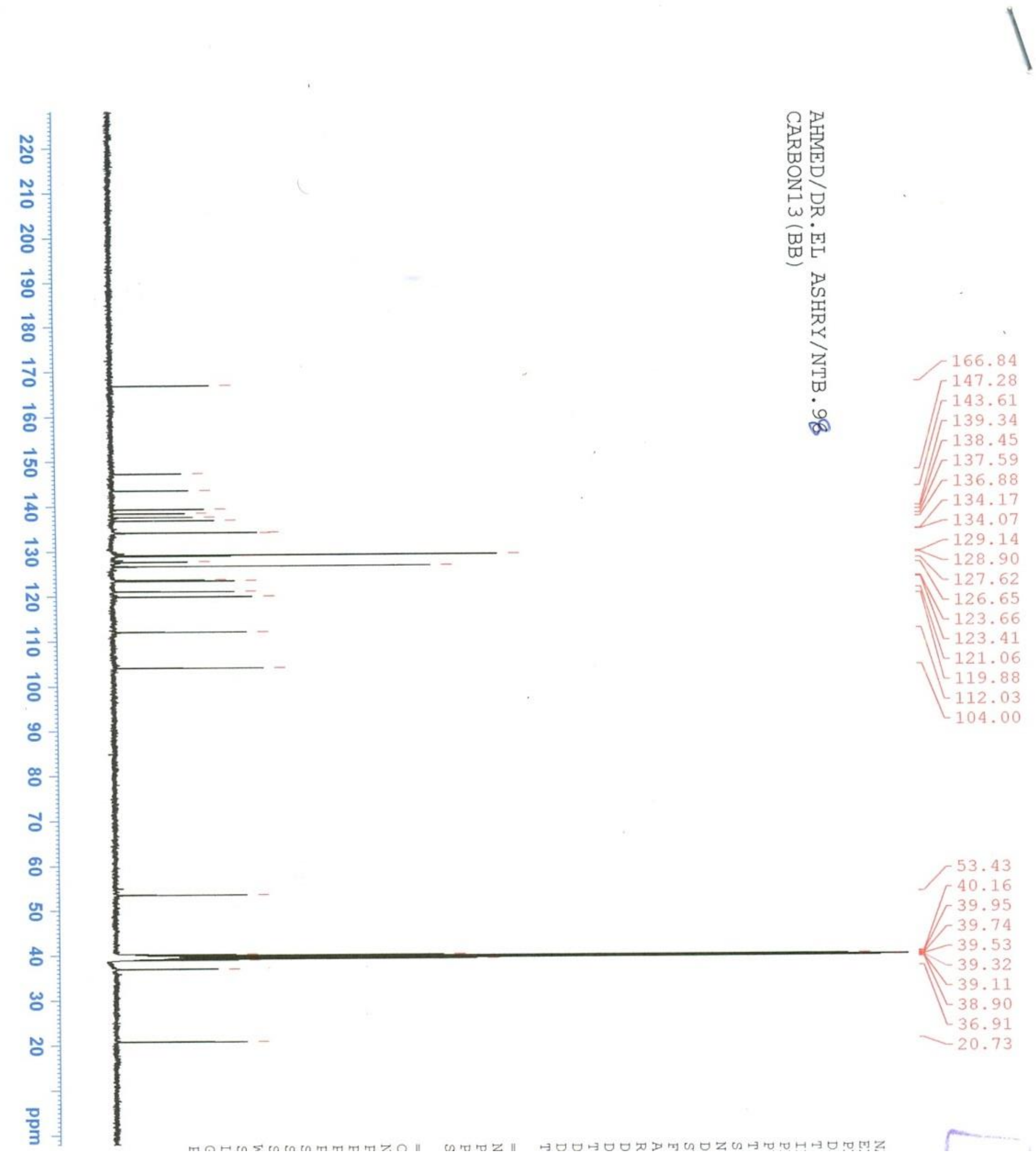

Figure S38. ^{13}C NMR of **4k**

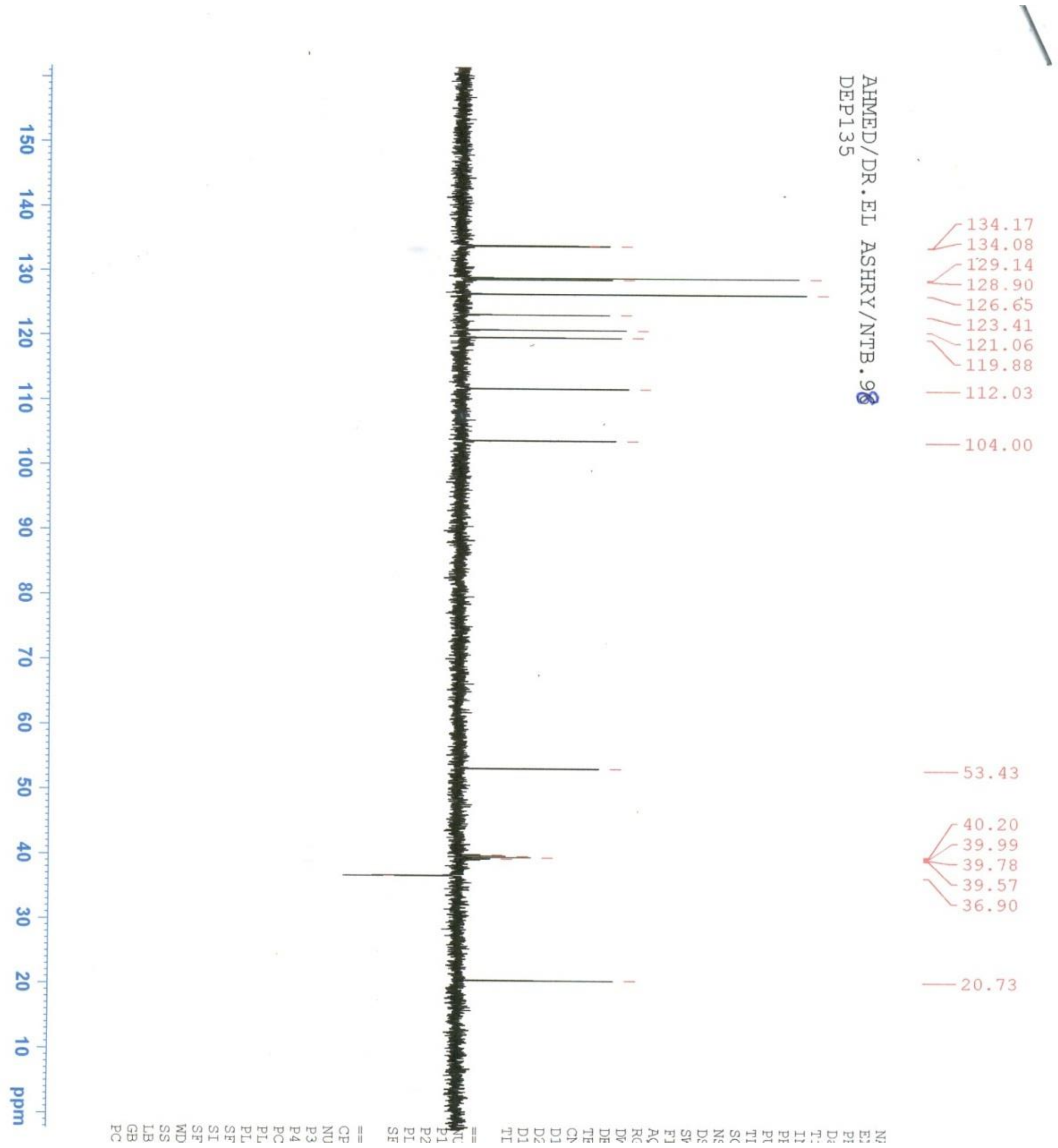

Figure S39. DEPT135 of **4k**

File: NTB-98
Sample: AHMED / PROF.DR.ELASHRY
Instrument: JEOL JMS600
Inlet: Direct Probe

Date Run: 05-30-2009

Ionization mode: EI+

Time Run: 10:26:51

Run By: lab101
Printed by: lab101

ican: 105
ase: m/z 226; 100%FS TIC: 12257925

R.T.: 4:0.5

#Ions: 377

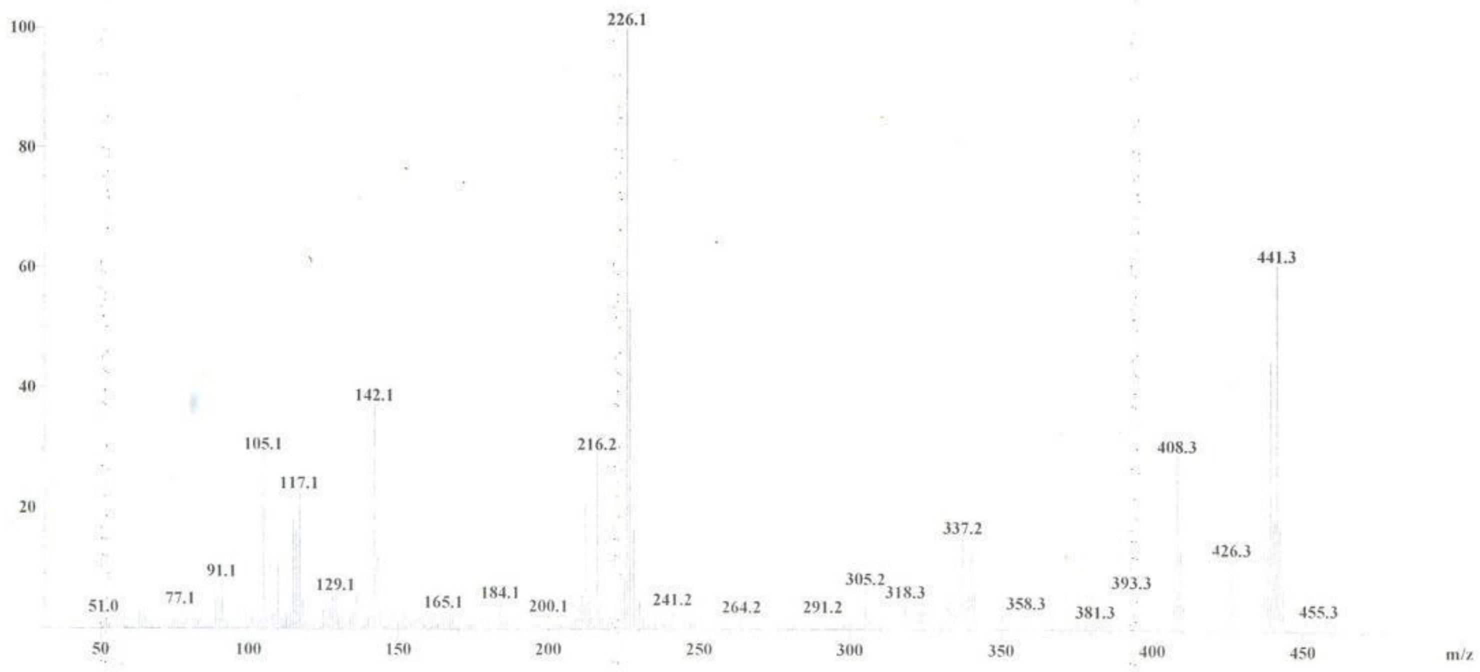

Figure S40. EIMS of **4k**

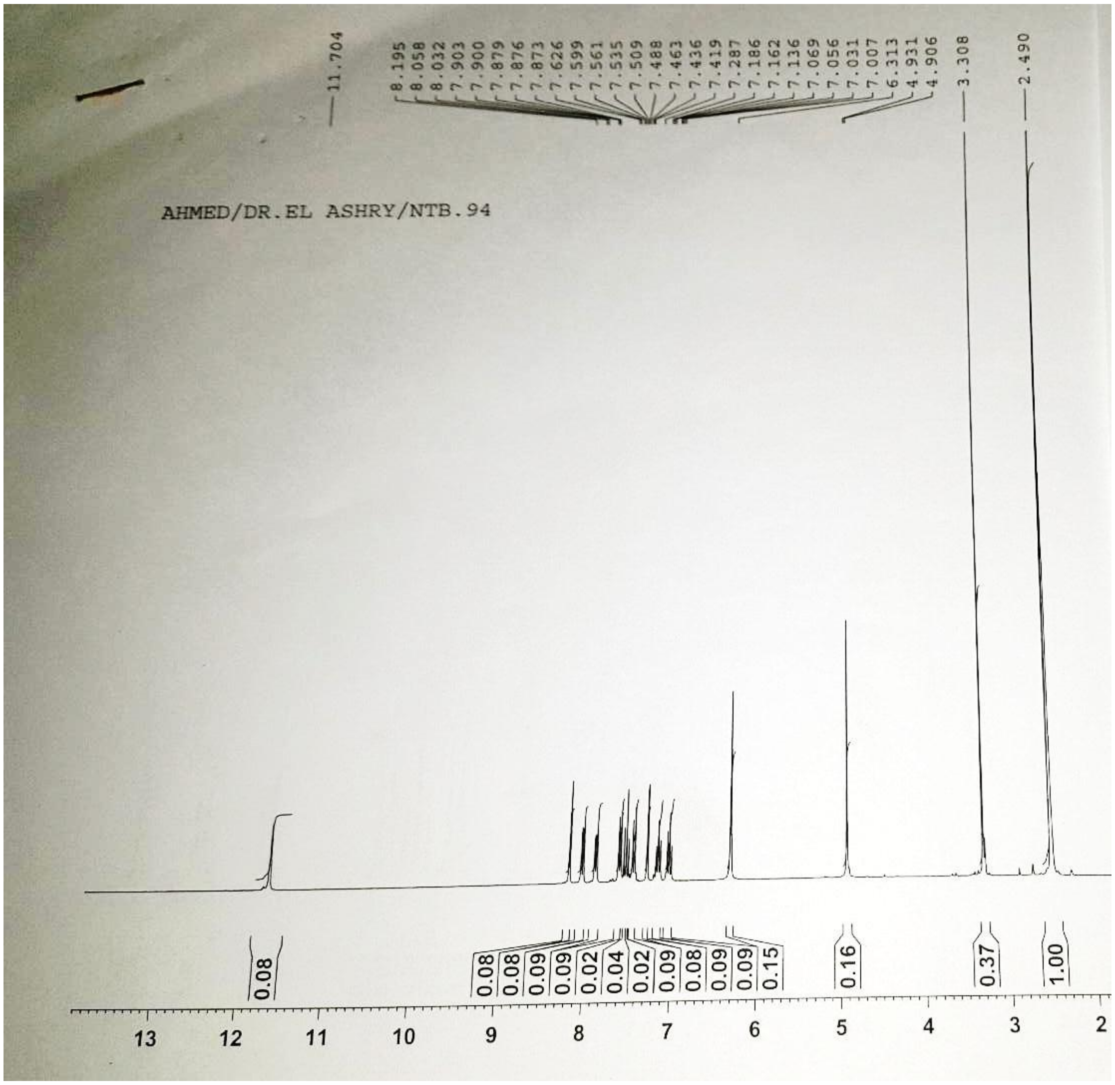

Figure S41. ^{1}H NMR of **7b**

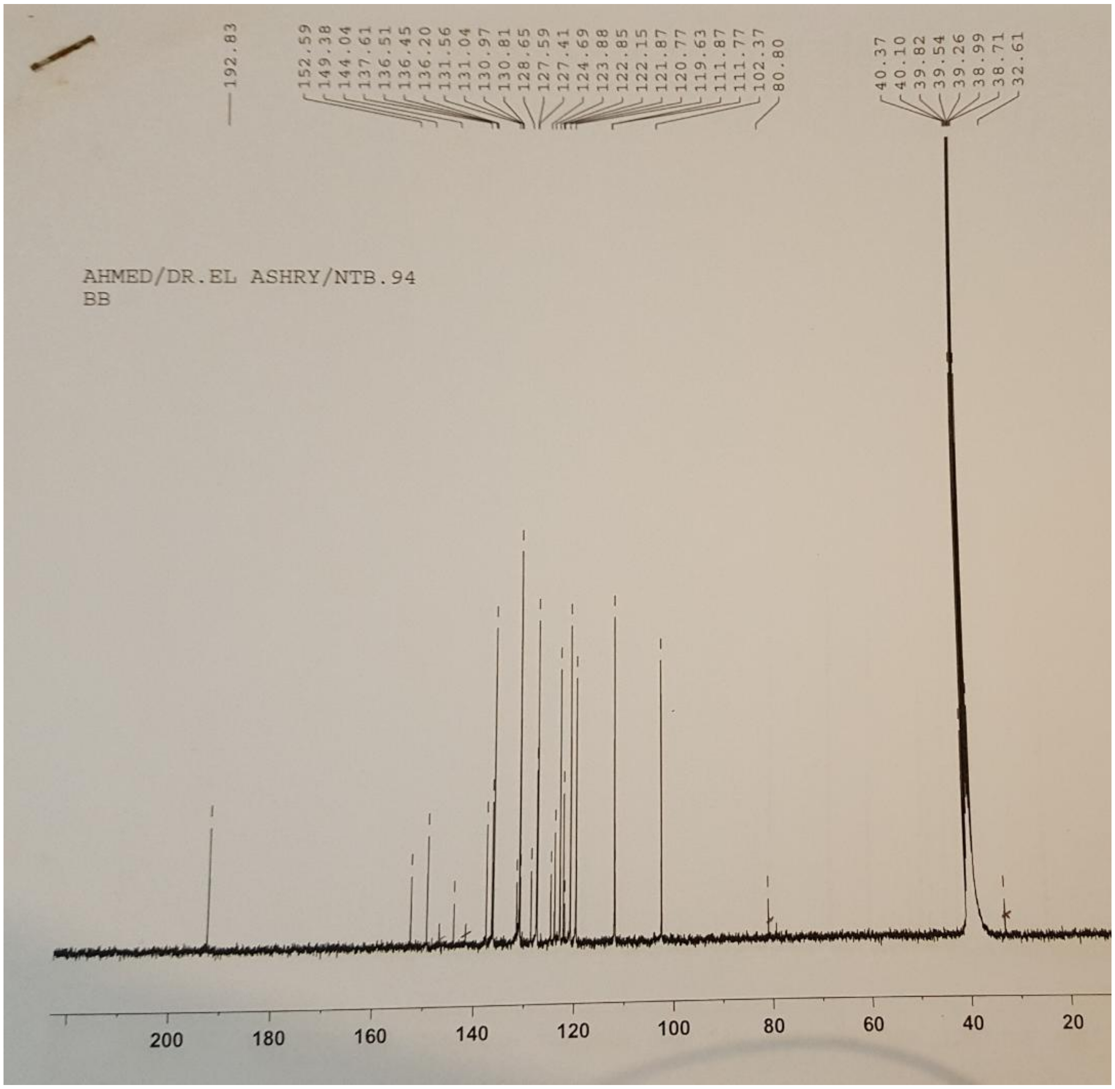

Figure S42. ^{13}C NMR of **7b**

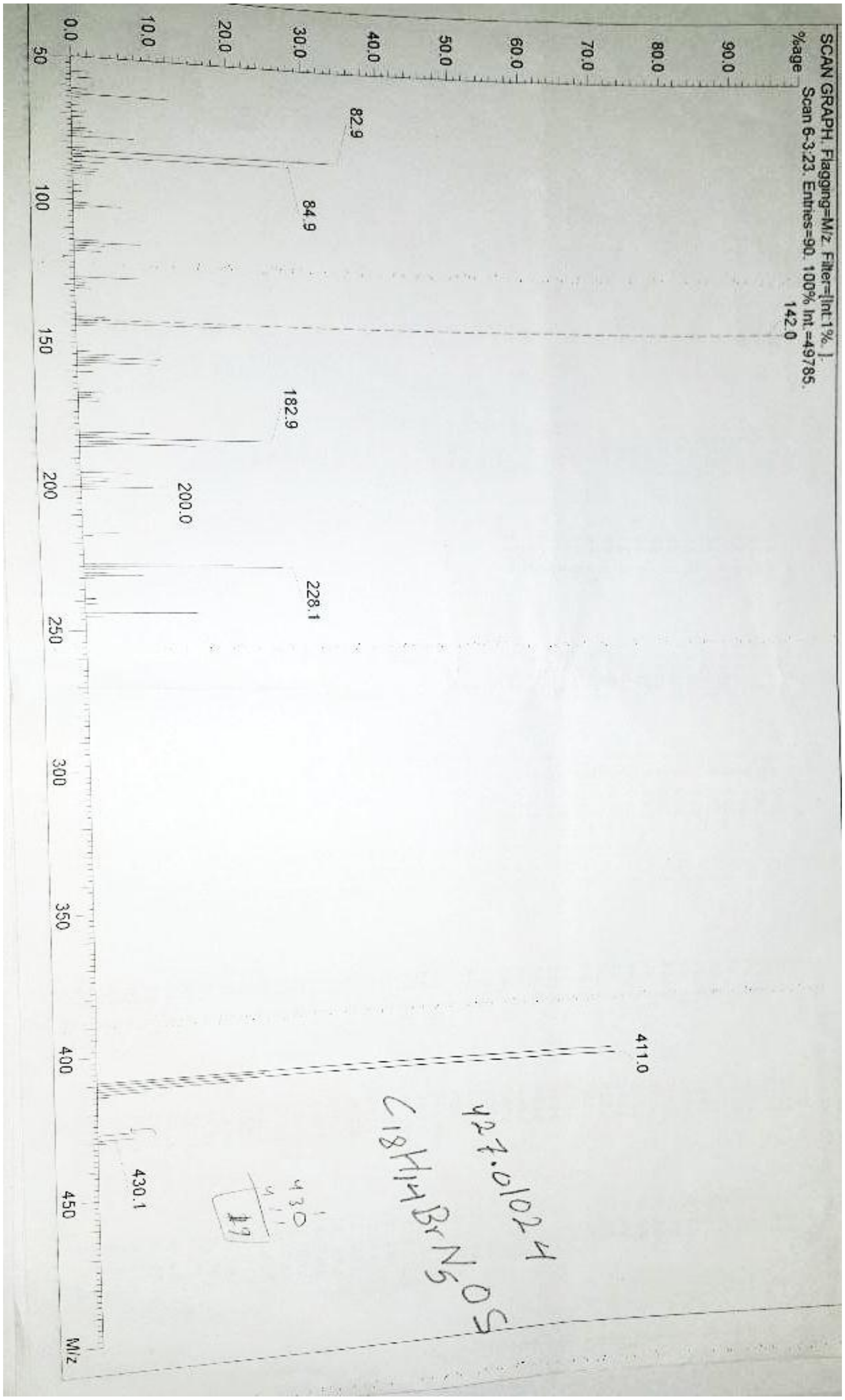

Figure S43. EIMS of **7b**

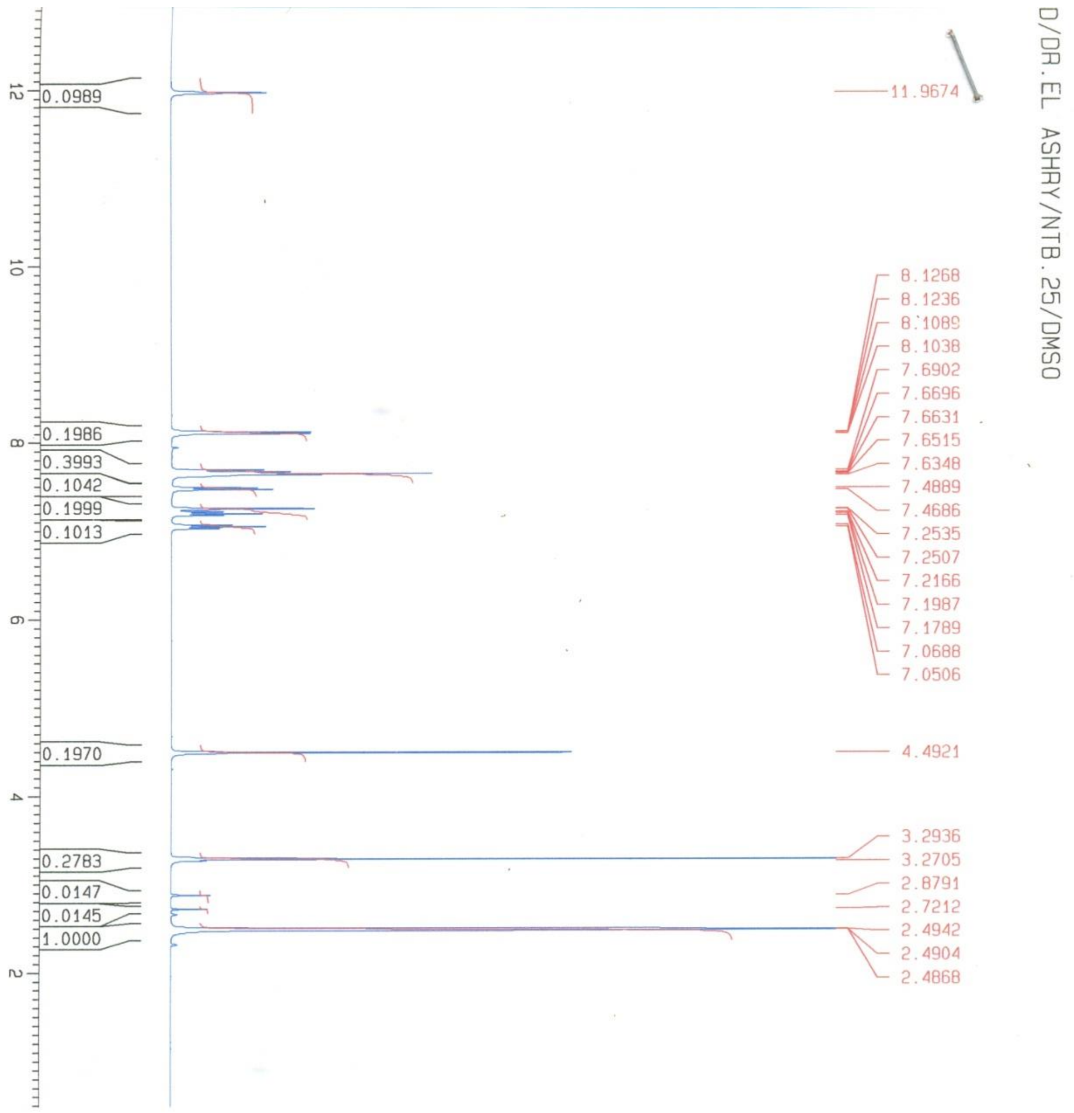

Figure S44. ^{1}H NMR of **8a**

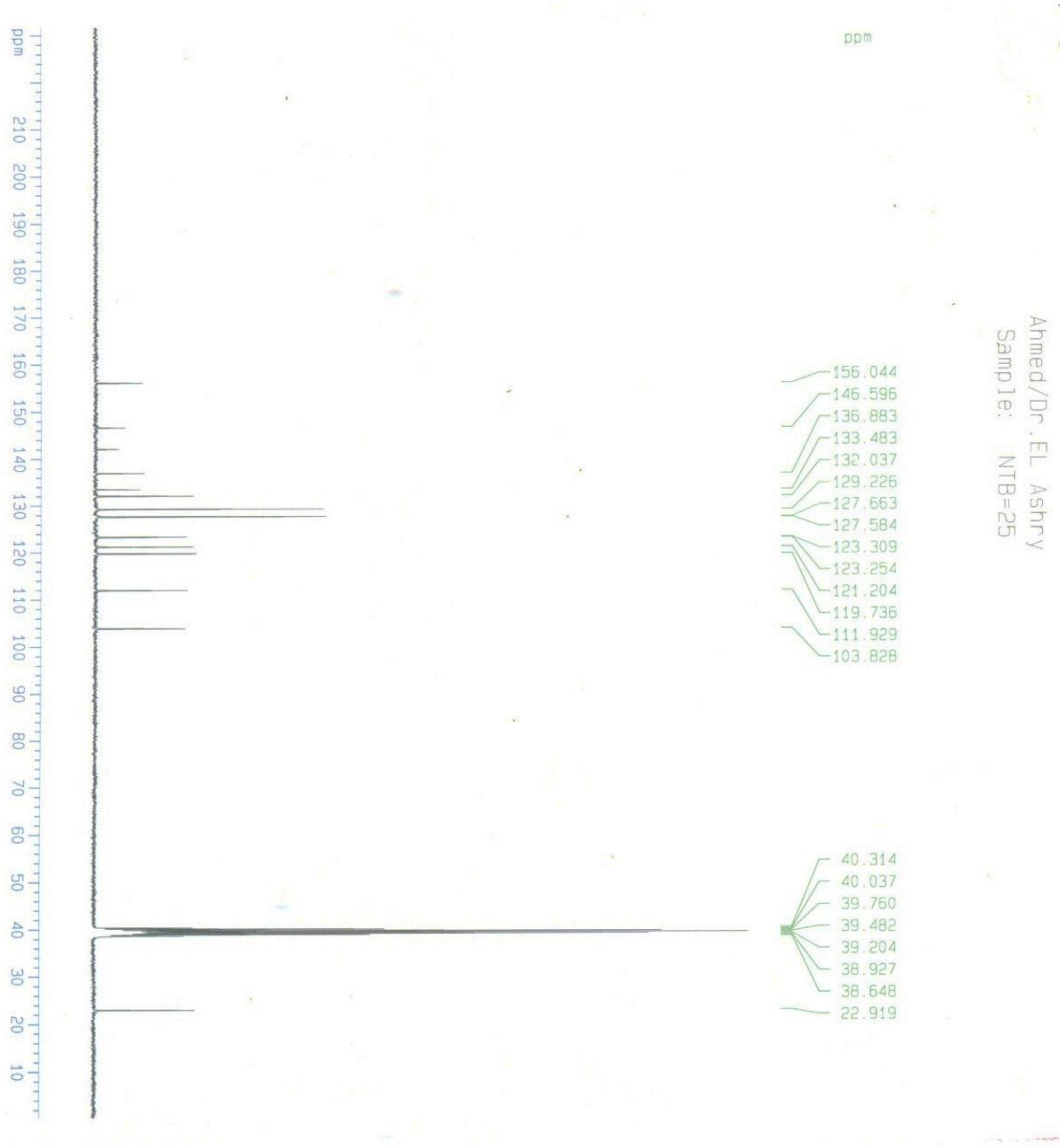

Figure S45. ^{13}C NMR of **8a**

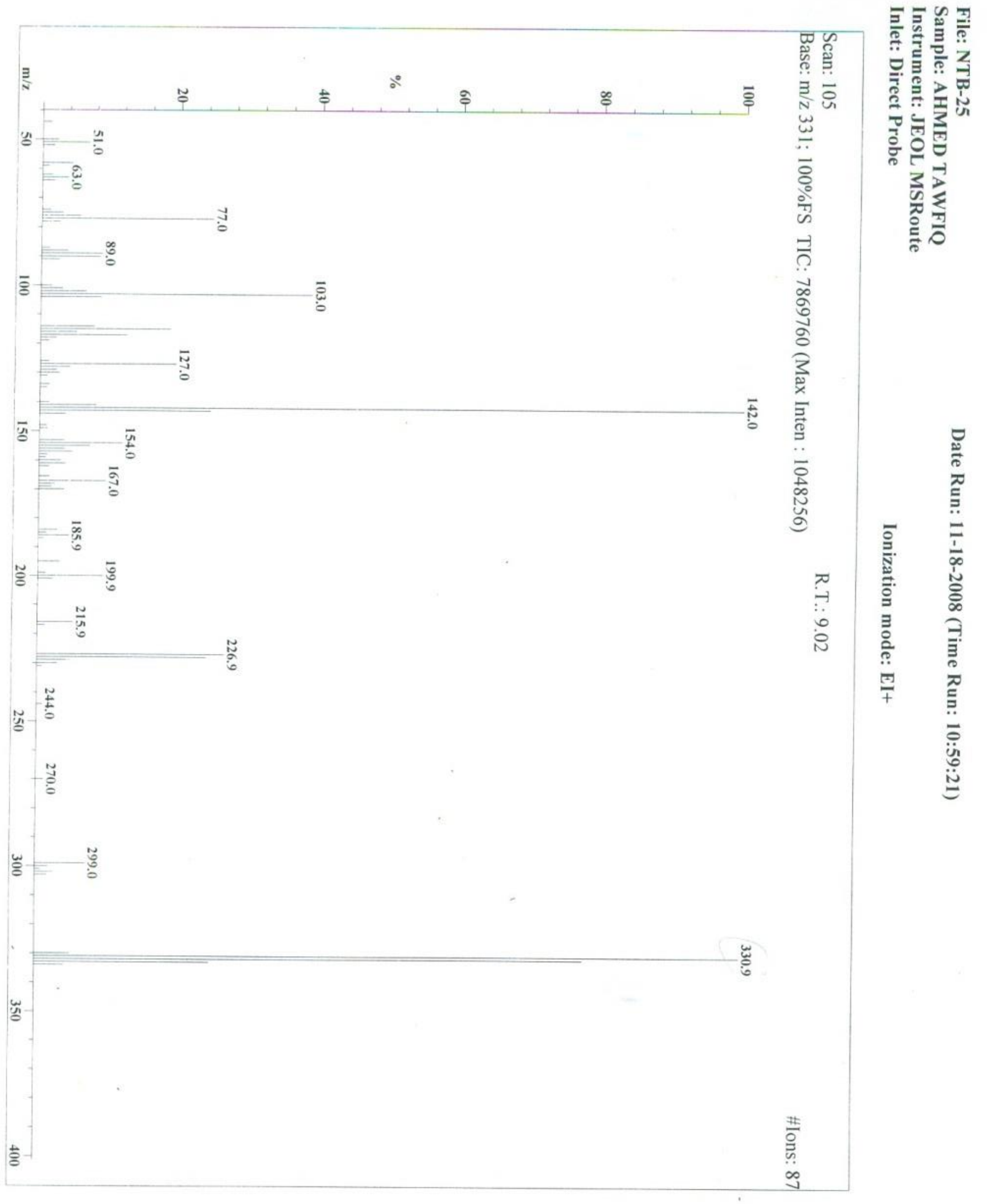

Figure S46. EIMS of **8a**

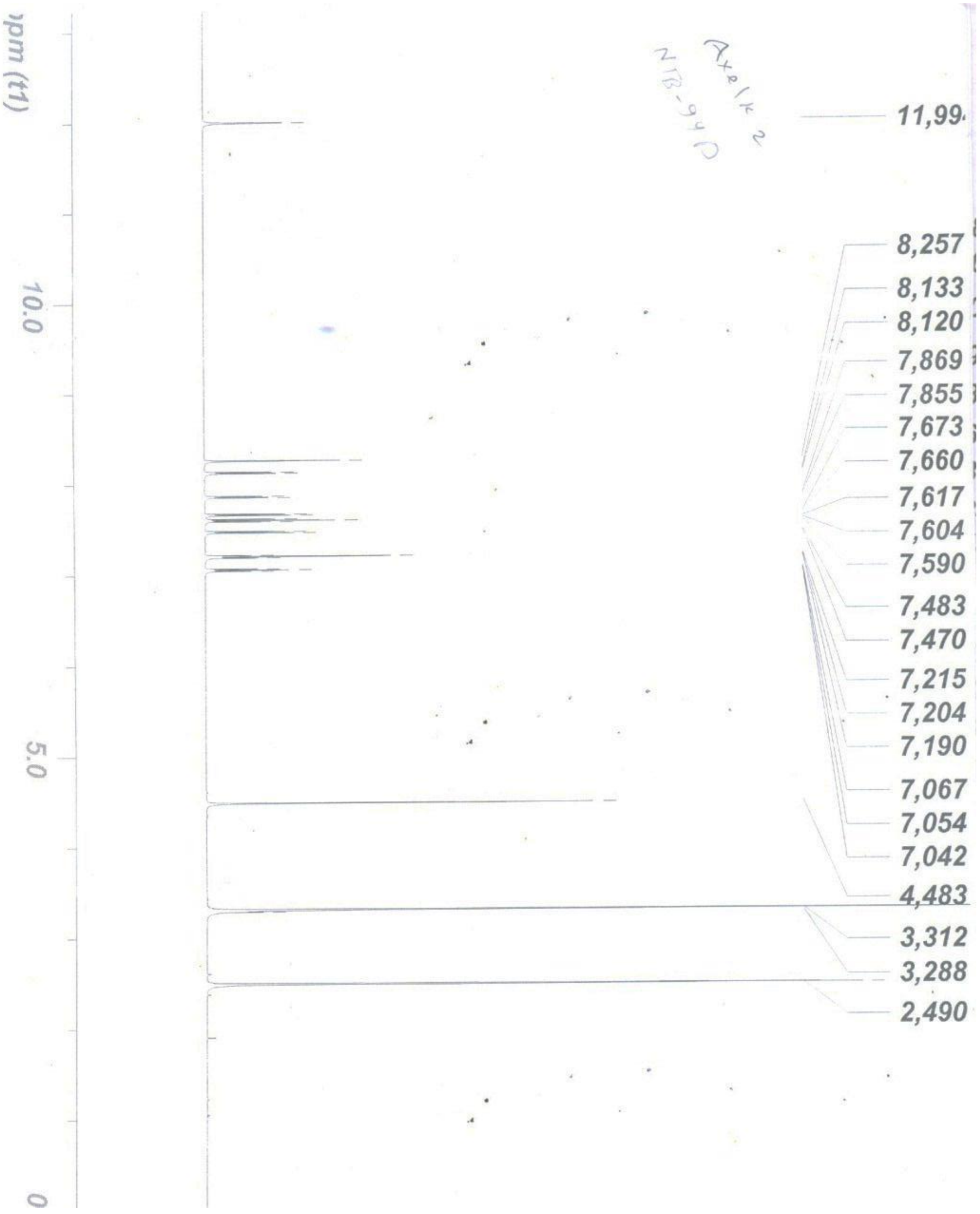

Figure S47. ^{1}H NMR of **8b**

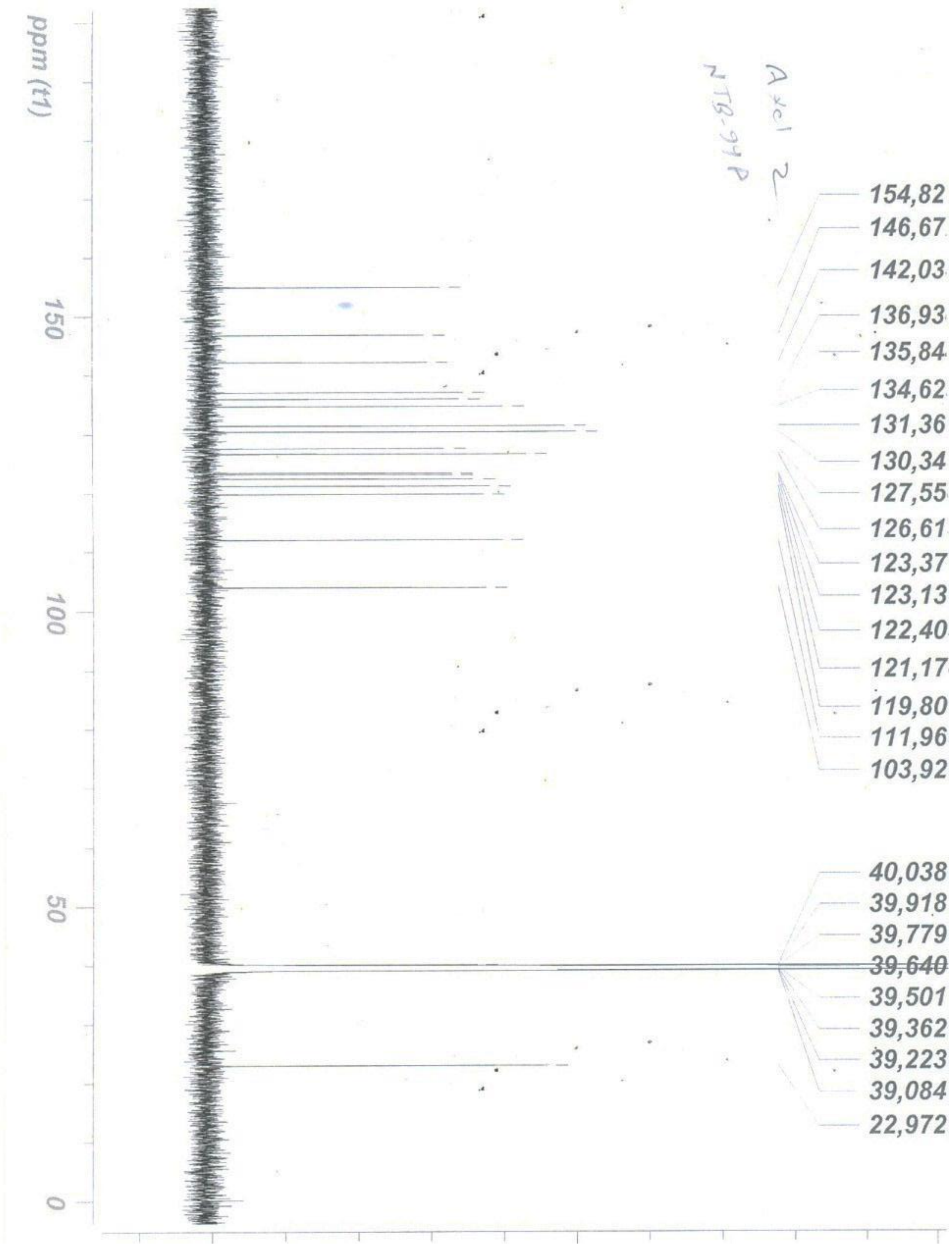

Figure S48. ^{13}C NMR of **8b**
